# Electrolyte Engineering for High-Voltage Lithium Metal Batteries

**DOI:** 10.34133/2022/9837586

**Published:** 2022-08-21

**Authors:** Liwei Dong, Shijie Zhong, Botao Yuan, Yuanpeng Ji, Jipeng Liu, Yuanpeng Liu, Chunhui Yang, Jiecai Han, Weidong He

**Affiliations:** ^1^MIIT Key Laboratory of Critical Materials Technology for New Energy Conversion and Storage, School of Chemistry and Chemical Engineering, Harbin Institute of Technology, Harbin 150080, China; ^2^National Key Laboratory of Science and Technology on Advanced Composites in Special Environments and Center for Composite Materials and Structures, Harbin Institute of Technology, Harbin 150080, China; ^3^State Key Laboratory of Urban Water Resource and Environment, Harbin Institute of Technology, Harbin 150080, China; ^4^Chongqing Research Institute, Harbin Institute of Technology, Chongqing 401151, China; ^5^School of Mechanical Engineering, Chengdu University, Chengdu, 610106, China

## Abstract

High-voltage lithium metal batteries (HVLMBs) have been arguably regarded as the most prospective solution to ultrahigh-density energy storage devices beyond the reach of current technologies. Electrolyte, the only component inside the HVLMBs in contact with both aggressive cathode and Li anode, is expected to maintain stable electrode/electrolyte interfaces (EEIs) and facilitate reversible Li^+^ transference. Unfortunately, traditional electrolytes with narrow electrochemical windows fail to compromise the catalysis of high-voltage cathodes and infamous reactivity of the Li metal anode, which serves as a major contributor to detrimental electrochemical performance fading and thus impedes their practical applications. Developing stable electrolytes is vital for the further development of HVLMBs. However, optimization principles, design strategies, and future perspectives for the electrolytes of the HVLMBs have not been summarized in detail. This review first gives a systematical overview of recent progress in the improvement of traditional electrolytes and the design of novel electrolytes for the HVLMBs. Different strategies of conventional electrolyte modification, including high concentration electrolytes and CEI and SEI formation with additives, are covered. Novel electrolytes including fluorinated, ionic-liquid, sulfone, nitrile, and solid-state electrolytes are also outlined. In addition, theoretical studies and advanced characterization methods based on the electrolytes of the HVLMBs are probed to study the internal mechanism for ultrahigh stability at an extreme potential. It also foresees future research directions and perspectives for further development of electrolytes in the HVLMBs.

## 1. Introduction

**Figure 1:**
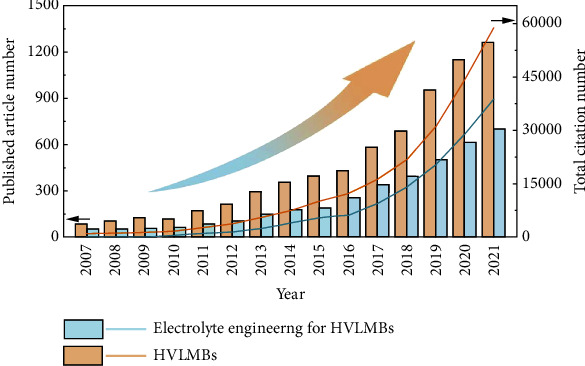
The published article number based on HVLMBs and electrolyte engineering for HVLMBs from 2007 to 2021 (accessed April 27, 2022, Web of Science).

With the continuously increasing demand for portable electronics and electric vehicles, higher requirements have been placed on rechargeable batteries. Due to the zero memory effect and long cycle life, lithium- (Li-) ion batteries (LIBs) composed of graphite anode and LiFePO_4_, LiCoO_2_ (LCO), or LiNi_*x*_Mn_*y*_Co_1‐*x*‐*y*_O_2_ (NMC) cathodes play an irreplaceable role in almost every aspect of our life [1–7]. However, the energy density of LIBs can hardly exceed the upper limit of 300 Wh kg^−1^ [8, 9]. By contrast, Li metal becomes the preferred anode for high-energy-density cells for its ultralow redox potential (-3.040 V versus the standard hydrogen electrode) and incomparable theoretical capacity (3862 mAh g^−1^) [6, 10, 11]. As Li anode is used instead of a graphite anode, the energy density increases by ~50% compared to conventional LIBs [12, 13]. Compared with conversion cathodes, intercalation cathodes own more complete production technology and higher work potential [14–16]. High-voltage Li metal batteries (HVLMBs) composed of intercalation cathodes and Li anode at high potential can provide energy density close to 400 Wh kg^−1^ and even higher [17, 18], which is honored as a promising next-generation battery system and attracts the rapidly growing interest of extensive research (Figure 1).

As known, HVLMBs are composed of four parts: high-voltage cathode, Li anode, separator, and electrolyte [19, 20]. Owing to the catalytic nature of high-voltage cathodes and the infamous reactivity of Li anode, the electrolyte directly contacting with the two electrodes is particularly instrumental in maintaining the stability of the entire battery system [21–23]. Moreover, electrochemical reactions at the electrode/electrolyte interphases are triggered during the charging/discharging with the generation of Li anode-side solid electrolyte interphase (SEI) and the cathode electrolyte interphase (CEI) counterpart as a result of the initial reduction and oxidation of electrolyte, respectively [24, 25]. The interphases generated in the electrolyte are expected to be robust enough to obstruct the continued side reactions between the electrolyte and the electrodes. Furthermore, the electrolyte should be endowed with rapid Li^+^ transport, sufficient wetting to the separator, and low flammability to ensure the excellent rate performance and safety of HVLMBs.

Carbonate-based electrolyte, which is widely utilized in commercial LIBs, shows high antioxidation stability of ~4.3 V. Nonetheless, its poor compatibility with Li anode brings about severe Li dendrite growth and low Li plating/stripping Coulombic efficiency (CE) [26]. Ether-based electrolyte exhibits exceptional stability with Li metal, as evidenced by elevated CE of ~95%, while oxidation appears above 4 V [27]. When they are applied to practical HVLMBs, consequently, they tend to decompose accompanied by the release of O_2_ and CO_2_ at a lower potential [28, 29]. Besides, although the SEI layers induced by traditional carbonate- and ether-based electrolytes play a certain role in protecting Li anode, their loose morphology and nonuniform distribution fail to suppress the dendrite growth, resulting in inferior lifespan and safety [30, 31]. On the other hand, the CEI layers generated in traditional electrolytes are mainly unstable organic carbides and hence fail to inhibit the continuous electrolyte oxidation and the dissolution of transition metal at an extreme potential [32, 33]. Therefore, developing stable electrolytes is vital for further promotions and applications of the HVLMBs. Electrolytes account for more than half of the total published articles on HVLMBs each year, as displayed in Figure 1. Extensive optimization strategies and design principles for the electrolytes have been focused on (1) various battery systems (LIBs with graphite or silicon anodes [34, 35], practical LMBs [36], lithium sulfur batteries [37], and anode-free lithium metal batteries [38]), (2) various solvent systems (fluorinated solvent [39], high concentration solvents [40], carbonate-based solvents [41], ether-based solvents [42], and ionic-liquid solvents [43]), and (3) special functions (flame retardant [44], film forming [45], and low temperature [46]). However, optimization principles, design strategies, and future perspectives for the electrolytes of the HVLMBs have not been summarized in detail.

**Figure 2:**
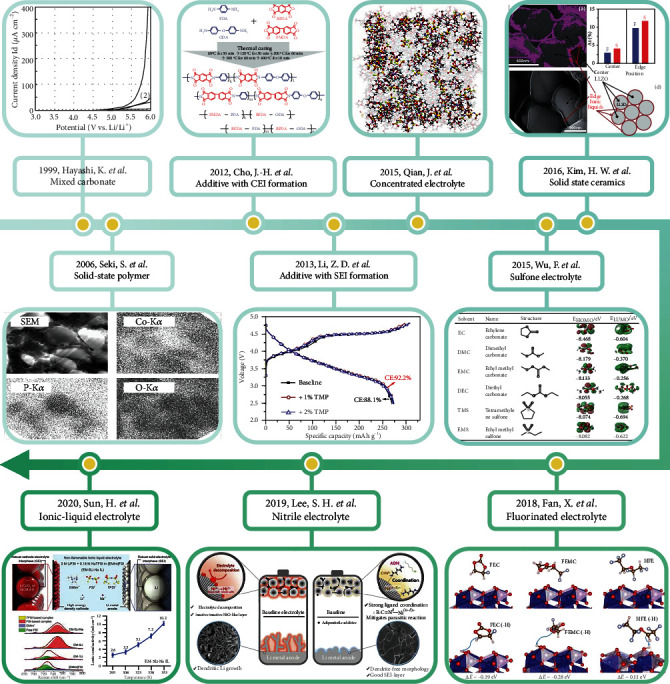
Brief timeline summarizing the historical development of electrolyte engineering for HVLMBs. 1999, mixed carbonate solvent for HVLMBs. Reproduced with permission [47]. Copyright 1999, Elsevier B.V. 2006, solid-state polymer for HVLMBs. Reproduced with permission [48]. Copyright 2006, Electrochemical Society, Inc. 2012, additive with CEI formation for HVLMBs. Reproduced with permission [49]. Copyright 2012, The Royal Society of Chemistry. 2013, additive with SEI formation for HVLMBs. Reproduced with permission [50]. Copyright 2013, Elsevier B.V. 2015, high concentration electrolyte for HVLMBs. Reproduced with permission [51]. Copyright 2015, Springer Nature. 2015, sulfone electrolyte for HVLMBs. Reproduced with permission [52]. Copyright 2015, American Chemical Society. 2016, solid-state ceramics for HVLMBs. Reproduced with permission [53]. Copyright 2016, The Royal Society of Chemistry. 2018, fluorinated electrolyte for HVLMBs. Reproduced with permission [54]. Copyright 2018, Springer Nature. 2019, nitrile electrolyte for HVLMBs. Reproduced with permission [55]. Copyright 2019, WILEY-VCH. 2020, ionic-liquid electrolyte for HVLMBs. Reproduced with permission [56]. Copyright 2020, WILEY-VCH.

**Figure 3:**
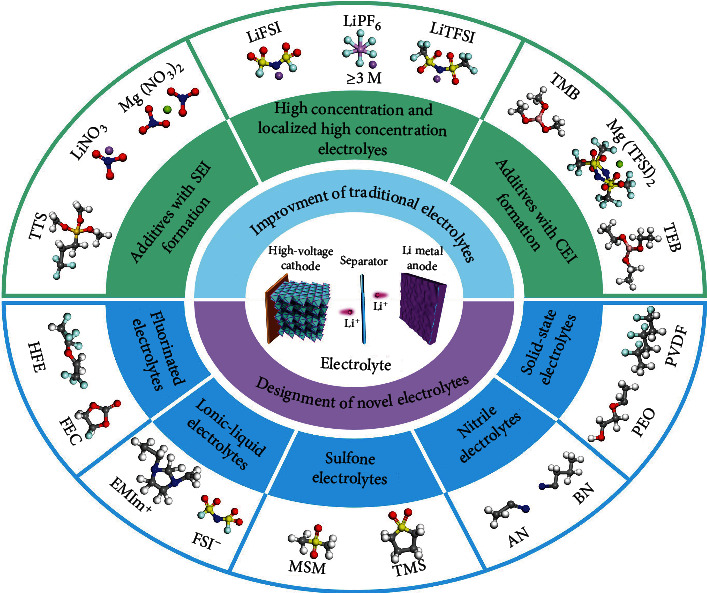
Schematic illustration of the frame structure of this review. Balls with various colors represent different atoms; color code: red: O; light green: P; blue: N; yellow: S; light blue: F; magenta: Li; grey: C; white: H; light red: B; green: Mg; brownish yellow: Si.

Herein, the improvement strategies of conventional electrolytes and the design of novel electrolytes have been proposed to enhance the stability and reliability of HVLMBs. A brief timeline summarizes the development of electrolyte engineering for HVLMBs (Figure 2). As shown in Figure 3, in this review, recent reports on the electrolytes for HVLMBs are reviewed in terms of the improvement of traditional electrolytes and design of novel electrolytes. The highest occupied molecular orbital (HOMO) and the lowest unoccupied molecular orbital (LUMO) values corresponding to the different substances in Figure 3 are displayed (Table 1). Theoretical studies and advanced characterization methods based on the electrolytes of the HVLMBs are also probed to study the internal mechanism for ultrahigh stability at an extreme potential. Furthermore, future hotspot directions and perspectives for further HVLMB electrolyte processing are also provided. Our review gives a multidimension perspective, which involves high-voltage cathode material science, Li metal anode chemistry, theoretical simulation, novel characterization, comprehensive overview of different solvents, comparison of various strategies, and perspectives of future directions.

## 2. Improvement of Traditional Electrolytes

### 2.1. High Concentration and Localized High Concentration Electrolytes

High concentration electrolytes (HCEs) exhibit unique ion solvation structures and anion-derived interfaces, which endows the electrolyte with a wide electrochemical window, excellent thermal stability, decreased flammability, and weakened current collector corrosion. The HCE design is mainly for solvent and lithium salt. For the solvent design, the carbonate-based, sulfone-based, and nitrile-based electrolyte solvents with strong reactivity and poor film-formation ability for Li metal are difficult to apply in HVLMBs. The formation of the anion-derived SEI layer induced in the HCEs can significantly enhance the compatibility between the electrolyte and Li anode. Moreover, the reduced free solvent number in HCE can improve the high-voltage stability of ether-based solvents, which promotes their applications in HVLMBs. In terms of lithium salt design, great success has been achieved on HCEs, primarily attributed to the LiF-rich SEI layer as derived from the anion. The big anion size in lithiumbis(fluorosulfonyl)imide (LiFSI) and lithiumbis(trifluoromethane sulfonyl) imide (LiTFSI) decreases the Coulombic forces between cations and anions in the electrolyte, ensuring high solubility in the solvents. In HCEs, the interaction between Li^+^ and anions significantly affects the interface formation and electrode passivation processes. Due to the uniform and dense LiF-rich EEIs as induced by TFSI^−^ and FSI^−^, the interface can remarkably inhibit Li dendrite growth and tolerate the significant volume change of high-voltage/capacity electrodes.

To achieve HCE effects while reducing the viscosity of the electrolyte, localized high concentration electrolytes (LHCEs) are developed. The construction difference between LHCEs and HCEs is attributed to the diluent introduction. The selection principles of the diluents mainly include (i) weakening Li^+^ binding energy without participating in the Li^+^ solvent shell to achieve localized high concentration environment, (ii) lowering the viscosity by reducing the HCE viscosity and increasing the wettability of the electrolyte for electrodes and separators, (iii) lowering the LUMO energy level and facilitating the SEI layer formation, (iv) lowering the cost for promoting large-scale applications, (v) lowering the combustibility and enhancing battery safety, and (vi) weakening toxicity and reducing pollution for battery recycling.

#### 2.1.1. High Concentration Electrolytes

**Figure 4:**
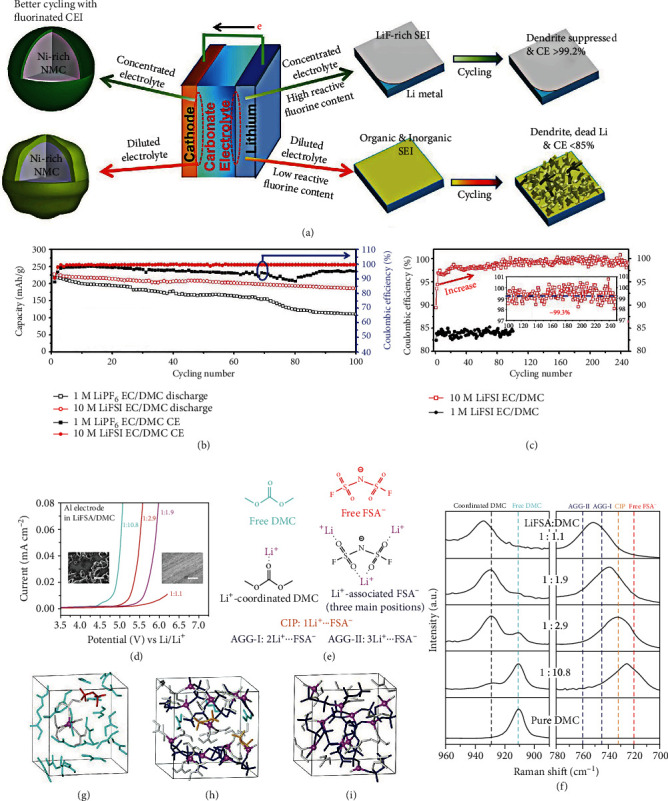
(a) Schematic illustration of the effect of concentrated and diluted electrolyte on a Li anode and Ni-rich cathode. (b) Cycling performance of NMC622||Li batteries with 1 M LiPF_6_ in EC/DMC and 10 M LiFSI in EC/DMC electrolytes in a 2.7-4.6 V voltage window. (c) CE of Li deposition/stripping in 1 M LiFSI in EC/DMC and 10 M LiFSI in EC/DMC electrolytes at a current of 0.2 mA/cm^2^. Reproduced with permission [57]. Copyright 2018, Elsevier B.V. (d) LSV curves of LiFSA/DMC electrolytes with various concentrations. The insets are SEM images of the Al surface polarized in 1 M LiFSI in EC/DMC and 10 M LiFSI in EC/DMC; the white scale bar represents 20 *μ*m. (e) Several main species in the LiFSA/DMC solvents. (f) Raman spectra of LiFSA/DMC solvents with various salt-to-solvent molar ratios. Snapshots of typical equilibrium trajectories obtained by DFT-MD simulations: (g) 1 LiFSA in 25 DMC, <1 mol dm^−3^, (h) 12 LiFSA in 24 DMC, ca. 4 mol dm^−3^, and (i) 10 LiFSA in 11 DMC, ca. 5.5 mol dm^−3^. Reproduced with permission [58]. Copyright 2016, Springer Nature.

In recent reports, high concentration electrolytes (HCEs) effectively enhance the Li metal stability and suppress the transition metal dissolution. By adding the LiFSI concentration to 10 M, Fan et al. [57] demonstrated outstanding cycling performance of the HVLMBs. As shown in Figure 4(a), the LiFSI salt is reduced to form the LiF-rich layer, which validly suppresses the electrolyte oxidation and impedes the Li dendrite growth. The superconcentrated electrolyte also stabilizes the high-voltage NMC cathode at an extreme voltage. As shown in Figure 4(b), the cycling stability of LiNi_0.6_Mn_0.2_Co_0.2_O_2_ (NMC622) ||Li batteries with 1 M LiPF_6_ in ethylene carbonate (EC)/dimethyl carbonate (DMC) and 10 M LiFSI in EC/DMC electrolytes is tested. The CE of the battery using traditional electrolyte owns up to 99% in the first few cycles. However, this CE steeply drops to only 95% after the first 20 cycles, and the specific capacity decays rapidly. After 100 cycles, the NMC622||Li battery using 1 M LiPF_6_ EC/DMC electrolyte achieves the capacity retention of ~52%, considerably lower than that in 10 M LiFSI in EC/DMC electrolyte (~86%), which significantly enhances the electrochemical performances of the HVLMBs compared with conventional electrolytes. The CEs of the Li/copper (Cu) batteries using the two different electrolytes are displayed in Figure 4(c). A high CE of ~97.5% is exhibited in the battery with the superconcentrated electrolyte, and the CE gradually increases to ~99.3% on the 80^th^ cycle. Density functional theory (DFT) calculations for LUMO energy values show that LiFSI owns a lower LUMO value (-1.70 eV) than those of DMC (-0.54 eV) and EC (-0.92 eV), which indicates that LiFSI is more inclined to react with lithium anode as compared with the solvents. In addition, the prioritized reduction of FSI anions with lithium anode is significantly increased as the molar ratio between salt and solvent increases from 0.105 in the diluted electrolyte to 1.05 in HCE. Without considering thermodynamics, the -SO_2_F groups of FSI anions also own a kinetical advantage over the carbonate solvents. Since the S-C bond is more stable than the C-F bond, the HCE with the LiFSA salt is expected to achieve better electrolyte suppression and transition metal dissolution effect as compared with that with LiFSI. Wang et al. [58] added an advanced lithium bis(fluorosulfonyl) amide (LiFSA) in traditional carbonate solvent to ultrahigh concentration. The superconcentrated electrolyte exhibits a dense network between anions and solvent molecules, and the network owns robust coordination with Li^+^ cations. As shown in Figure 4(d), the linear sweep voltammetry (LSV) confirms that a high concentration strategy can improve the oxidation potential of the electrolyte. The scanning electron microscopy (SEM) photographs of the polarized aluminum surface indicate the successful prevention of Al dissolution in such a superconcentrated electrolyte. The main substances in the LiFSA/DMC electrolytes are shown in Figure 4(e). As shown in Figure 4(f), an evident O-CH_3_ vibration (910 cm^−1^) is observed in free DMC. When Li^+^ is coordinated with the DMC molecule, the O-CH_3_ band rises to 930-935 cm^−1^. In the solvent of LiFSA : DMC = 1 : 10.8, most DMC molecules are independent as the molar ratio of solvent to salt is far higher than a conventional four- or five-time coordination amount of lithium ion. As the concentration of the LiFSA gradually increases, the free DMC molecule decreases and the coordinated 1,2-dimethoxyethane (DME) molecule with Li^+^ cations increases. Owing to the appearance of aggregate clusters (two or more Li^+^ cations coordinate with FSA^−^ anion) and contact ion pairs (only one Li^+^ cation coordinates with FSA^−^ anion), the formation of aggregate clusters is confirmed by a significant mobile of FSA^−^ cation band at 700-780 cm^−1^. Figures 4(g)–4(i) show the simulation snapshots of dilute electrolyte, moderately concentrated electrolyte, and superconcentrated electrolyte, respectively. For the moderately concentrated electrolyte, the intensity of the free DMC band significantly decreases in Raman spectroscopy, indicating that a large number of DMC molecules participate in the Li^+^ solvation. It is further confirmed by DFT calculations (Figure 4(h)), which display that ~90% of DMC molecules are involved in the Li^+^ solvation. As shown in Figure 4(i), for the superconcentrated electrolyte, Li^+^ cations coordinated with FSA^−^ anions form a robust three-dimensional network, effectively suppressing the dissolution of transition metal and anodic aluminum (Al) foil at a high voltage. In addition, there are two critical reasons for the improved stability of the electrolyte/cathode interface in HCE: (i) LiFSA exhibits lower reactivity to generate hydrofluoric acids than LiPF_6_, thus reducing the electrode corrosion. This effectively decreases the dissolution of the transition metal; (ii) even if little transition metal leakage occurs on the high-voltage cathode surface, they are difficult to dissolve and transfer through HCE with abundant ion aggregates.

#### 2.1.2. Localized High Concentration Electrolytes

**Figure 5:**
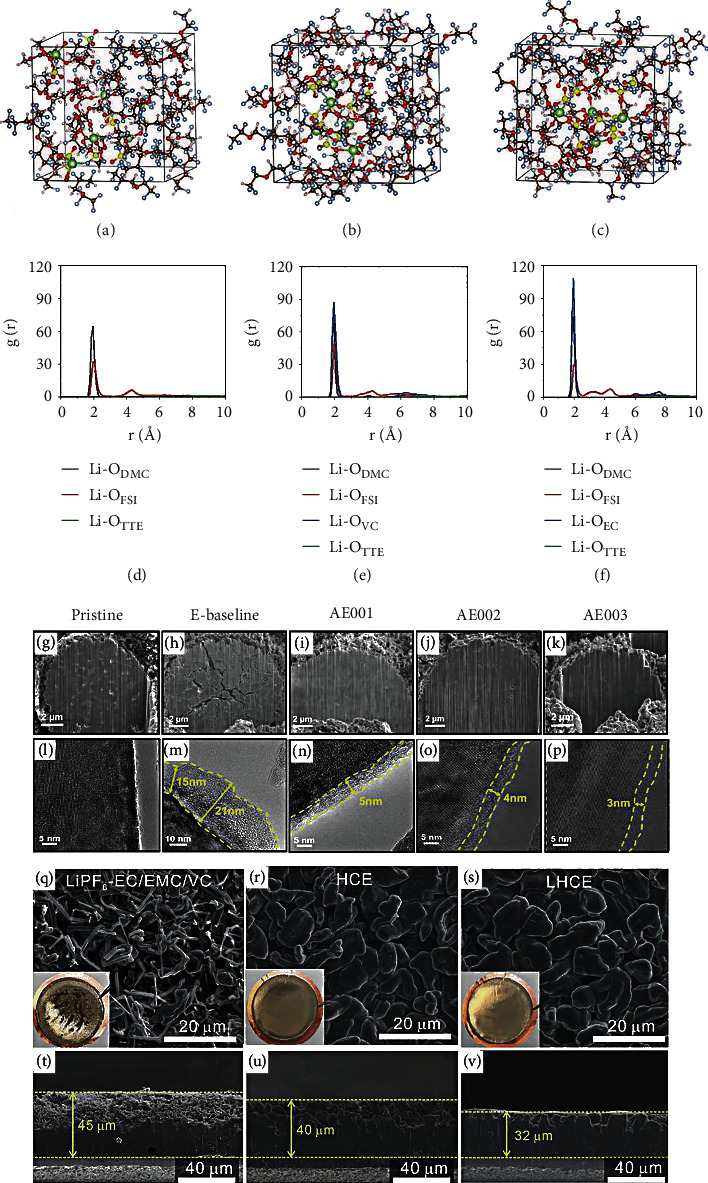
AIMD simulation snapshots of (a) LiFSI/DMC/TTE, (b) LiFSI/DMC/VC/TTE, and (c) LiFSI/DMC/EC/TTE. Color code: C: brown; O: red; Li: green; S: yellow; N: ice blue; H: pink. Radial distribution functions *g*(*r*) of Li-O_DMC_, Li-O_FSI_, Li-O_VC_, Li-O_EC_, and Li-O_TTE_ pairs in (d) LiFSI/DMC/TTE, (e) LiFSI/DMC/VC/TTE, and (f) LiFSI/DMC/EC/TTE. (g–k) Cross-sectional FIB/SEM images of NMC811 particles; (l–p) HRTEM images of the CEI layer morphologies on NMC811 cathodes. Reproduced with permission [59]. Copyright 2020, WILEY-VCH. (q–s) The top view and (t–v) cross-sectional view images of deposited Li films (0.5 and 4 mAh cm^−2^) in different electrolytes. Reproduced with permission [60]. Copyright 2019, Elsevier B.V.

By adding diluent into the HCEs, LHCEs are further developed, which enhances the wettability with the separator and electrode and reduces the use of Li salt to decrease the cost. Zhang et al. [59] enhanced the oxidation potential of LHCE to 4.9 V and achieved outstanding cycling performance of LiNi_0.8_Mn_0.1_Co_0.1_O_2_ (NMC811)||Li battery in 2.5-4.4 V. Figures 5(a)–5(c) show the simulation snapshots of LiFSI/DMC/1,1,2,2-tetrafluoroethyl-2,2,3,3-tetrafluoropropyl ether (TTE), LiFSI/DMC/vinylene carbonate (VC)/TTE, and LiFSI/DMC/EC/TTE electrolytes, respectively. The simulation results indicate that the LiFSI salt is associated with DMC and EC as well as VC molecules in diluent TTE solvent. The radial distribution function (*g*(*r*)) is conducted to further research the distances between different solvents and Li^+^ cations in the LHCEs. As shown in Figures 5(d)–5(f), strong peaks of the Li^+^-O_DMC_ are all ~1.95 Å, a bit smaller than Li^+^-O_FSI_^−^, demonstrating the excellent coordination between DMC molecules and Li^+^ cations. For the LHCEs with EC or VC solvents, a new peak of Li^+^-O_EC_/Li^+^-O_EC_ also appears at 1.95 Å, indicating that the EC and VC fully participate in the Li^+^ cation solvation. The stronger intensity of Li^+^-O_EC_/Li^+^-O_EC_ pairs than that of Li^+^-O_DMC_ pairs demonstrates the better coordination between Li^+^ cations and EC/VC molecules in the LHCEs. Likewise, the coordination between VC and Li^+^ is weaker than EC. In addition, TTE molecules have no association with Li^+^ cations in the three LHCE systems, suggesting that the TTE solvent as diluent shows no interaction with Li^+^ cation in the LHCE. To find out why the LHCEs in NMC811||Li batteries exhibit outstanding electrochemical performances, the NMC811 electrodes after cycling and cathode electrolyte interphase (CEI) on their surface are characterized by SEM and high-resolution transmission electron microscopy (HRTEM). The cross-sectional focused ion beam (FIB)/SEM images of the pristine and cycled cathodes are shown in Figures 5(g)–5(k). Many cracks appear on the NMC811 cathode after cycling in a traditional E-baseline electrolyte. In contrast, the electrodes after cycling in the three LHCE systems are well protected. As shown in Figure 5(i), a clean surface is observed at the fresh NMC811 cathode. A CEI layer with 15-21 nm thickness is unevenly distributed on the cycled cathode surface (Figure 5(m)). However, the NMC811 cathodes using the LHCEs exhibit more uniform and thinner CEI layers, specifically, ≈5 nm for LiFSI/DMC/TTE electrolyte (Figure 5(n)), ≈4 nm for LiFSI/DMC/VC/TTE electrolyte (Figure 5(o)), and ≈3 nm for LiFSI/DMC/EC/TTE electrolyte (Figure 5(p)). Despite the smaller thickness than the traditional electrolyte, the CEI layers in three LHCE systems exhibit better durability and mechanical strength, originating from the less transition metal dissolution than the traditional electrolyte. These results demonstrate that the advanced interfacial chemistry in the LHCEs efficiently protects the high-voltage cathode, further reducing the transition metal escape and electrolyte oxidation. In addition to the carbonate-based LHCEs, the ether-based LHCEs with an excellent affinity for lithium metal are also profoundly investigated. Ren et al. [60] developed an ether-based LHCE that can maintain the stability of NMC811 cathode under a high voltage of 4.5 V with the LiF-rich interface. Combined with the outstanding stability for Li metal in the LHCE, the NMC811||Li cells exhibit significantly enhanced cycling performance. In the LHCE system, FSI^−^ replaces the DME molecule in the Li^+^ solvation structure, and the addition of TTE diluent does not destroy the interaction between FSI^−^ and Li^+^. To research the Li dendrite growth behavior in the conventional electrolyte, HCE, and LHCE, the digital photos of the Li metals after cycling in Li||Cu batteries using various electrolytes are shown in the insets of Figures 5(q)–5(s). For the traditional electrolyte, an extremely uneven layer is observed, indicating a severe parasitic reaction at the anode-electrolyte interface. In contrast, flat Li layers are deposited in LHCE and HCE, suggesting uniform Li^+^ transmission in the two electrolytes. Large and smooth Li deposits are observed in SEM characterizations of these two electrolytes. However, apparent Li dendrite growth can be observed in the carbonate electrolyte. As shown in Figures 5(t)–5(v), for the LHCE, the cross-section picture of the anode exhibits a denser and uniform deposition layer with a thickness of 32 mm, considerably lower than those in the HCE (40 mm) and the carbonate electrolyte (45 mm). The Li deposit owning a larger contact area with carbonate electrolyte will induce a more severe parasitic reaction to cause a lower CE.

Except the TTE diluent, 1,2-difluorobenzene (1,2-dfben), fluorobenzene (FB), and bis(2,2,2-trifluoroethyl) ether (BTFE) with weak Li^+^ binding energy and strong fluorine-donating ability own huge potential as diluents. Yoo et al. [61] reported that 1,2-dfben could be used as a diluent solvent in the electrolyte to realize the LHCE effect. The low LUMO energy level and strong fluorine-donating ability of 1,2-dfben enhance the concentration influence at a relatively low lithium salt concentration of 2 M and achieve LiF-rich SEI composition. Jiang et al. [62] found that the introduction of FB diluent not only enhances the physical properties of the electrolyte but also modifies the Li^+^ solvation shell, which promotes the formation of LiF-rich electrode/electrolyte interfaces (EEIs). Beneficial from these improved features, the Li/LCO cell with the high-loading cathode (20.4 mg cm^−2^) using the LHCE shows stable cycling performances and high rate capacities at an extreme potential (4.6 V). Notably, the Li/LCO pouch battery delivers an energy density of 400 Wh kg^−1^ under harsh conditions (50 *μ*m Li, 2.7 g Ah^−1^ electrolyte amount). Chen et al. [63] reported a fire-retardant LHCE with the lithium salt of LiFSI and the nonflammable solvents of triethyl phosphate (TEP) and BTFE. The LHCE can sustain stable and dendrite-free cycling of HVLMBs with an average CE of 99.2%. Furthermore, it shows outstanding anodic stability up to 5 V and significantly enhances the electrochemical performances of HVLMBs.  Table 2 shows the cycling performance of the HVLMBs with different HCEs and LHCEs.

#### 2.1.3. Section Summary

In this section, recent development in the HCEs and LHCEs has been discussed, including carbonate-in-salt, ether-in-salt, and other organic solvent-in-salt electrolytes, such as 5.5 M LiFSA in DMC, 1.7 M LiFSI in DME/TTE, and 3.5 M LiTFSI in DMC/[C_2_mpyr][FSI]. Through increasing the salt concentration, the ion transfer mechanisms in HCEs and LHCEs are remarkably different as compared with traditional electrolytes. In conventional electrolytes, a big solvation structure is formed through coordination between Li^+^ and solvent, which seriously decreases Li^+^ mobility. However, the anion almost does not enter the solvation shell. Therefore, the conventional electrolytes own low Li^+^ transference numbers (<0.4), leading to abundant anions gathering on the cathode, generating severe concentration polarization and large overpotential [64]. On the contrary, for the distinctive solvation structure of the HCEs, Li cations can drag more anions into the solvation structure, which dramatically limits the anion mobility while less affecting the cation mobility, resulting in an extremely high Li^+^ transference number (>0.6) [65, 66]. This leads to a large mass transfer flux of Li cation, promoting the uniform and rapid substance exchange at the surfaces of high-voltage cathode and Li metal anode. It can also prevent the spatial charge layer induced by the anion consumption at EEIs. This decreases the electric field driving force and exacerbates the uneven deposition of Li^+^. In addition, the rival coordination of solvent and anion with Li cation controls the solubility of the lithium salt in the electrolyte and the generation of aggregates and ion pairs universally existing in the HCEs and maintaining the stability of the two electrodes in HVLMBs. Moreover, the reorganized Li^+^ solvation structure in the HCEs not only involves more anions but also reduces accessible free solvents, which enhances the electrochemical window of the electrolyte and optimizes the formation pathways of SEI and CEI layers to increase the mechanics and the electrochemical stability of EEIs. Overall, besides the widely received cancellation of free solvent at high concentrations, the contact ion pair and aggregate cluster formation facilitate the involvement of anions in the passivation layer formation and thus essentially enhance the inorganic substances in the SEI/CEI layers and increase the electrolyte oxidation resistance. The distinctive solvation structure and the anion-derived interface endow the electrolyte with surprising properties, such as enhanced oxidation potential, improved thermal stability, decreased flammability, and intensive compatibility with the current collector. Therefore, the electrochemical performances of HVLMBs can be significantly enhanced using HCEs and LHCEs.

### 2.2. Additives with CEI Formation

The high voltage leads to continuous decomposition of conventional carbonate-based electrolytes at cathode surfaces, often generating an unstable, nonuniform, and nonprotective CEI layer, impeding the Li^+^ migration and decreasing the electrochemical efficiency. Various side reactions, such as irreversible structural change, leakage of transition metals, and release of lattice oxygen, also emerge and considerably deteriorate battery performances. Compared with other strategies of enhancing the electrochemical performances of HVLMBs, the strategy of using CEI additives owns less dosage, simple method, low cost, and prominent effect. Thus, it has attracted widespread concerns. The HOMO energy value of most CEI additives is lower than that of solvents, so they can preferentially participate in the electrochemical reactions on the electrolyte/cathode interface, forming a robust and uniform film on the cathode surface to suppress the decomposition of electrolytes and decrease the effect of various side reactions. The higher voltage pursuing in the high-energy-density Li batteries has exceeded the anodic limits of the commercial electrolytes, therefore adding CEI additive to form a protective layer that blocks the electron transport and meanwhile enables efficient Li^+^ access is one of the most effective strategies.

**Figure 6:**
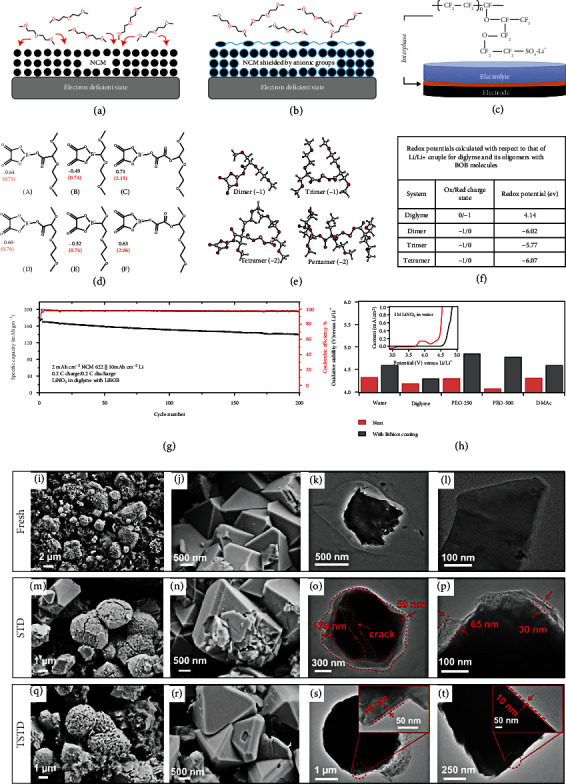
(a, b) A pictorial illustration of the mechanism by which oxidation of ethers is inhibited at a high-voltage CEI containing a layer of immobilized anions. (c) Schematic illustration of the structure of lithiated Nafion^™^ (Lithion) utilized to form the CEI. (d) Structures of plausible coupling products of BOB^2-^ and diglyme. Calculated reaction-free energies for forming anionic (green) and neutral (red color) dimers. (e) Optimized geometries and respective charges for the dimer and higher-order coupling products of BOB and diglyme. (f) Table of calculated redox potentials for diglyme and its oligomers with BOB molecules. (g) Cycling performance for a Li||NCM cell with diglyme–LiNO_3_–HfiP-lithium bis(oxalate) (LiBOB) electrolyte. (h) Bar chart comparing the oxidative stability of different electrolytes with (black) and without (red) lithion coating. Reproduced with permission [78]. Copyright 2019, Springer Nature. SEM (left) and TEM (right) images of the LNMO cathodes under the conditions of (i–l) pristine LNMO cathode and after 200 cycles in (m–p) STD and (q–t) TSTD electrolytes. Reproduced with permission [79]. Copyright 2019, The Royal Society of Chemistry.

By forming a stable and even CEI layer, the additives alleviate the continuous degradation of electrolytes and inhibit the transition metal dissolution, enhancing the cycling stability of HVLMBs. Choudhury et al. [78] demonstrated that the CEI composed of preformed supramolecules and anionic polymers achieves an advanced strategy for enhancing the stability of ether electrolytes at an extreme voltage. As shown in Figures 6(a) and 6(b), the charged glyme-bis (oxalate)borate (BOB) oligomers, which are incredibly stable at a high voltage, form a dense CEI by robust noncovalent interactions. The oligomer CEI effectively reduces the side reaction between the electrolyte and the high-voltage cathode. As shown in Figure 6(c), the semicrystalline polymer with the following three advantages is studied. First, the solutions of Li^+^ cations in aprotic alcohol and carbonate ester exhibit low viscosity for the successful transportation of the polymer to the pores of the prefabricated cathode through the liquid carrier. Second, the Li^+^ coated interfaces are previously studied to indicate that the effective electrostatic shielding is formed by the negative charge centers generated by the dissociation of the sulfonate groups. The shield inhibits the migration of negatively charged substances on the surface of the electrode without compromising the transport of cations. It induces high interfacial ionic conductivities and Li^+^ transference numbers. Finally, the hydrophobic and hydrophilic domains coexist in the Li^+^ cation separator, which means that the strongly polarized molecules will be slowed down in solvent. Figure 6(d) displays the only coupling product and the corresponding free energy change. The simulations show that the negatively charged substances are thermodynamically prone to be formed compared with their neutral analogs. Thermodynamically, the C-C produced by the CO_2_ release is preferable (Δ*G* = −0.64 eV). To evaluate the generate possibility of supramolecules, oligomers, or polymers, the reaction energies for these species are calculated (Figure 6(e)). These results demonstrate that the generation of negative charge and neutral trimer is thermodynamically difficult, along with the higher-order polymers. The anion and neutral form of the trimer formed by the dimer are endothermic peaks of 1-4 eV, and the formation of higher-order coupling products is very difficult owing to the high Δ*G*. As the potentials increase, the trimers may appear, but further polymerization seems impossible. Higher-order oligomers with multiple charges are unstable owing to the easy dissociation into lower-order charged dimers or trimers. Based on this situation, Figure 6(f) displays the calculations of the redox potentials of the diglyceride molecule, as well as the oligomer with the BOB molecule. In particular, the measured and computationally predicted infrared (IR) spectra all confirm that the oligomer is stable at an extreme voltage. The LSV in a 3-electrode setup and the more rigorous electrochemical floating-point examination show the enhanced oxidation potential with the existence of the oligomer. The cyclability of Li||NCM cell utilizing the diglyme-LiNO_3_-tris (hexafluoro-iso-propyl)phosphate (HFiP) electrolyte is studied, as displayed in Figure 6(g). The battery exhibits a CE of up to 98%, and the capacity decays only 20% after 200 cycles at 0.2 C. As shown in Figure 6(h), the improvement of oxidation potential is observed in several electrolyte systems. The enhanced oxidation of anionic Li^+^ cation coating is universal. The introduction of F on the basis of B-containing additives can further enhance the stability of the electrolyte/cathode interface. Yue et al. [79] demonstrated that tris(pentafluorophenyl) borane (TPFPB) additive with CEI formation stabilizes the LNMO cathode at elevated potentials and the Li anode. SEM is conducted to study the CEI generation on the LNMO cathode. The HOMO value of TPFPB (-7.89 eV) is higher than those of EMC (-8.16 eV) and EC (-8.44 eV). The LUMO energy level of TPFPB is -3.54 eV, considerably lower than those of EMC (0.82 eV) and EC (0.54 eV). Such a large energy landscape ensures the early redox reaction of TPFPB over the carbonate solvents, promoting the stable EEI formation on the electrodes and inhibiting the decomposition of EC and DMC. For the pristine LNMO electrode, octahedral crystal shape and spherical secondary particles are observed (Figures 6(i) and 6(j)). The LNMO cathode after 200 cycles in the standard electrolyte (STD) is damaged (Figures 6(m) and 6(n)). On the contrary, both types of cathodes in the STD with 1 wt.% TPFPB (TSTD) are relatively intact after 200 cycles (Figures 6(q) and 6(r)). The transmission electron microscope (TEM) characterizations are used to further evaluate the CEI in various electrolytes. For the fresh LNMO cathode, no CEI layer is observed (Figures 6(k) and 6(l)). As shown in Figures 6(o) and 6(p), the nonuniform CEI layer with a thickness ranging from 59 to 178 nm is formed on the surface of LNMO particles in the STD electrolyte. It is because of the electrolyte decomposition on the cathode. By-products of the decomposition are electronically insulating and can cause these LNMO particles to inactivate [80]. Therefore, the impedance continues to increase to cause difficulties in delithiation and lithiation. As shown in Figures 6(s) and 6(t), a thin and uniform CEI ~10 nm is generated in the TSTD electrolyte. This thin CEI prevents side reactions between the electrolyte and the LNMO cathode and ensures fast Li^+^ transmission.

**Figure 7:**
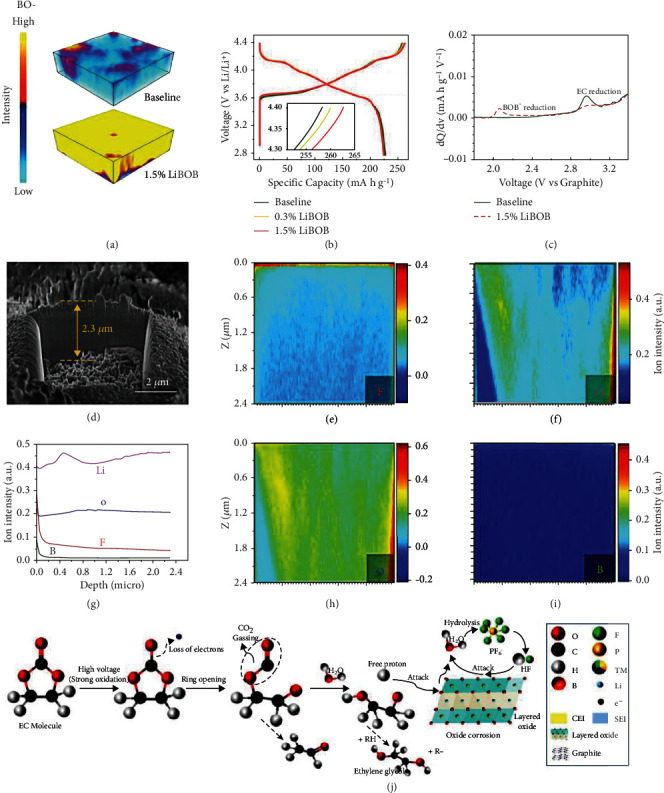
(a) 3D distribution of LiBOB decomposition species (represented by BO^−^) in the TOF-SIMS sputtered volume on the LiNi_0.94_Co_0.06_O_2_ cathode surface retrieved from 500-cycle full cells with baseline electrolyte and 1.5% LiBOB. (b) The initial charge-discharge curves of Li||LiNi_0.94_Co_0.06_O_2_ cells with different electrolytes. (c) *dQ*/*dV* curves of the cells with baseline electrolyte and 1.5% LiBOB. Reproduced with permission [81]. Copyright 2020, WILEY-VCH. (d) Surface morphology of LNO particle after 100 cycles in F-262A electrolyte for TOF-SIMS. (e, f, h, and i) The depth profiles of TOF-SIMS analysis for F, Li, O, and B elements on the cycled active particles. (g) The corresponding Li, O, F, and B element distribution in the sputtered active particle. Reproduced with permission [82]. Copyright 2019, Elsevier B. (j) Schematics of carbonate-based electrolyte decomposition in a high-voltage layered oxide system using EC as an example. Reproduced with permission [83]. Copyright 2022, WILEY-VCH.

Li et al. [81] manipulated the electrode/electrolyte interphase with a LiBOB additive to enhance the cycle life of LiNi_0.94_Co_0.06_O_2_||Li cell at an extreme potential.  Figure 7(a) shows a 3D view of the distribution of BO-segments in the time of flight secondary ion mass spectrometry (TOF-SIMS) sputtering volume collected near the surface of the cycled cathode with or without LiBOB. The BO^−^ signal on the LiNi_0.94_Co_0.06_O_2_ cathode in 1.5% LiBOB electrolyte is more evident and uniform than the baseline electrolyte. Therefore, LiBOB salt initially oxidizes and changes the original CEI composition.  Figure 7(b) displays the charge-discharge curves of various electrolytes at the initial cycle for the LiNi_0.94_Co_0.06_O_2_||Li batteries at 0.1 C. The discharge and charge capacities of the cell with the baseline electrolyte are 228 and 258 mAh g^−1^, corresponding to 88.2% CE. The charge capacities of the cells with the 0.3% LiBOB and 1.5% LiBOB electrolytes are 260 and 263 mAh g^−1^, respectively, with CE of 86.8% and 85.6%. The initial oxidation of BOB^−^ anions causes lower CE. Besides, the corresponding differential capacity (*dQ*/*dV*) curve offers a more direct and detailed comparison (Figure 7(c)). The battery using baseline electrolyte exhibits an apparent peak at 3.0 V, ascribed to the decrease of EC when the electrolyte-anode interphase is formed on the anode surface. Overall, this study demonstrates that B_*x*_O_*y*_ groups are introduced in the EEIs by the sacrificial decomposition of LiBOB, which enhances the interfacial Li^+^ diffusivity and gives rise to superior electrochemical stability of the high-voltage cathode. Compared with LiNi_0.94_Co_0.06_O_2_, the stable CEI construction of Co-free LiNiO_2_ is more difficult due to the continuous Ni dissolution, structural disordering, and particle cracking. Deng et al. [82] developed a fluorinate-rich electrolyte with lithium difluoro(oxalate)borate (LiDFOB) salt to generate a strong fluoride- (F-) and boron- (B-) rich CEI layer. The capacity retention of LiNiO_2_||Li cell reaches as high as 80% after 400 cycles under an extreme potential of 4.4 V. The cycled LiNiO_2_ cathodes in 1 M LiPF_6_ in EC/DMC = 1/1, *v*/*v* and 1 M LiPF_6_ + 2 wt.% LiDFOB dissolved in FEC/FEMC/HFE = 2/6/2, *w*/*w*/*w* (F-262A) electrolyte are tested by TOF-SIMS to study the surface compositions (Figures 7(d)–7(i)). As shown in Figure 7(d), the Ga^+^ ions sputter the crater to form the edge surface. Plentiful F and B signals are generated on the LiNiO_2_ cycled in the F-262A electrolyte. The ion intensity of Li and O elements remains unchanged from the depth profile (Figure 7(g)), suggesting a strong and thin CEI layer with high-ratio F and B elements on the cycled LiNiO_2_ with the F-262A electrolyte. With the F-262A electrolyte, the LiNiO_2_ cathode exhibits a superior capacity of 216 mAh g^−1^ with lower capacity decay of <20% after 400 cycles at 0.5 C. The significantly improved cycling performance originates from the following two aspects: (i) the CEI with high-ratio F and B elements effectively inhibits the continuous side effects on the LiNiO_2_ cathode, and (ii) the SEI with high-ratio F and B elements suppresses the parasitic reaction for the preservation of the Li anode.  Figure 7(j) shows that a small amount of HF corrosion helps form a uniform CEI layer.  Table 3 shows the cycling performance of the HVLMBs with CEI formation using different additives.

#### 2.2.1. Section Summary

In this section, recent development in the CEI additives has been discussed, including boron additive, phosphorus additive, nitrile additive, fluorine additive, unsaturated carbonate derivatives, and silane additives, such as HFiP, TPFPB, LiBOB, LiDFOB, and TMSPO. Electrolyte additive for CEI formation provides a practical and facile method to enhance the electrochemical performances of HVLMBs by *in situ* managing the chemical/physical properties/structures of CEI. The ideal CEI structure must be compact, continuous, uniform, and thin, accelerating Li^+^ conductivity, suppressing the leakage of transition metals and lattice oxygen, and separating high-voltage cathode and electrolyte to suppress the side reactions. Also, the CEI should balance flexibility and mechanical strength to tolerate the morphology changes of cathodes. Furthermore, it should be electrochemically stable in the extreme voltage environment. The abovementioned properties and component element types of the film generated on the cathode surface are variant, and the reaction mechanism is different. Therefore, it is worth exploring their internal mechanism of action and integrating various functions into one additive.

### 2.3. Additives with SEI Formation

Apart from regulating Li salts and solvents, improving the electrolyte with SEI additives is another efficient strategy to stabilize the SEI layer and thus inhibits the growth of Li dendrite in HVLMBs. For the additives for SEI improvement, electrolyte additives are developed to be sacrificial to facilitate stable SEI formation during the initial activation cycles, retaining the SEI stability and inhibiting the electrolyte decomposition in the subsequent cycling of HVLMBs. Therefore, the effective SEI additive should participate in the reactions with Li anode before electrolyte solvents, forming an extremely stable film with large Li^+^ conductivity to ensure the interfacial stability in HVLMBs in the repeated charging/discharging process.

**Figure 8:**
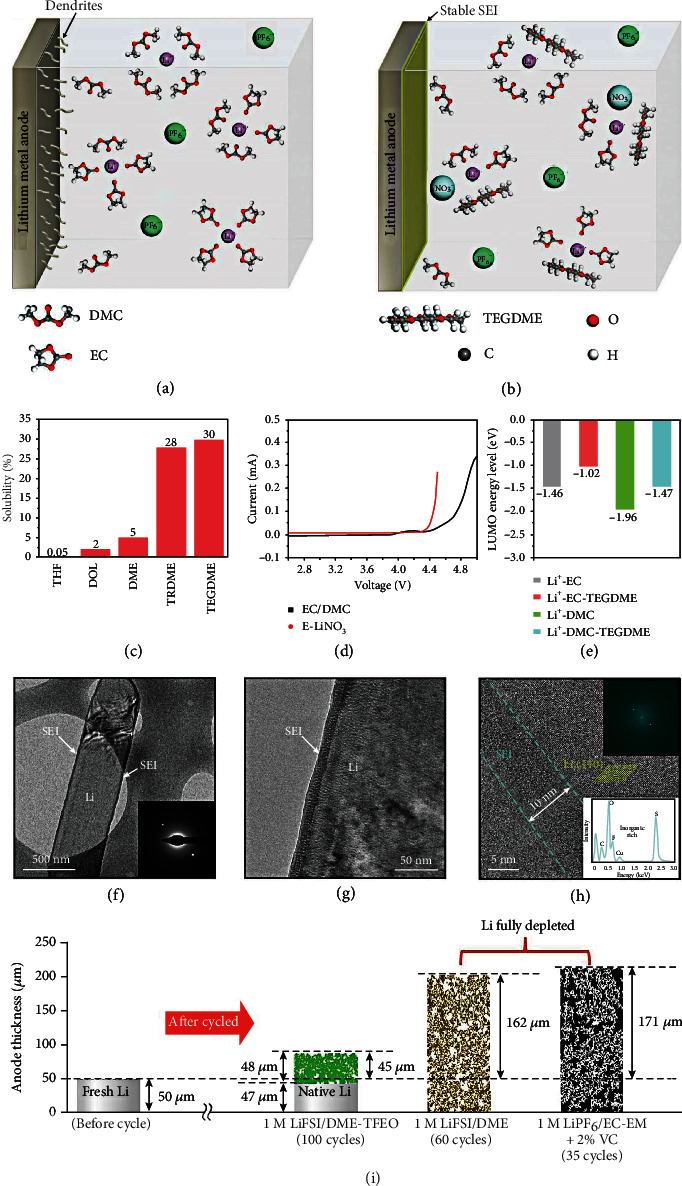
Illustration of (a) the unstable SEI with dendrite growth behavior in the EC/DMC electrolyte and (b) the stable SEI with uniform Li plating behavior in the E-LiNO_3_ electrolyte. (c) The solubility of LiNO_3_ in various ether solvents. (d) LSV curves of EC/DMC and E-LiNO_3_ electrolytes. (e) LUMO energy levels of Li^+^-EC, Li^+^-EC-TEGDME, Li^+^-DMC, and Li^+^-EC-TEGDME. Reproduced with permission [96]. Copyright 2020, WILEY-VCH. (f–h) Cryo-EM images of Li deposited on a TEM grid at different scales. Inset in (f): corresponding selected-area electron diffraction pattern. Inset in (h): corresponding reduced fast Fourier transform (top) and energy dispersive spectroscopy spectra (bottom) of the SEI layer. The yellow lines show the lattice space of the crystalline Li. (i) Schematic of Li loss and corresponding thickness (volumetric) expansion after different cycles in Li||NMC811 using 1 M LiFSI/DME-TFTO, 1 M LiPF_6_/EC‐EMC + 2%VC, and 1 M LiFSI/DME electrolytes. Reproduced with permission [97]. Copyright 2019, Springer Nature.

Uneven Li^+^ deposition and severe parasitic reaction hinder the development of the HVLMBs with high energy density. Developing additives to stabilize the SEI layer is essential for the issues above. A mechanically robust, chemically inert interface can avoid the continuous reaction between electrolyte and lithium metal, inhibiting capacity loss and CE attenuation [95]. Xiao et al. [96] developed a tetraglyme (TEGDME) additive to adjust Li^+^ solvation for a robust SEI layer while maintaining a voltage window suitable for the high-voltage cathodes. As shown in Figures 8(a) and 8(b), the TEGDME participates in the Li^+^ solvation in ester electrolyte for the SEI generation. The TEFDME solvent remarkably enhances the LUMO energy levels of Li^+^ solvation to prevent electrolyte decomposition. As shown in Figures 8(c)–8(e), to choose the best cosolvent for LiNO_3_ dissolved in the ester-based electrolyte, several cyclic and linear ethers are studied. The TEGDME solvent shows a 30 wt.% solubility of LiNO_3_ salt, considerably higher than other cyclic and linear ethers (Figure 8(c)). As shown in Figure 8(d), the electrochemical windows of 1 M LiPF_6_ in EC/DMC = 1/1, *v*/*v* (EC+DMC) and 1 M LiPF_6_ in EC/DMC/TEGDME = 2.4/5.6/2, *w*/*w*/*w* (E-LiNO_3_) electrolytes are studied by LSV. The traditional carbonate electrolyte shows an oxidational potential of 4.4 V, and the E-LiNO_3_ electrolyte is stable at 4.3 V. Besides, the TEGDME solvent remarkably enhances LUMO energy levels of both the Li^+^-DMC and Li^+^-EC, suggesting a reduced electrolyte decomposition and enhanced reduction stability (Figure 8(e)). The partial H atoms in ether additives are replaced with F atoms, which can effectively promote the LiF-rich SEI layer on the lithium anode surface. Cao et al. [97] reported an advanced electrolyte with tris(2,2,2-trifluoroethyl)orthoformate (TFEO) solvent alleviating the Li depletion and pulverization. TFTO owns a high boiling point of 145°C, which is beneficial for improving the safety and working temperature of the battery. In addition, TFEO does not own unstable organic groups (such as carbonyl, sulfonic, and cyano groups) that are strongly reactive with the lithium metal anode but only owns lithium-friendly ether groups. TFSO also contains the CF_3_ groups with powerful electron-withdrawing effects, which widen the electrochemical window. The SEI layer generated in this electrolyte shows overall characteristics, which can cause uniform Li^+^ stripping/plating and slow depletion of electrolyte and Li anode. Figures 8(f)–8(h) display the Li metal anodes after the deposition in the TFEO electrolyte tested by high-resolution cryoelectron microscopy (cryo-EM). As illustrated in Figures 8(f) and 8(g), a uniform and thin SEI layer is observed on the deposited Li metal cycled in the TFEO electrolyte. We can clearly observe the composition of the SEI layer and Li deposition direction when the magnification of cryo-EM is raised to the atomic level. As shown in Figure 8(h), the SEI layer ~10 nm is shown in the dashed line. Moreover, energy dispersive spectroscopy (EDS) inserted in Figure 8(h) shows that the SEI layer generated in the TFEO electrolyte mainly consists of inorganic substances, which are high-ratio S-, O-, and F-containing compounds from LiTFSI decomposition. More importantly, these inorganic substances are amorphous instead of crystallized, which is proved by electron diffraction inserted in Figure 8(f) and the simplified fast Fourier transform inserted in Figure 8(h). This structure is entirely different from the traditional perception of the SEI structure. For the multilayer- and mosaic-type structures for the SEI layer, enriched inorganics are highly crystallized at the SEI layer. Therefore, these SEI layers are usually nonuniform. In contrast, a highly amorphous structure is shown in the SEI layer using the TFEO electrolyte even if it has a high-ratio inorganic species. As shown in Figure 8(i), a pictorial illustration showing the expansion and depletion of Li metal after cycling is provided. In 1 M LiFSI/DME-TFEO electrolyte, the inhibition of Li depletion and volumetric expansion reveals enormous potential for the practical application of the HVLMBs.

**Figure 9:**
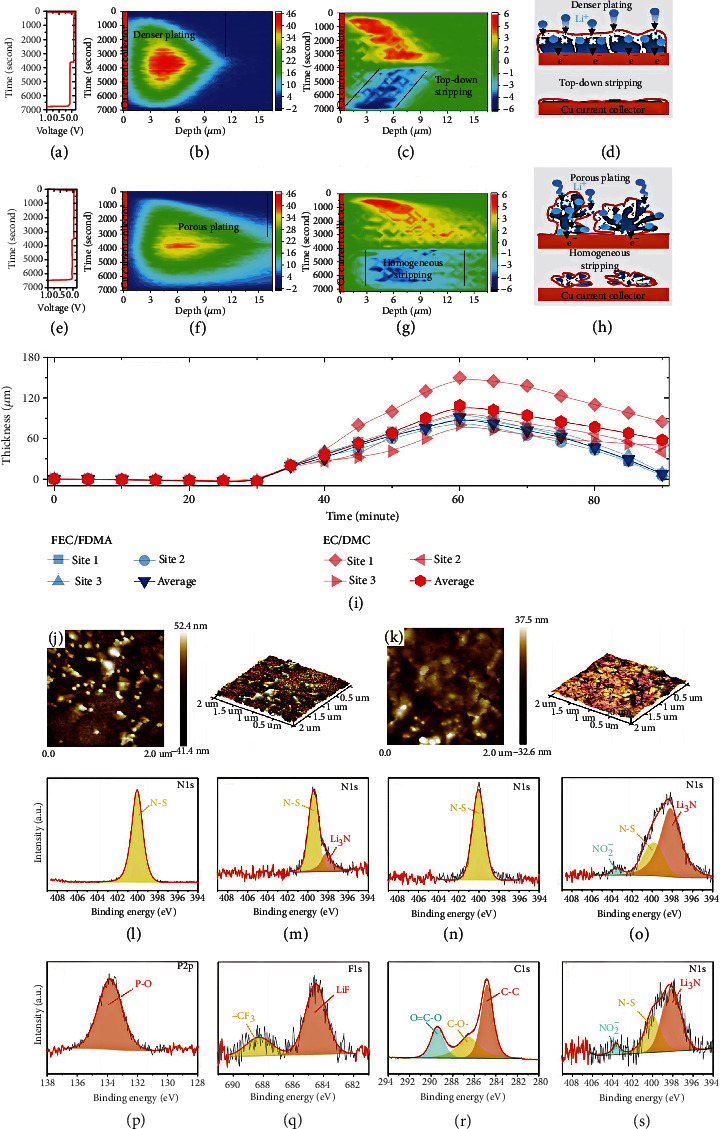
Depth profiles during Li metal plating/stripping on a Cu working electrode cycled at a current density of 1 mA cm^−2^ to a capacity of 1 mAh cm^−2^ of (a) 1 M LiTFSI in FEC/FDMA and (e) 1 M LiPF_6_ in EC/DMC electrolytes. Evolution of Li^+^ plating/stripping density *vs.* time from *operando* NDP during the first cycle at 1 mAh cm^−2^ in Li||Cu pouch cells for (b) 1 M LiTFSI in FEC/FDMA and (f) 1 M LiPF_6_ in EC/DMC electrolytes. Evolution of Li^+^ plating/stripping activity for (c) 1 M LiTFSI in FEC/FDMA and (g) 1 M LiPF_6_ in EC/DMC electrolytes, which is obtained from the change in Li^+^ density upon each time step of (b, f), respectively. (d, h) Schematic representation of the plating/stripping mechanism in the two electrolytes. (i) The evolution of thickness for the deposition during Li plating/stripping. Reproduced with permission [98]. Copyright 2020, Springer Nature. 2D and 3D Atomic Force Microscope (AFM) images of Li surface in (j) OPEs and (k) MOPEs. N 1s X-ray photoelectron spectroscopy (XPS) spectra of Li anode after immersion in (l) OPEs and (m) MOPEs for 4 h. (n) N 1s XPS spectra for OPEs and (o) N 1s, (p) P 2p, (q) F 1s, and (r) C 1s spectra for MOPEs after formation process. (s) N 1s spectra for MOPEs after 150 cycles. Reproduced with permission [99]. Copyright 2019, WILEY-VCH.

Wang et al. [98] developed advanced amide-based electrolyte-induced interface products. The time-dependent change of Li^+^ plating/stripping density is studied from the operating neutron depth profile (NDP) in the first cycle of the Li||Cu cell using fluoroethylene carbonate (FEC) + 2, 2, 2‐trifluoro‐N, N‐dimethylacetamide (FDMA) (Figures 9(a) and 9(b)) and EC+DMC (Figures 9(e) and 9(f)) electrolyte. FDMA owns the lowest LUMO energy value among all electrolyte solvents, indicating excellent electron affinity, which can be preferentially decomposed and assist the interface formation. In addition, the FDMA as an N-containing component generates LiNO_3_ in the interface, which is widely considered to be beneficial in forming a uniform and dense SEI layer. As shown in Figure 9(b), a thinner and denser Li layer is deposited in the FEC+FDMA electrolyte. On the contrary, a thicker and looser Li layer is observed in the carbonate electrolyte. It is worth noting that apparent asymmetry occurs in plating and stripping. The time derivative of the Li density verifies such phenomenon more visibly (Figure 9(g)), demonstrating the deep research of the plating/stripping behavior. The stripping of Li is evenly distributed throughout the deposited thickness with the carbonate electrolyte, while compared to plating, the stripping activity in the FEC+FDMA electrolyte moves back to the current collector symmetrically. Uniform stripping contributes to a morphology full of pores, accelerating the generation of high contact regions and lithium metal domains falling apart from the current collector, thus forming a dead Li layer. Conversely, as shown by the FEC+FDMA electrolyte in Figure 9(c), stripping from the top indicates a highly reversible plating/stripping mechanism. As shown in Figures 9(d) and 9(h), the top-down plating/stripping mechanism is the origin of high reversibility. As displayed in Figure 9(i), the thickness variation of Li anode plating/stripping with two different electrolytes proves the more uniform and denser deposition in the FEC+FDMA electrolyte. In addition to amide-based additives, LiNO_3_ can also inhibit the production of dead Li. Tan et al. [99] reported a nitriding interface by adding LiNO_3_ in organic phosphate electrolytes (MOPFs) to enhance combability between Li anode and organic phosphate electrolytes (OPEs). As displayed in Figure 9(j), the Li metal surface in OPEs is very rough with scattered particles of different sizes. In contrast, a uniform layer comprises an approximately flat quasicircular structure with a 400-500 nm diameter in MOPEs (Figure 9(k)). The uniform SEI without dead Li is essential for fast and uniform Li deposition. As displayed in Figures 9(l)–9(s), the SEI components in different electrolytes are studied. The Li_3_N compounds (398.5 eV) are generated on the interface using MOPEs (Figures 9(l) and 9(m)). For the Li metal with OPE, the N-S band is only found in the N 1s spectrum (Figure 9(n)) and may be derived from LiNO_3_ salt. As shown in Figure 9(o), the amount of Li_3_N enhances, and LiN_*x*_O_*y*_ emerges in MOPEs. In MOPEs, the SEI consists of Li_3_N, LiF, ROCOOLi, ROLi, and Li_*x*_PO_*y*_ compounds (Figures 9(q) and 9(r)). Notably, the Li_3_N owns a Li^+^ conductivity of up to 10^−4^ S cm^−1^, which effectively inhibits the dead Li formation and reduces the interfacial resistance. After 150 cycles, the SEI components in MOPE remain stable (Figure 9(s)). Summarily, the nitriding interface is successfully formed in MOPE, and the robust SEI layer with high-ratio Li_3_N compound is more conductive, which accelerates the ion transmission of the interface. It ensures stable cycling performance and uniform plating/stripping.  Table 4 shows the cycling performances of the HVLMBs with SEI formation using different additives.

#### 2.3.1. Section Summary

In this section, recent development in the SEI additives has been discussed, including boron additive, phosphorus additive, nitrile additive, and fluorine additive, such as HFiP, TEGDME, TFEO, FDMA, LiNO_3_, CTAC, and TTS. The SEI additives are of great importance in maintaining the stability of the electrolyte/anode interface and enhancing the cycling performances of HVLMBs. Nevertheless, owing to the small dose of additives and the intricate electrochemical reaction process in the HVLMBs, it is difficult to construct an interfacial layer with homogeneous and controllable composition and structure by introducing additives into electrolytes. Therefore, integrating the SEI additives with other modification strategies, such as CEI additives, HCEs, LHCEs, and novel solvents, can realize improved effects on stabilizing Li metal anode and high-voltage cathode.

## 3. Design of Novel Electrolytes

### 3.1. Fluorinated Electrolytes

Fluorinated compounds have received considerable attention in the electrolytes for lithium-based batteries due to their desirable features, including excellent antioxidation stability, low flammability, low melting point, and enhanced wettability for electrode and separator, along with the easy generation of a robust and uniform interfacial layer between electrolyte and electrode. Furthermore, partially fluorinated organic solvents own a stronger polarity than their perfluorinated counterparts and are miscible with other polar electrolyte solvents. Therefore, fluorinated solvents exhibit completely different physical features as compared with their nonfluorinated analogs and are suitable candidates for high-voltage applications. In terms of the fluorinated electrolyte design, the position of the fluorinated group in the molecular structure has a profound effect on the capabilities of LiF formation and Li^+^ coordination. Moreover, the combination of excellent oxidative stability and high ionic conductivity of the electrolyte is also significant for the fluorinated electrolyte design.

**Figure 10:**
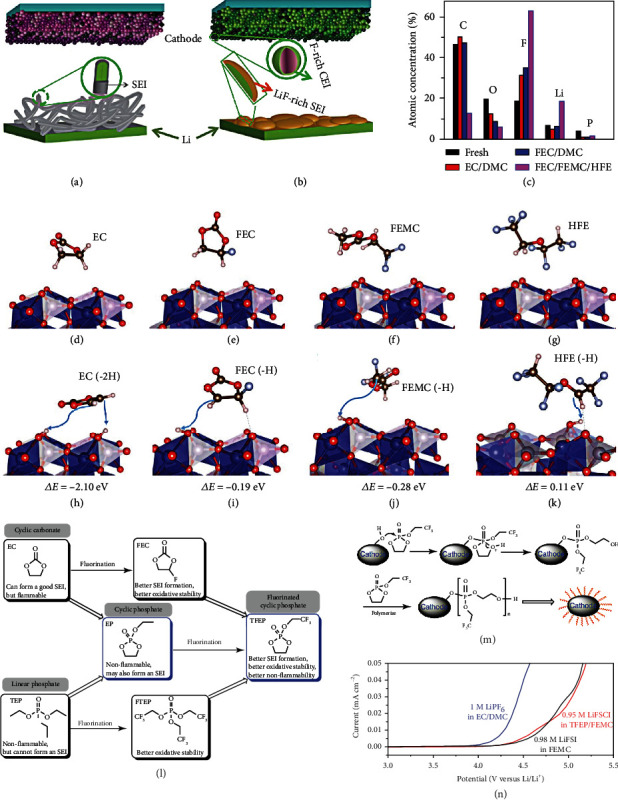
Variation of SEI and CEI chemistries formed in (a) traditional carbonate and (b) all-fluorinated electrolytes. (c) Composition of CEI on the cycled LCP cathodes in different electrolytes. (d–g) Initial and (h–k) final configurations and reaction energies. O: red; C: brown; F: light blue; H: white. Reproduced with permission [54]. Copyright 2018, Springer Nature. (l) Design of the fluorinated cyclic phosphate solvent. (m) Schematic illustrations of the ring-opening polymerization of TFEP for the formation of CEI. (n) Oxidative stabilities of the different electrolytes. Reproduced with permission [21]. Copyright 2020, Springer Nature.

By introducing fluorine (F) into the electrolyte structure, remarkable progress in battery chemistry can be achieved. Fan et al. [54] developed a fluorinated electrolyte that realizes the stable function of high-voltage cathodes in LIBs. The solvent composition leads to a huge difference in stability between the different electrolytes on the surfaces of high-voltage cathodes and Li anode. The F content in these electrolytes is the most apparent difference, 0 M, 1.8 M, and 22 M, for EC+DMC, FEC+DMC, and all-fluorinated electrolytes, respectively. As shown in Figures 10(a) and 10(b), the F content ultimately affects the robustness and composition of SEI and CEI. As shown in Figure 10(c), XPS is conducted to study the components of both SEI and CEI. Each component in the all-fluorinated electrolyte is an F donor, where ultrahigh LiF content (~90%) is found, inducing a more uniform and robust interphase. LiF has two essential functions here: (i) LiF acts as an excellent electronic insulator and prevents electrons from passing through the SEI layer, which has been the primary reason for the continuous capacity loss and consumption of electrolyte; (ii) LiF owns high interfacial energy of up to 73.28 meV/Å^2^, which constraints Li^+^ cation diffusion along with the interface and promotes the development of deposited Li in a direction parallel to, not perpendicular to, the electrode plane. It is worth noting that, unlike the SEI layer, the CEI formation relates not only to the electrolyte but also to active substances in the positive electrode. This involves solvent hydrogen abstraction, transition metal leakage, and oxygen-layer reaction into peroxides or superoxides. For the LiCoPO_4_ (LCP) electrode at the high voltage, DFT calculations show the migration of hydrogen (H) in all solvents to the cathode surface (Figures 10(d)–10(k)). And the reaction energies are the least favorable for 1,1,2,2-tetrafluoroethyl-2′,2′,2′-trifluoroethyl ether (HFE) solvent and the most favorable for EC solvent. EC solvent is a poor CEI layer former, because there is only a 0.91 eV barrier to release CO_2_ when EC^·^_(-H)_ radicals decompose, leaving only a small part of EC^·^_(-H)_ radicals to the second H extraction and further leading to polymerization. This eventually causes aggregation, while the polymer with high-ratio H is still prone to further decomposition. Due to the larger energy barrier for FEC solvent opening compared to EC solvent, FEC solvent will exist longer than EC solvent and have a chain reaction with FEMC and HFE solvents on the surface LCP cathode, resulting in F-rich interphase. Besides, the oxygen in the LCP cathode is banded with fluorinated radicals to suppress the formation of OH^−^, which effectively prevents the dissolution of transition metal. With the exception of the abovementioned nonflammable electrolyte system, a new type of nonflammable fluorinated solvent is designed based on EC and TEP. Zheng et al. [21] fabricated a fluorinated solvent, 2-(2,2,2-trifluoroethoxy)-1,3,2-dioxaphospholane 2-oxide (TFEP), applicated in HVLMBs. As displayed in Figure 10(l), by imitating the structure of EC molecule, combining the incombustibility of the phosphate group, and adding an F part, a fluorinated solvent named TFEP is synthesized to realize reversible Li^+^ transmission and high security. As displayed in Figure 10(m), TFEP solvent forms the CEI when the phosphorus center of the cyclic phosphate is nucleophilically attacked by O on the surface of O-rich metal (M-O). Pursuant proton transfer initiates the polymerization of TFEP solvent through a ring-opening reaction, resulting in polyphosphate formation on the NMC cathode. For the SEI formation, the process can be divided into the following three stages: (i) first, the TFEP solvent is predominantly reduced at ~1 V to form the initial SEI layer (LiPO_*x*_, Li_2_O, LiF, and polyphosphate); (ii) then, the further reduction of the FEMC at ~0.65 V leads to polycarbonate and Li_2_CO_3_; (iii) at last, the FSI anions are reducted to produce LiSON, Li_2_S, Li_2_SO_3_, and Li_2_S_2_O_3_. As shown in Figure 10(n), the 0.98 M LiFSI in FEMC and 0.95 M LiFSI in TFEP/FEMC electrolytes exhibit an oxidation voltage as high as ~4.9 V, considerably higher than conventional carbonate electrolyte (~4.4 V).

**Figure 11:**
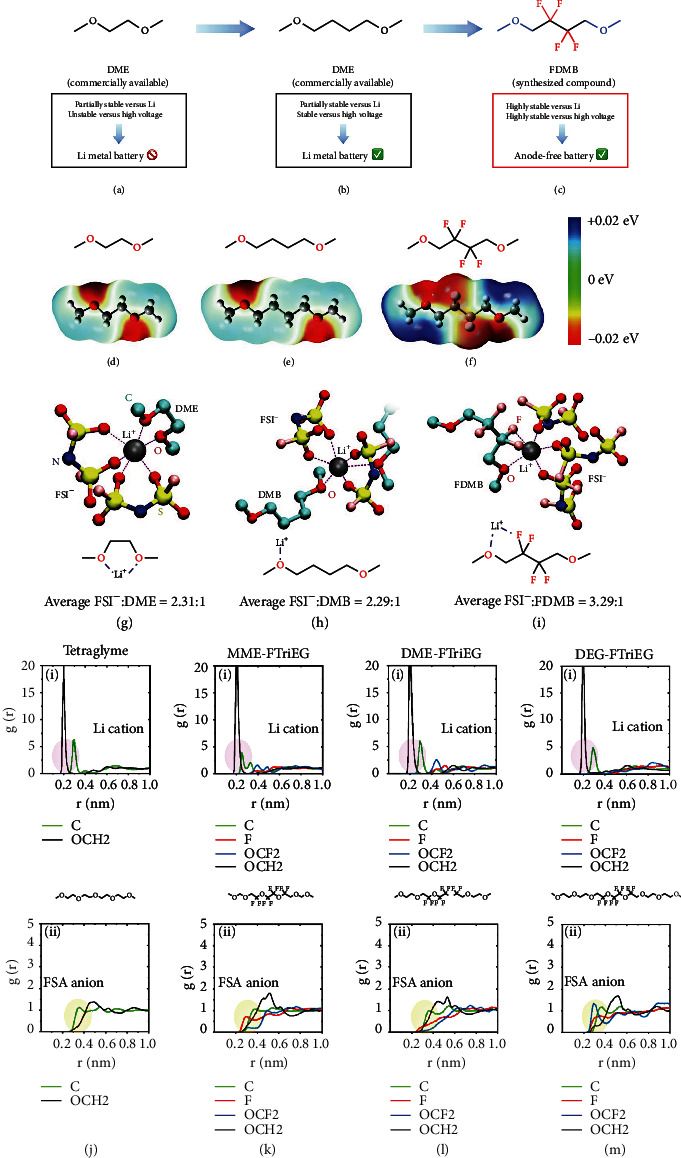
Design scheme and molecular structures of three liquids studied in this work: (a) DME, (b) DMB, and (c) FDMB. ESP comparison of (d) DME, (e) DMB, and (f) FDMB. Solvation structure of (g) 1 M LiFSI/DME, (h) 1 M LiFSI/DMB, and (i) 1 M LiFSI/FDMB given by MD simulations and the corresponding average ratio of solvation bindings from FSI- anions to those from solvents in the solvation sheath. Reproduced with permission [109]. Copyright 2020, Springer Nature. Radial distribution functions *g*(*r*) of 0.1 M LiFSA in the different electrolytes: (j) tetraglyme, (k) methyl methyl ether- (MME-) FTriEG, (l) DME-FTriEG, and (m) diethylene glycol- (DEG-) FTriEG. The highlighted regions draw attention to the differences between the spectra for both the Li^+^ cation and the FSA^−^ anion. (i) Li^+^ cation and (ii) FSA^−^ anion for the respective electrolytes. The anion spectra in part (ii) refer to the interaction between the fluorine atom in the FSA^−^ anion and the respective compounds. Reproduced with permission [110]. Copyright 2020, American Chemical Society.

Yu et al. [109] reported a fluorinated 1,4-dimethoxybutane (FDMB) as an electrolyte solvent by properly incorporating -CF_2_- parts. The ether backbone is selected here due to the excellent compatibility with Li metal to target the required electrolyte solvent. But ether-based electrolyte is intrinsically unstable above 4 V, limiting the cell performance at an extreme potential. Therefore, the following two pivotal design concepts are proposed to guarantee oxidation stability and high CE. First, the alkyl chain 1,2-dimethoxyethane (DME, Figure 11(a)) in the middle of the commonly used ether solvent structure is lengthened to obtain 1,4-dimethoxybutane (DMB, Figure 11(b)). The longer alkyl chain owns sufficient stability and enough ability to conduct Li^+^ and dissolve lithium salts. Second, the introduction of F groups can further enhance the oxidation potential and Li anode compatibility. However, it is well known that the solvation ability of the ether can be kept only when the -F group is far away from the -O- group. Therefore, the -CF_2_- group can only replace the center of DMB to achieve the purpose of being far away from the -O- group (Figure 11(c)). As a result, the FDMB solvent is expected to be stable to both high-voltage cathodes and Li anode. As shown in Figures 11(d)–11(f), the interaction of the Li-F band is evaluated using electrostatic potential (ESP) simulations, which are closely related to noncovalent interactions. For DME and DMB molecules, the negative charges are all distributed around the O atoms. The FDMB molecule exhibits different electron distribution, where the negative charge concentrates not only around O atoms but also on F atoms. To further prove the solvation structures, molecular dynamics (MD) calculations for different electrolyte solvents are studied (Figures 11(g)–11(i)). The coordination of DME molecule and Li^+^ cation is like the “clamping” of two -O- groups. For DMB molecules, most Li^+^-solvent structures are “linear,” in which there is only one -O- group combined with one Li^+^ cation. Unlike DME or DMB molecules, a five-membered ring is observed in the LiFSI/FDMB electrolyte, where the Li^+^ cation is simultaneously bonded to O and F atoms. Based on the above molecular design strategy, a new class of fluorinated ether electrolytes that combine the oxidative stability of HFEs with the ionic conductivity of ethers in a single compound are synthesized [110]. At room temperature, the ionic conductivity can reach 2.7 × 10^−4^ S/cm with a high oxidation potential of 5.6 V. MD calculations are conducted to study the solvation structure in these fluorinated solvents. *g*(*r*) for Li^+^ cation and FSA^−^ anion in fluorinated triethylene glycol (FTriEG) and tetraglyme is studied in Figures 11(j)–11(m). The O atoms on the -OCH_2_- group in the tetraglyme compound are most likely to be adjacent to the Li^+^ cation (Figure 11(j)). Comparing *g*(*r*) of Li^+^ cation in Figures 11(k)–11(m), the ether group adjacent to the Li^+^ cation can be found. As displayed in Figures 11(k) and 11(l), as the methylene group moves to the ethylene spacer, the interference of carbon in the first solvation shell is smaller, and the possibility of carbon in the next solvation shell increases. The *g*(*r*) results also show that both H and F atoms from solvent molecules contribute obviously to the interaction with the FSA^−^ at 1 M conventional concentration. Therefore, the fluorinated segment in the molecule interacts with the fluorinated anion by “fluorous effect,” which suppresses anion migration. Moreover, Figure 11(m) shows that as the length of the ether increases, the probability of the appearance of -OCF_2_- in the Li^+^ solvation shell decreases. This also explains why longer ether chains have higher ionic conductivities.

**Figure 12:**
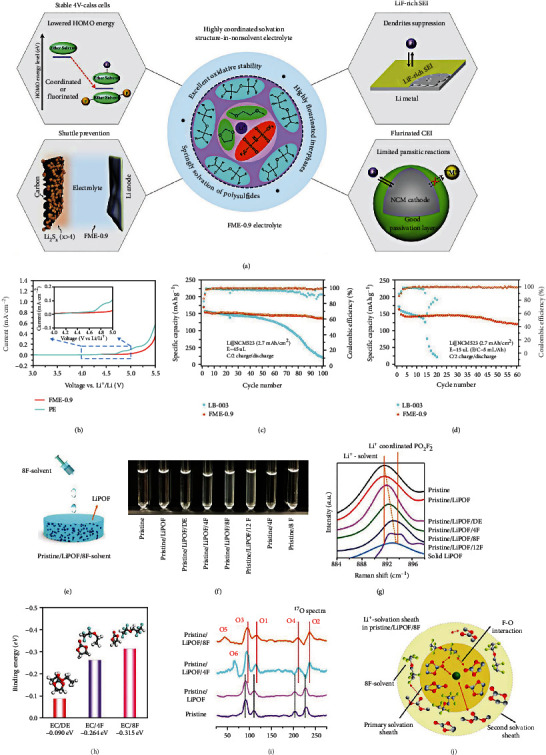
(a) Schematic illustration of advanced battery functions realized by FME-0.9. (b) LSV curves of pristine electrolyte and FME-0.9 at 0.5 mV s^−1^. Cycling performance with high areal loadings (2.7 mAh cm^−2^) under (c) flood electrolyte (45 *μ*L) and (d) lean electrolyte (15 *μ*L). Reproduced with permission [111]. Copyright 2020, Elsevier B.V. (e) Schematic diagrams of recrystallization of LiPO_2_F_2_ upon addition of the 8F solvent into a pristine/LiPOF electrolyte. (f) Digital photographs of a pristine electrolyte upon addition of LiPO_2_F_2_ and different kinds of solvents. (g) Raman spectra of pristine, pristine/LiPOF, pristine/LiPOF/DE, 4F/8F/1,1,2,2,3,3,4,4-octafluoro-5-(1,1,2,2-tetrafluoroethoxy) pentane (12F) solvent, and bare LiPO_2_F_2_. (h) Theoretical binding energies and geometry structures calculated through first-principle calculations among DE/EC, 4F/EC, and 8F/EC in a carbonyl-coordinated manner. (i) ^17^O nuclear magnetic resonance (NMR) spectra for pristine, pristine/LiPOF, pristine/ LiPOF/4F, and pristine/LiPOF/8F. (g) Schematic illustration of the Li^+^ solvation sheath and related solvent interaction of Li^+^-coordinated units and F-O interaction in pristine/LiPOF/8F. Reproduced with permission [112]. Copyright 2021, American Chemical Society.

Zhao et al. [111] developed a novel fluorinated electrolyte with an advanced solvation structure to restrain Li dendrite generation and maintain stable interphase of high-voltage cathodes. As displayed in Figure 12(a), the novel electrolyte is fabricated by adding a high-ratio nonsolvent TTE to the traditional ether-based solvent. The specific composition is 1 M LiTFSI salt in DOL/DME/TTE with a 90% mass proportion of TTE (DOL/DME, *v*/*v* = 1/1). The unique solvation structure of 1 M LiTFSI in TTE/DME/DOL with TTE/(DME/DOL, 1/1, *v*/*v*) mass ratio of 9/1 (FME-0.9) electrolyte endows it with outstanding oxidative stability. As shown in Figure 12(b), the LSV shows that the FME-0.9 electrolyte with 1 M lithium salt concentration remains stable until 4.5 V, which can support high-voltage cathodes. The high oxidation potential is mainly due to the following two main reasons. First, the highly coordinated Li^+^ keeps the lone pair of electrons in the O atom of the solvent molecule away from the cathode's capture, thereby decreasing its HOMO energy level. Second, the lower HOMO of the TTE solvent exhibits outstanding Li metal stability, which is attributed to the F substituent with strong electron-withdrawing reducing the electron density. As shown in Figure 12(c), the LiNi_0.5_Mn_0.2_Co_0.3_O_2_ (NCM532)||Li cell achieves excellent capacity retention of 80% with 50 *μ*m Li metal and 45 *μ*L FME-0.9 electrolyte at 1/2 C. Conversely, the capacity quickly decays to only 15% of the initial capacity in the 1 M LiPF_6_ in EC/DMC/EMC = 1/1/1, *v*/*v*/*v* (LB003). Then, as the amount of electrolyte reduces to 15 *μ*L, the NCM532||Li cell using FME-0.9 electrolyte still exhibits high capacity retention of 81% after 60 cycles (Figure 12(d)). Nevertheless, the battery utilizing the LB003 undergoes rapid capacity degradation and fails at the 20^th^ cycle. Moreover, there is also a distinctive function of the fluorinated ether, which plays as a destabilizer to change the Li^+^ solvation structure and reduce the reciprocity between Li^+^ ions and carbonyl in traditional electrolytes. Deng et al. [112] reported that a fluorinated ether as a destabilizer facilitates the recrystallization of LiPO_2_F_2_ (LiPOF) from the electrolyte for concurrent surface protection on both the anode and the cathode (Figure 12(e)). As shown in Figure 12(f), the conventional carbonate electrolyte keeps clear and transparent when 2 wt.% LiPOF is introduced. However, after further adding 8F solvent, the electrolyte becomes turbid, indicating the precipitation of LiPOF. In Raman spectra (Figure 12g), the original electrolyte displays a classical enlarged peak at 891.6 cm^−1^ related to the Li^+^ solvation structure. In comparison, the pure LiPOF shows a distinct peak at 893.5 cm^−1^ on the Li-related PO_2_F_2_^−^ anions without a solvation structure. As the F concentration in fluorinated ether increases, the major peaks slightly transfer, which means that more Li is coordinated with PO_2_F_2_^−^, and the Li^+^ solvation structure has been disturbed. As shown in Figure 12(h), the DFT calculations are conducted to study the interaction between additives and EC solvent. The binding energy of DE-EC is only -0.09 eV, considerably lower than those of 1-ethoxy1,1,2,2-tetrafluoroethane (4F)-EC (0.264 eV) and 1,1,2,2-tetrafluoroethyl-2,2,3,3-tetrafluoropropylether (8F)-EC (-0.315 eV), indicating stronger interaction between fluorinated ether with EC solvent compared with that of diethyl ether (DE). Therefore, it is apparent that the attraction ability from fluorine to carbonyl sites on EC solvent increases along with the number of F atoms. In addition, the level of salting out of LiPOF is only affected by the solvent type rather than the lithium salt, indicating that the solvent competition influences the shell structure around Li^+^. As shown in Figure 12(i), after adding 4F and 8F solvent, the signals of ^17^O correspond to ethereal (O2 or O4 site) and 2 equiv of carbonyl (O1 or O3) in EC+DMC electrolyte migrating to the large position, which demonstrates a shielding influence on Li^+^ ion. As displayed in Figure 12(g), when fluorinated ether is added to the original electrolyte, it will participate in the Li^+^ solvation structure. Robust F-O interaction between EC+DMC and fluorinated ether is formed.  Table 5 lists the cycling performance of the HVLMBs with different fluorinated electrolytes.

#### 3.1.1. Section Summary

This section discusses recent development in fluorinated electrolytes, including FEC, FEMC, HFE, TFEP, FDMB, FTriEG, and TTE. Overall, the C–F bonds in organic compounds possess considerable effects on the LUMO and HOMO energy of the solvent molecule and positively influence the interfacial chemistry in HVLMBs. In addition, these fluorinated solvents are weakly flammable and even nonflammable, which can effectively improve thermal stability and increase electrode passivation. In the future, systematically enhancing the interfacial electrochemical/chemical compatibility between fluorinated solvents and high-voltage cathode/Li metal anode is conducive to further guaranteeing the long-time cycling life. Moreover, the specific energy density, CE, and cycle life of practical pouch cells using fluorinated electrolytes are expected to be further explored under commercial conditions.

### 3.2. Ionic-Liquid Electrolytes

Ionic liquids have exhibited stupendous potential for applications in lithium-based cells and acted in multifunctional roles because of their highly different physicochemical features from inorganic salts and molecular solvents. They can be used as electrolyte solvents to substitute conventional carbonate-based solvents to enhance battery safety due to their nonflammable and nonvolatile properties. In particular, FSI^−^/TFSI^−^-based ionic liquids with efficient ion transfer have attracted widespread attention. First, the highly delocalized charge distribution remarkably decreases the interaction between cations and anions, enhancing the Li^+^ fluidity in the ionic-liquid electrolyte. Then, the small ion sizes of FSI^−^ (0.26 nm) and TFSI^−^ (0.38 nm) anions enable the decrease in the viscosity of ionic-liquid electrolytes, accelerating the Li^+^ transfer in the electrolyte.

**Figure 13:**
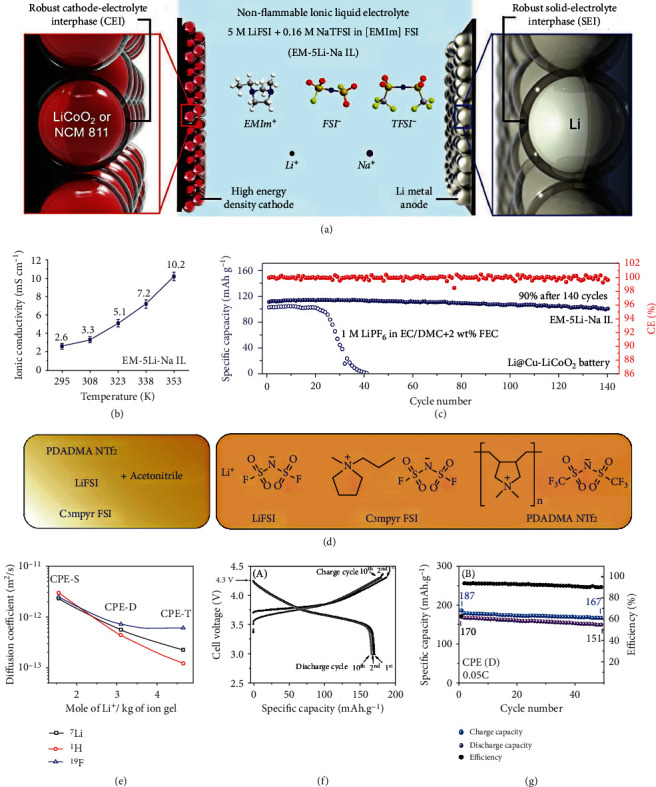
(a) Schematic illustration of the battery configuration and electrolyte composition of the EM–5Li–Na IL electrolyte. (b) Ionic conductivities of EM–5Li–Na IL at various temperatures. (c) Cyclic stability of Li@Cu–LCO batteries using EM–5Li–Na IL and organic electrolytes at 0.7 C. Reproduced with permission [56]. Copyright 2020, WILEY-VCH. (d) Schematic illustration of composite electrolyte preparation along with used materials. (e) Diffusion coefficient of ^7^Li (Li^+^, black), ^1^H (C3mpyr^+^, red), and 19F (NTf_2_^−^, blue) of single CPE (CPE-S), CPE-D, and triple CPE (CPE-T) composite electrolytes measured by solid-state NMR. (f) Charge-discharge profiles after 1, 2, and 10 cycles for Li||NMC cells containing CPE (D) composite electrolyte. (g) Corresponding cycling performance for 50 cycles at 0.05 C (0.03 mA) and 50°C. Reproduced with permission [123]. Copyright 2019, American Chemical Society.

Ionic liquid has been considered a member of the most prospective electrolyte candidates for the HVLMBs due to its low volatility, excellent flame retardancy, and wide electrochemical window. As displayed in Figure 13(a), Sun et al. [56] reported an advanced ionic-liquid electrolyte for HVLMBs. This electrolyte is comprised of bis(fluorosulfonyl)imide (FSI) anions and 1-ethyl-3-methylimidazolium (EMIm) cations, with the additive of sodium bis(trifluoromethanesulfonyl)imide (NaTFSI), denoted as EM–5Li–Na IL electrolyte. The EM–5Li–Na IL electrolyte owns a viscosity as low as 125 mPa s at room temperature, half of the Py13-based ILs electrolyte. As shown in Figure 13(b), the EM–5Li–Na IL electrolyte exhibits an ion conductivity of ~2.6 mS cm^−1^ at 25°C, considerably higher than other ionic-liquid electrolytes (1.0-1.2 mS cm^−1^) for LIBs. Due to lower viscosity at high temperatures, the ionic conductivity is further enhanced to over 10 mS cm^−1^. Compared with traditional carbonate electrolytes, the EM–5Li–Na IL electrolyte exhibits high safety owing to its superhigh thermal stability and nonflammability. As shown in Figure 13(c), the LiCoO_2_ (LCO)||Li cell utilizing the EM-5Li-Na IL electrolyte exhibits excellent capacity retention and high average CE at 0.7 C. On the contrary, the cell with traditional carbonate electrolyte undergoes rapid CE and capacity decay, indicating the fast loss of active Li in the organic electrolyte. Overall, the EM–5Li–Na IL electrolyte is novel for the following points. First, the EMIm cations are vital for accomplishing high rate capacities under high-loading conditions. Then, the high concentration of LiFSI salts in the EM–5Li–Na IL electrolyte is relevant to excellent battery performances. At last, NaTFSI is first exploited as a novel additive for improving the LMB performances. The ionic-liquid electrolyte based on the poly-ILs can support HVLMBs to work at a higher voltage as compared with that based on the EMIm. Wang et al. [123] mixed polymerized poly-ILs and poly(ionic liquid)s as well as electrospun fiber carrier to prepare an electrolyte with high-ratio ionic liquid, contributing to a dramatic enhancement of Li^+^ transference number. With this electrolyte, the Li metal cells with NMC and LiNi_1‐*x*‐*y*_Mn_*x*_Al_*y*_O_2_ (NMA) cathodes exhibit excellent cycling performances under a high voltage of 4.5 V. Polyvinylidene fluoride (PVDF) nanofibers can be used as an outstanding mechanical skeleton to prepare flexible and thin composite polymer electrolyte (CPE). As shown in Figure 13(d), the CPEs are synthesized by solvent pouring. Figure 13(e) displays the diffusion coefficients of each component tested by the pulsed-field gradient nuclear magnetic resonance (PFG-NMR) methods, and Figure 13(f) exhibits the voltage profiles during charging and discharging of the LCO||Li battery. The specific capacity of the first cycle for the cell using the double CPE (CPE-D) electrolyte is 170 mAh g^−1^. The CE of the battery exceeds 94% after 2 cycles. Within 50 cycles, the specific capacity decays very slowly to 150 mAh g^−1^, as shown in Figure 13(g). The low CE in the first cycle indicates irreversible capacity, which is ascribed to the degradation of electrolyte, the consumption of active Li, and the formation of the SEI layer.  Table 6 shows the cycling performances of the HVLMBs with different ionic-liquid electrolytes.

#### 3.2.1. Section Summary

In this section, recent development in the ionic-liquid electrolytes has been discussed, including TFSI^−^, FSI^−^, EMIm^+^, C3mpyr^+^, and NTf_2_^−^. Ion conductivity is regarded as one of the most critical indicators of ionic-liquid electrolytes used in HVLMBs [124]. Ionic liquids are constituted of anions with decreased charge density and tiny size and the cations with electron-donating functional groups and delocalized unsaturated chemical bonds [125, 126]. This distinctive composition significantly weakens the interaction between anions and cations, resulting in large conductivity. Nevertheless, the ion transfer in ionic-liquid electrolytes is deeply influenced by viscosity. Higher viscousness of the ionic-liquid electrolyte leads to slow transfer of Li cations in the electrolyte. Apart from viscosity, the ion radius and carrier distribution density of ionic-liquid electrolytes also affect ion transfer. The ion transfer rate is inversely proportional to the ion size and proportional to the distribution density of the charge carriers. Because the ionic-liquid electrolyte is fully constituted of cations and anions, the amount of charge carriers is adequate, but the carrier number decreases due to the uncontrollable ion association, leading to reduced ion conductivity. In the future, several issues hindering the industrial commercialization of ionic-liquid electrolytes are urgent to solve, such as expensive cost, large viscosity, and inferior electrode affinity.

### 3.3. Sulfone Electrolytes

Sulfone is an inexpensive by-product by numerous chemical manufacturers, as produced by tons and generally used in high-temperature industry for organic and inorganic compound preparation as reaction and extraction solvents, and for fungicide treatments [138–140]. Compared with the carbonyl group in carbonate solvents and the ether group in ether solvents, the sulfonyl group with more significant electron-withdrawing effect provides a lower HOMO energy value, causing higher oxidation stability. In addition, sulfone as an electrolyte solvent candidate owns several advantages such as low flammability, large dielectric permittivity, and excellent cathode compatibility. However, the commercial application of the sulfone electrolytes is prevented by large viscosity, high melting point, and low affinity for Li anode, which can be resolved by grafting functional groups and mixing with other solvents/additives.

**Figure 14:**
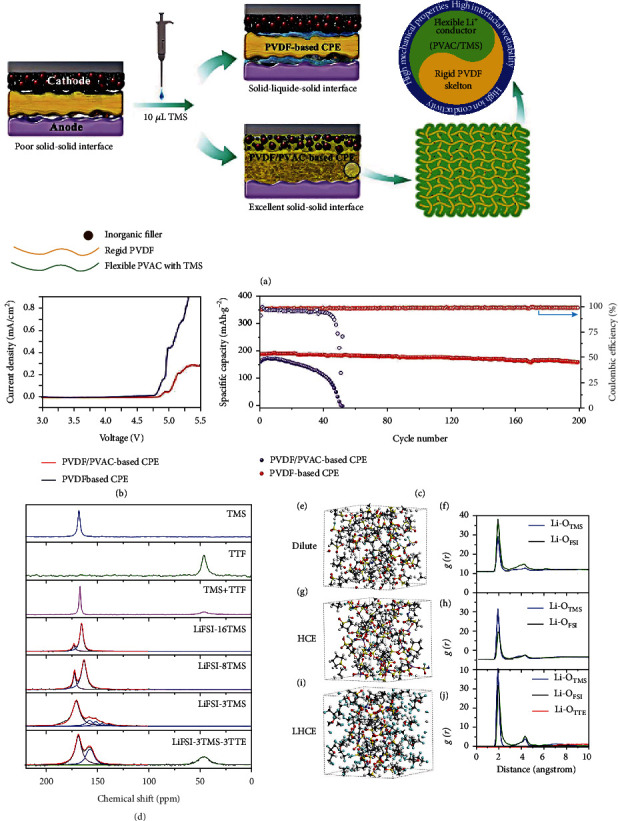
(a) Schematic illustration of the different interface characteristics between PVDF-based CPE and PVDF/PVAC-based CPE. (b) LSV plots of PVDF-based CPE and PVDF/PVAC-based CPE. (c) Cycling performance of LCO/PVDF/PVAC-based CPE/Li and LCO/PVDF-based CPE/Li batteries. Reproduced with permission [141]. Copyright 2020, WILEY-VCH. (d) Natural abundance ^17^O-NMR spectra of different solvents and electrolytes collected at 60°C. AIMD simulation snapshots of (e) LiFSI-8TMS, (g) LiFSI-3TMS, and (i) LiFSI-3TMS-3TTE. Radial distribution functions *g*(*r*) of Li-O_FSI_, Li-O_TMS_, and Li-O_TTE_ pairs in (f) dilute electrolyte, (h) HCE electrolyte, and (j) LHCE electrolyte. Reproduced with permission [142]. Copyright 2018, Elsevier B.V.

Sulfone is low-cost, with an electrochemical window exceeding 5 V, and is considered a potential electrolyte for the HVLMBs. Yu et al. [141] introduced tetramethylene sulfone (TMS) solvent to PVDF-polyvinyl acetate-based (PVAC) for safe and efficient HVLMBs. This interaction between the molecules in SPE remarkably improves Li^+^ conductivity and wetting behavior at the electrolyte-electrode interface. As displayed in Figure 14(a), PVDF/PVAC/Li_6.4_La_3_Zr_1.4_Ta_0.6_O_12_ (LLZT) wetted by TMS solvent is prepared for 4.5 V LCO||Li cell. As shown in Figure 14(b), the oxidation potential of PVDF-based CPE is ~4.75 V. However, it turns out that the addition of PVAC enables the CPE to maintain steady at 4.85 V, as verified by the lower and stabler current of the modified CPE. This is due to excellent interfacial compatibility and high stability of the PVAC and TMS. The LCO||Li cells using the two electrolytes are tested to compare the electrochemical performances at the high voltage. As displayed in Figure 14(c), the LCO||Li cell with the PVDF-based CPE electrolyte undergoes rapid capacity decay and only survives 50 cycles. Comparatively, the LCO||Li battery with PVDF/PVAC-based CPE electrolyte exhibits a modest capacity decay of 15% after 200 cycles. The charge-discharge curves of the LCO||Li cell with this electrolyte demonstrate a capacity of 190.8 mAh g^−1^ at the initial circle and maintain over 160 mAh g^−1^ after 200 cycles due to the low polarization. Conversely, remarkable polarization results in a severe decrease in the initial specific capacity of the cell using PVDF-based CPE (164.4 mAh g^−1^). In particular, the coordination between TMS and Li^+^ can also contribute to interfacial formation. Ren et al. [142] developed a concentrated sulfone-based electrolyte to achieve a high CE of over 98%. In addition, by introducing a fluorinated ether TTE, low viscosity and high wettability are obtained in the LHCE, which further enhances the CE and cell stability at an extreme potential. As shown in Figure 14(d), after TTE solvent addition, the evolution of the Li^+^ solvation structure is studied through NMR. The signal of O atoms in FSI^−^ ion migrates from 172.4 to 171.8 and 170.1 ppm as the LiFSI salt/TMS solvent enhances from a molar ratio of 1 : 16 to 1 : 8 and 1 : 3. This is because the ion-dipole interactions between O atom and Li^+^ ion gradually increase when the concentration of LiFSI salt improves. The signal of O migrates from 164.9 to 162.6 ppm with TMS as the solvent, suggesting the number of TMS associated with Li^+^ cation increases. For the HCE, the signal of the O atoms in TMS solvent has shifted more obviously from 138.0 to 158.0 ppm, which represents different Li^+^ solvation structures. The O atoms in FSI^−^ anion exhibit a significant migration to 170.1 from 168.1 ppm. In addition, with TMS as the solvent, the O signal at 156.7 ppm gets more evident as TTE solvent owns low interaction with both FSI^−^ anion and Li^+^ cation but exhibits strong coordination ability with TMS solvent. Figures 14(e)–14(j) show the simulation snapshots of the three electrolyte systems. For all systems, the LiFSI salt is associated with TMS molecule instead of TTE molecule. *g*(*r*) for Li^+^-O_TTE_ and Li^+^-O_TMS_ pairs is studied by *ab initio* molecular dynamics simulations. Regarding the three electrolytes, the peak of Li^+^-O_TMS_ remains at 1.95 A° (Figures 14(f), 14(h), and 14(j)), suggesting that TMS solvent becomes the first coordination structure for the Li^+^ cation. This is attributed to the robust interaction between LiFSI salt and TMS solvent. In addition, the TTE solvent is not associated with Li^+^ cation in the LHCE. Bader charge analysis is conducted to investigate the possible charge transfer between the lithium salt and solvents. Compared with LiFSI, TMS, and the TMS/LiFSI pair, the interaction between TTE and the anode surface is low, demonstrating that the TTE molecule is inert and is not reduced. However, TMS/LiFSI pair and TMS are slightly reduced by obtaining the fractional charges of 0.40 and 0.22 |e|, respectively. This means that both are possibly reduced and form interfacial compositions.

**Figure 15:**
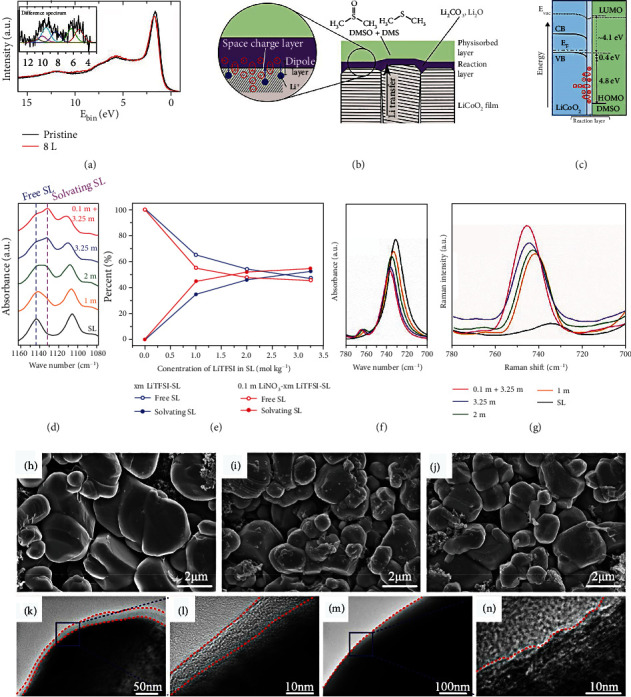
(a) Valence band spectra for pristine LCO and after adsorption of 8 Langmuirs of DMSO. (b) Illustration of the processes at the LCO/DMSO interface. (c) Energy level diagram of LCO in contact with DMSO. Reproduced with permission [143]. Copyright 2016, American Chemical Society. (d) FTIR spectra of SL's symmetric SO_2_ stretch with different electrolytes. (e) Relative intensities of FTIR peaks at 1144^−1^ and 1130 cm^−1^ in the LiTFSI-SL electrolytes. (f) FTIR spectra of TFSI^−^'s S-N stretch with different electrolytes. (g) Raman spectra for TFSI^−^ of various electrolytes. Reproduced with permission [144]. Copyright 2020, WILEY-VCH. SEM images of (h) the fresh LCO cathode and 4.55 V high-voltage cycled LCO cathodes with (i) the EC+DMC electrolyte and (j) MSM electrolyte. TEM images of the high-voltage cycled LCO cathodes with (k, l) EC+DMC electrolyte and (m, n) the MSM electrolyte. Reproduced with permission [145]. Copyright 2019, American Chemical Society.

Spaeth et al. [143] developed a dimethyl sulfoxide (DMSO) electrolyte to generate the advanced interface between the solvent adsorbate and high-voltage LCO cathode.  Figure 15(a) shows the valence band spectrum of the fresh LCO and the spectrum after 8 Langmuirs of DMSO adsorbed. For the fresh LCO, a composition of about 1.5 eV is distributed to the Co 3d state. The features from 3 to 8 eV can be distributed to the overlap of the O 2p and Co 3d states and the pure O 2p state. The offset between the valence band of fresh LCO and the HOMO of DMSO mainly comes from the valence band spectrum: after DMSO exposure, in addition to the energy migration caused by band bending, the change in signal shape is also found, and the change in signal is mainly contributed by the physically absorbed DMSO in 8 Langmuirs (inset in Figure 15(a)). Since no change in the oxidation state of cobalt is observed during the experiment, it can be concluded that after the initial reaction, Li is deintercalated from the volume of the fresh LCO film. This effectively inhibits the transportation of Li, probably at the grain boundary of the (0 0 3) textured film, as shown in Figure 15(b). As displayed in Figure 15(c), 4 Langmuirs of DMSO after exposure is displayed. The negative charge is distributed on the surface of the LCO cathode, while the positive charge remains in the internal reaction layer. As compared with the DMSO, the sulfolane (SL) can dissolve more lithium salts, which makes it promising to be applied in HCEs and LHCEs. By introducing HFE and LiNO_3_ into the concentrated sulfone-based electrolyte, Fu et al. [144] developed a novel LHCE to inhibit the growth of Li dendrite and achieved an excellent CE of ~99% for both high-voltage NMC811 cathode and Li metal anode. The Li^+^ solvation structures of various electrolytes are studied by Fourier-transform infrared (FTIR), as displayed in Figure 15(d). The apparent peak at 1144 cm^−1^ is associated with the SO_2_ band of free sulfone solvent in the 1160~1080 cm^−1^ of the FTIR. As LiTFSI salt concentration increases, a new peak at 1130 cm^−1^ can be found, which corresponds to the solvated sulfone solvent. For the various LiTFSI salt concentration electrolytes with or without 0.1 M LiNO_3_ salt, the peak intensities of 1130 and 1144 cm^−1^ are studied in Figure 15(e). This enables the analysis of the degree of solvation of Li^+^ cation and sulfone solvent. As shown in Figures 15(f) and 15(g), Raman spectroscopy and FTIR in the electrolytes of various salt concentrations are tested. As the concentration of LiTFSI increases from 1 M to 3.25 M, the upward shift of the S-N band peak to high wavenumber suggests that more TFSI- participates in the Li^+^ solvation structure assigned to the S-N band. After adding LiNO_3_ salt to the electrolyte, the position of the S-N band peak is further upward shift than 3.25 M LiTFSI-SL, indicating that the NO_3_^−^ group strengthens the interactions of Li^+^ cation and TFSI^−^ anion. MD simulations are also conducted to analyze the Li^+^ solvation shell. The calculation results exhibit that each Li^+^ in 3.25 M LiTFSI-SL electrolyte is solvated by ~3.44 SL molecules and 1.03 TFSI^−^, and the solvation sheaths are linked with SL solvents. After adding 0.1 M LiNO_3_ into 3.25 M LiTFSI-SL electrolyte, the concentration of TFSI^−^ anions increases around NO_3_^−^ anions, and more TFSI^−^ links to the Li^+^ solvation sheaths, which enriches the combining sites for Li^+^ hopping motion in the concentrated electrolytes. By theoretical calculation, Kong et al. [145] reported a progressive electrolyte to remarkably improve the electrochemical performances of the HVLMBs. This electrolyte solvent comprises four solvents: FEC/DMC/HFE/dimethyl sulfone (MSM). Figures 15(h)–15(j) show the SEM pictures of the pristine and cycled LCO cathodes at 4.55 V. The surface of the pristine LCO cathode is very flat, but the surface is very rough in the EC+DMC electrolyte. However, regarding the MSM electrolyte, the cathode displays a smooth and uniform surface. By the HRTEM (Figures 15(k)–15(n)), the cathode cycled in the EC+DMC produces a 10-20 nm deposited layer on the surface of LCO. On the contrary, the LCO cathode has a flat surface in the MSM electrolyte.  Table 7 shows the cycling performance of the HVLMBs with different sulfone electrolytes.

#### 3.3.1. Section Summary

In this section, recent development in the sulfone electrolytes has been discussed, including TMS, DMSO, SL, MSM, and MPS. Overall, sulfone-based solvents own high dielectric constant (>40) and high oxidation potential (>5 V *vs.* Li/Li^+^), exhibiting stability with high-voltage cathodes. Nevertheless, sulfone-based solvents show poor wettability and inferior compatibility with Li metal anode. In the future, developing novel sulfones by grafting functional groups is expected to own low viscosity and high affinity with Li anode. Moreover, mixing with other solvents/additives, such as FEC, HFE, DMC, and LiNO_3_, can achieve the same improvement under the guidance of theoretical calculations.

### 3.4. Nitrile Electrolytes

As proved above, the excellent antioxidation stability of the sulfones can be attributed to the powerful intrinsic stability of the sulfone group against oxidation at an extreme potential. On the contrary, the antioxidation stability of nitrile-based electrolytes is attributed to the prioritized decomposition on the surface of the high-voltage cathode, generating an effective protective layer of -C≡N/transition metal complexes to avoid the intimate contact between other species and catalytic surfaces of the cathode. In addition, similar to carbonate functional groups in alkyl carbonate molecules with large dielectric constants and dipolar moments, the terminal -C≡N functional groups are excellent nucleophilic sites for coordinating Li^+^.

**Figure 16:**
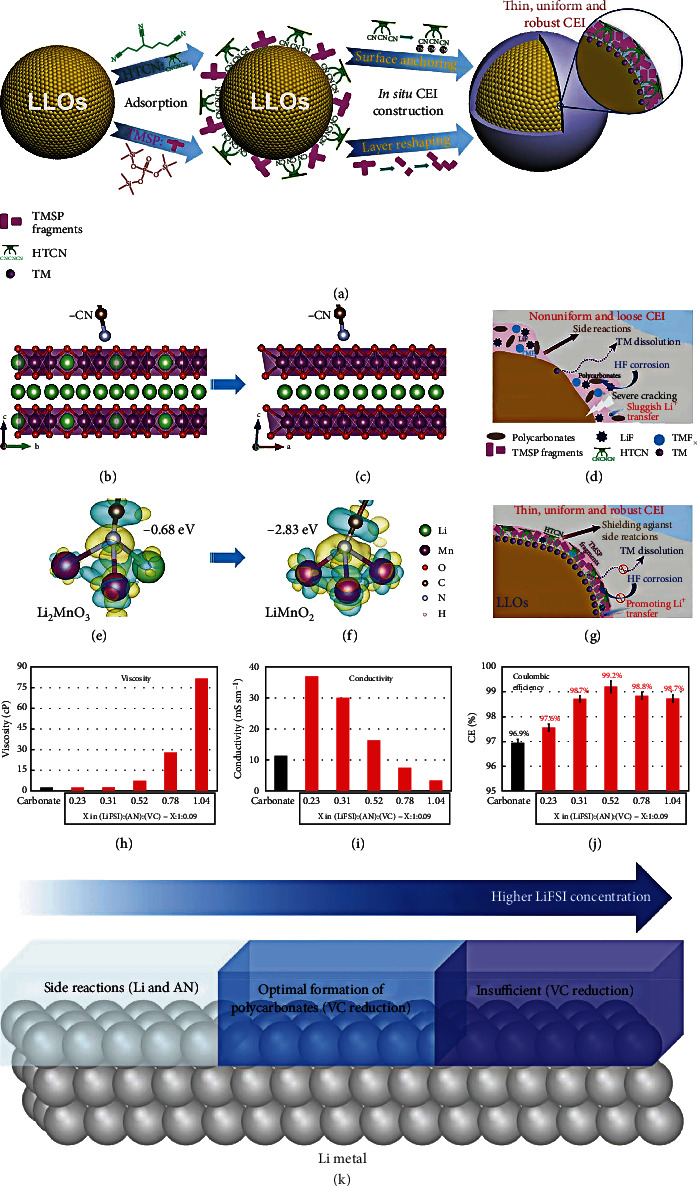
(a) Schematic illustration of the synergistic effects of HTCN+TMSP on adjusting the CEI structure and cathode electrochemistry. (b, c) The configurations of -C≡N binding with the Li_2_MnO_3_ crystal domain in LLO upon the electrochemical evolution from the pristine to the monoclinic manganese-based LiTMO_2_ structure. The differential charge densities of -C≡N binding with (e) the pristine Li_2_MnO_3_ crystal domain and (f) the evolved monoclinic manganese-based LiTMO_2_ structure. (d, g) Schematic illustrations of the mechanism of BE-induced and BE/T/H-induced CEI on LLOs. Reproduced with permission [152]. Copyright 2020, WILEY-VCH. (h) Viscosities, (i) conductivities, and (j) CEs of the electrolytes for molar ratio of [LiFSI]: [AN]: [VC] = *X* : 1 : 0.09, where *X* ranged from 0.23 to 1.04. (k) Schematic representation of Li interfacial phases in the LAV electrolytes with increasing LiFSI concentration. Reproduced with permission [153]. Copyright 2020, WILEY-VCH.

There are a lot of advantages to nitriles, such as high thermal stability, excellent anode stability, and wide liquid temperature range. The most prominent feature is the wide electrochemical window. The oxidation stability of mononitrile can reach 7 V, which enables the full support of the HVLMBs. Zhao et al. [152] reported tris(trimethylsilyl) phosphate (TMSP) and 1,3,6-hexanetricarbonitrile (HTCN) as electrolyte additives to generate robust CEI, which supports high-performance Li_1.13_Mn_0.517_Ni_0.256_Co_0.097_O_2_ (LLO) electrode. The HOMOs of EC, DEC, HTCN, and TMSP are -8.44, -8.21, -9.08, and -7.94 eV, respectively, demonstrating the excellent high-voltage stability of HTCN and the prioritized oxidation of TMSP. HTCN owns three -C≡N groups with lone-pair electrons to combine with transition metal atoms and thus can anchor surface transition metal atoms even at an extreme potential as a stable CEI framework. In addition, the prioritized decomposition of TMSP can provide beneficial groups to participate in the *in situ* formation of the CEI. As shown in Figure 16(a), HTCN and TMSP as binary additives are introduced into the conventional electrolyte (1 M LiPF_6_ in EC/DEC). A synergistic effect is realized to generate the CEI layer, alleviate irreversible side reactions, and inhibit transition metal dissolution. Both TMSP and HTCN take precedence in covering the cathode surface to separate electrolyte and electrode, which effectively prevents side reactions between the two components. The three -C≡N groups in HTCN solvent are firmly attached to the electrode interface to prevent transition metal dissolution. In addition, owing to the high oxidation potential of HTCN solvent, the electrolyte exhibits a wide electrochemical window. As displayed in Figures 16(b), 16(c), 16(e), and 16(f), the binding energy of HTCN-Li_2_MnO_3_ (0 0 1) and HTCN-LiMnO_2_ (0 0 1) is calculated. For either LiMnO_2_ or Li_2_MnO_3_, the charge density exhibits the accumulation of electrons in the region between Mn and N atoms. In contrast, the C atom in the -C≡N group shows reduced electron density, indicating that the electrons move from the -C≡N group to the Mn atom. The binding energy of HTCN-LiMnO_2_ is -2.83 eV, remarkably larger than that of HTCN-Li_2_MnO_3_ (-0.68 eV). As shown in Figures 16(d) and 16(g), using the conventional electrolyte for LLO cathodes has several main disadvantages, including the dissolution of transition metal, the degradation of LLO structure, and the severe side reactions, which are proved by the nonuniform and thick CEI. These issues can be significantly solved by introducing HTCN and TMSP additives to generate uniform and thin CEI. Compared with the HTCN, acetonitrile (AN), with a much lower cost, is promising for practical applications. Peng et al. [153] reported an AN-based electrolyte with HCE strategy and VC additive to prevent the depletion of Li^+^ ion at large current densities. As shown in Figure 16(h), the electrolyte with a molar ratio of [LiFSI]: [AN]: [VC] = 0.23 : 1 : 0.09 is 2.18 cP, which is comparable to commercial electrolytes (2.19 cP). As a rule, the larger viscosity is attributed to the enhanced electrostatic interaction among ion pairs, which may increase the difficulty of ion transportation and thus lead to lower conductivity, as displayed in Figure 16(i). Highest CE at a molar ratio of [LiFSI]: [AN]: [VC] = 0.52 : 1 : 0.09 (LAV) is achieved for different LiFSI salt concentrations, as shown in Figure 16(j). For the LAV electrolyte with lower salt concentration, side reactions between AN solvent and Li metal predominate. For the LAV electrolyte with higher salt concentration, although the side reactions are inhibited by decreased free AN solvent, the SEI formation is also suppressed. Figure 16(k) schematically illustrates the change of the Li metal interface with the salt concentration in the LAV electrolyte.  Table 8 shows the cycling performance of the HVLMBs with different nitrile electrolytes.

#### 3.4.1. Section Summary

In this section, recent development in the sulfone electrolytes has been discussed, including TMS, DMSO, SL, MSM, and MPS. The oxidation voltage of nitrile-based solvents is up to ~5 V, which can cover the operating voltage window of the existing mainstream battery cathodes. Furthermore, they present high dielectric constants, low viscosities, and good dissociation effects. Therefore, after further improving the compatibility with Li anode, nitrile electrolytes are believed to have the potential to replace the existing commercial electrolyte system.

### 3.5. Solid-State Electrolytes

Solid-state electrolytes (SSEs) have aroused great interest due to their transference number and high mechanical strength, which can physically inhibit Li dendrite growth. Outstanding antioxidation stability endows SSEs with good compatibility with high-voltage cathodes. SSEs are divided into solid-state electrolytes (ISSEs) and quasi-solid-state electrolytes (QSSEs). ISSEs without liquid include inorganic solid electrolytes (ISEs), solid polymer electrolytes (SPEs), and composite polymer electrolytes (CPEs). QSSEs contain a small number of liquid components fixed inside the solid matrix. Among ISSEs, SPEs with inhibition of transition metal ion escape from cathodes, prevention of Li dendrite growth, excellent safety, and reduction of EEI reactions can potentially realize superior electrochemical performances and safety characteristics at a high voltage [162]. However, the stability requirement for solid electrolytes against electrodes has still not been fully satisfied under high-voltage conditions. Thus, introducing advanced materials and designing efficient structures are necessary. Accordingly, some antioxidation substances (PP_12_FSI, LEP, LiPVFM, and SN) or a layered architecture can be possible solutions to enhancing stability.

**Figure 17:**
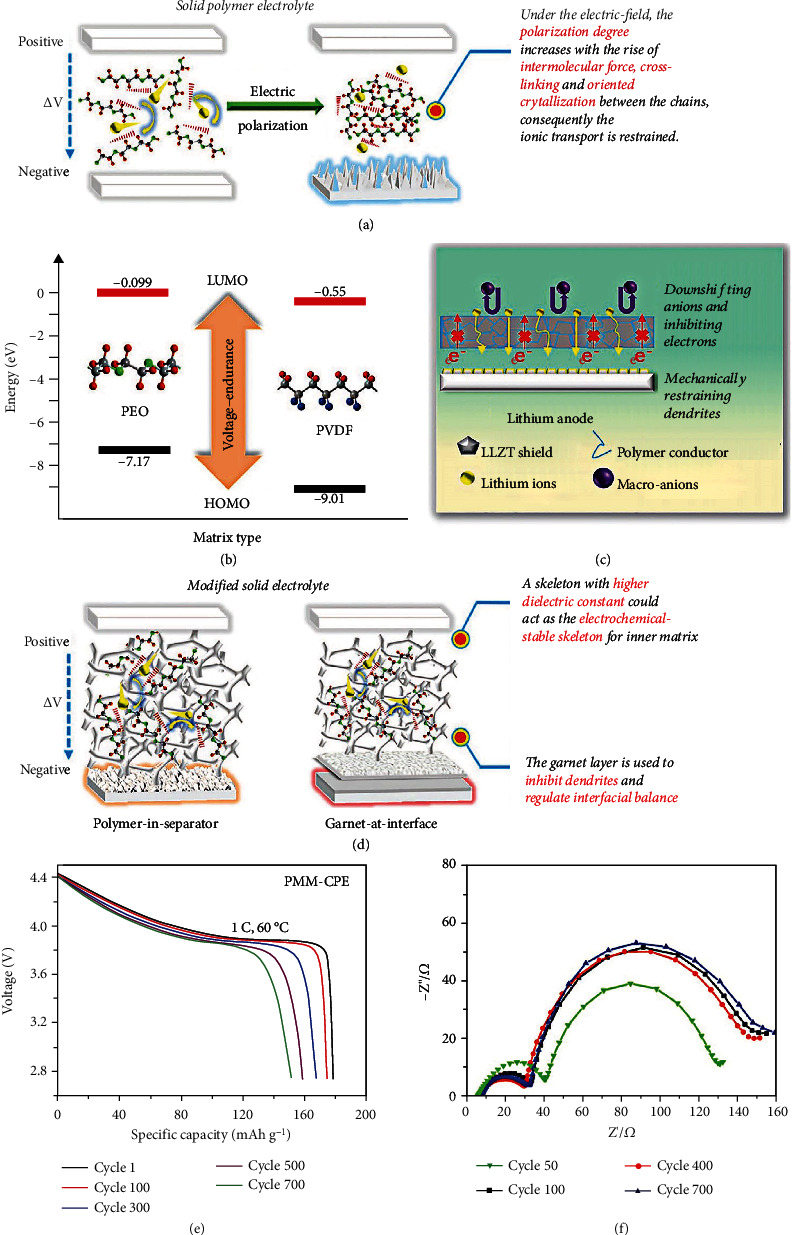
(a) Schematic illustration for the electrochemical attenuation of SPEs under the electric field. (b) Orbital energy levels of the PEO and PVDF polymer matrices. (c) The sketch for the multifunctional effects of the LLZT shield layer. (d) Schematic illustration for superiorities of modified solid electrolytes in this work. Reproduced with permission [163]. Copyright 2020, WILEY-VCH. (e) The corresponding discharge voltage curves of the 1st, 100th, 300th, 500th, and 700th cycles of the LCO||Li cell using PMM-CPE at 60°C. (f) EIS spectra of the 4.45 V-class LCO||Li cell with PMM-CPE after 50, 100, 400, and 700 cycles at 1 C (60°C). Reproduced with permission [164]. Copyright 2018, The Royal Society of Chemistry.

Sun et al. [163] combined the “garnet-at-interface” layer and “polymer-in-separator” matrix to prepare a solid electrolyte. As displayed in Figure 17(a), under an electric field, in the SPE complexed with lithium salt and polyethylene oxide (PEO), the polar groups of the polymer chain interact with active ions, which jump over the entire chain segment (amorphous region) through thermal motion. Under continuous electric field stimulation, there will be many polarization regions due to the intermolecular forces between chains, orientation crystallization, and cross-linking. Therefore, the free volume for the thermal movement of the chain is shrined, and then, the ion transmission is inhibited, destroying the stability of the cell. As displayed in Figure 17(b), PVDF owns lower HOMO energy, which means that the PVDF-based matrix has a higher high-voltage resistance and dielectric constant than PEO. Therefore, to enhance high-voltage stability, PVDF is selected as the skeleton of the polymer electrolyte. The high-ratio LLZT is introduced into the polymer to increase the shielding effect for inhibiting Li dendrite growth. Besides, this combination can act as an electronic and anionic filter. However, Li^+^ cation can migrate through the LLZT percolation and the interface between polymer and filler (Figure 17(c)). As shown in Figure 17(d), the strong electric field can be alleviated by packing the vulnerable polymer electrolyte into the porous PVDF separator, further alleviating to serve electrochemical polarization. This design helps ensure a good interface to Li metal and improves the high-voltage performance of the electrolyte. The electric potential can achieve uniform distribution in the EEIs. The embedded PVDF skeleton (high-dielectric parts) and LLZT layer jointly guarantee the uniform distribution of the electric field inside the electrolyte bulk. Regulated by the electric field, the Li^+^ ions transport through the electrolyte bulk and homogeneously deposit on the electrode surface. Accordingly, the electric potential can be well distributed, which further sustains the stable cycles of batteries. Compared with PEO and PVDF, bacterial cellulose as the substrate owns higher mechanical strength and antioxidation stability. Dong et al. [164] combined poly(methyl vinyl ether-alt-maleic anhydride) P(MVE-MA) and bacterial cellulose to form a polymer electrolyte for the HVLMBs. The LCO||Li battery using P(MVE-MA) membrane with 1 M LiODFB/PC (PMM-CPE) electrolyte exhibits excellent capacity retention of 85% of the initial capacity after 700 cycles (Figure 17(e)), remarkably superior to the LCO||Li battery using LiODFB/PC electrolyte (14%). Furthermore, the LCO||Li battery using PMM-CPE electrolyte exhibits a high and stable CE. As shown in Figure 17(f), electrochemical impedance spectroscopy (EIS) of LCO||Li batteries using the carbonate electrolyte and PMM-CPE electrolyte is tested. Two semicircles stand for the bulk electrolyte resistance and interfacial resistance, respectively. For the PMM-CPE electrolyte, interface impedance increases slowly within 700 cycles, indicating outstanding interfacial combability in the PMM-CPE electrolyte. Zheng et al. [165] developed an interpenetrating network (IPN), including a basal PEO-contained network and a linear poly(acrylonitrile) (PAN) secondary network. The chemical and architectural properties of IPN markedly enhance the oxidative stability of the electrolyte from 4.1 V to over 5.1 V by adding 2 wt.% PAN. The IPN electrolyte is promising to be applied in HVLMBs.

**Figure 18:**
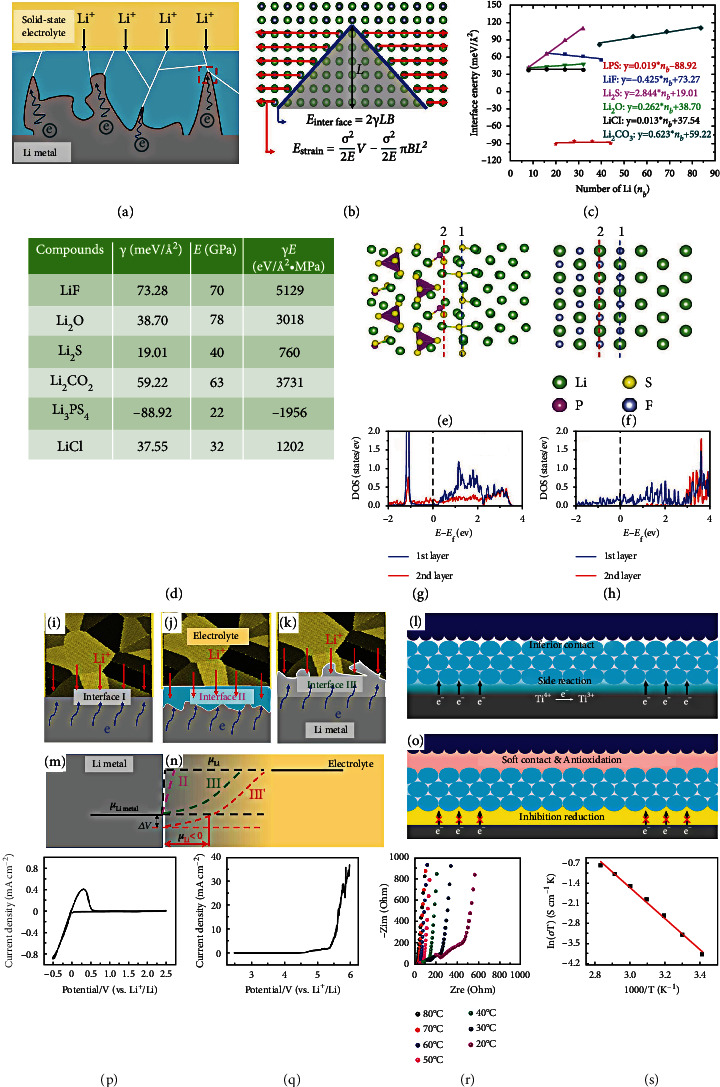
(a) Schematic illustration of the electrochemical deposition process of the Li metal anode. (b) Energy-based analysis (interfacial energy and strain energy) of Li dendrite formation. (c) The plot of the relationship between the interfacial energy for possible SEI components and the number of Li metal formula units. (d) Calculated interfacial energies *γ*, bulk modulus *E* from MP, and Li dendrite suppression ability *γE* for different interface components. DFT-optimized atomic structures of (e) LPS/Li and (f) LiF/Li interfaces and (g, h) their corresponding density of state (DOS) profiles by atomic layer with a Fermi level at 0 eV. The green, purple, yellow, and grey balls in (e, f) represent Li, P, S, and F atoms. (i) Thermodynamically stable interphase. (j) Reactive but forming an electron insulator SEI layer. (k) Reactive and forming a degradation layer with high electron conductivity. (m) Li potentials between the Li metal and the SSEs in the above three interphase types. (n) The difference between the green dash line (III) and the red dash (III′) is that the red dash line (III′) includes the overpotentials during the Li plating process. Reproduced with permission [166]. Copyright 2018, American Association for the Advancement of Science. Illustrations of the solid full battery with (l) pristine LATP and (o) DPCE. (p) Cyclic voltammetry and (q) LSV curves of the DPCE at 60°C. (r) EIS analysis and (s) Arrhenius linear fitting plots of the DPCE at various temperatures. Reproduced with permission [167]. Copyright 2019, American Chemical Society.

Fan et al. [166] reported a LiF-rich SEI layer formation to inhibit the generation of Li dendrites. The LiF-rich SEI layer owns low resistance, which is because (i) the in situ-formed thin interface is closely contacted with both Li_3_PS_4_ (LPS) and Li; (ii) the lower energy barrier for Li^+^ surface diffusion on LiF (0.17 eV for LiF and 0.23 eV for Li_2_CO_3_) accelerates the Li^+^ transfer along the LiF surface rather than the dendritic plating. Theoretical calculations based on DFT are conducted to understand the suppression mechanisms of the Li dendrite of the LiF-rich SEI layer in the SSEs. As shown in Figure 18(a), Li dendrites must pass through the passivation layer along the grain boundaries first, where strain energy remains high. According to the energy analysis, the interface energy increases due to the newly formed interface at the SEI layer. The strain energy is released with the generation of Li dendrites (Figure 18(b)). As the length of Li dendrite increases, the total energy increases and reaches the top corresponding to the fatal dendrite length Lc, followed by a slow drop. As displayed in Figure 18(c), the relationship between the Li formula number and the interfacial energies of various SEI constituents is tested. The intercept of the fitted line represents the interface energy. As displayed in Figure 18(d), Li_3_PS_4_ exhibits low interface energy of -88.92 meV/Å^2^, suggesting an unstable interface between the Li anode and Li_3_PS_4_. At the interface, the cracks are generated because the serve reaction between the Li anode and the Li_3_PS_4_ causes the Li_3_PS_4_ reconstruction with the Li-P and Li-S band generation derived from the optimized structure (Figures 18(e) and 18(f)). However, other SEI components, including LiF, Li_2_CO_3_, Li_2_O, LiCl, and Li_2_S, exhibit positive interfacial energy. Furthermore, LiF owns the largest interfacial energy of 73.28 meV/Å^2^, demonstrating its ultrahigh dendrite inhibition capacity. The interface energy multiplied by the bulk modulus is used to evaluate the dendrite inhibition ability, as shown in Figure 18(d). LiF exhibits excellent interface energy and an outstanding bulk modulus (70 GPa). As shown in Figures 18(g) and 18(h), the energy barrier of the electron tunnelling from Li to LiF or LPS is calculated. The barrier of electron tunnelling for Li_3_PS_4_ is very low, approximately zero, revealing the incapability of Li_3_PS_4_ to suppress electron tunnelling. In contrast, the barrier of electron tunnelling for LiF is 2 eV, indicating that LiF can effectively prevent electron tunnelling. The remarkable electronic shielding effect can effectively alleviate side effects between the Li metal anode and the Li_3_PS_4_. As shown in Figures 18(i)–18(k), 18(m), and 18(n), three interphases can be generated at the anode-electrolyte interface: (i) inherently stable interphase, in which electrolyte and Li metal are thermodynamically stable; (ii) regular SEI, which owns very low electronic conductivity but high Li^+^ conductivity; and (iii) electronic conducting interphase with an ultrahigh electronic conducting ability. For the first type of SSE, since Li cannot wet the SSE, the potential at the interface suddenly declines from the SSE to Li metal, resulting in higher interfacial resistance (Figures 18(m) and 18(n)). Regarding the second category, Li metal can associate with LiPON to generate SEI with high-ratio Li_2_O, which effectively prevents electron tunnelling. The third kind of SSE comprises Li_3*x*_La_2/3−*x*_TiO_3_ (LLTO), sulfide, and oxide electrolytes owing to the potential reduction of SSE by Li anode when the potential is below zero in the Li plating process to form an electronic conductive substance or an electronic semiconductor substance. Except for the strategy of *in situ* forming the LiF-rich SEI, the introduction to CPEs at the opposite position of ISE can also effectively stabilize Li metal anode and high-voltage cathode. Liang et al. [167] designed an SSE by coating PAN and PEO on both sides of Li_1.4_Al_0.4_Ti_1.6_(PO_4_)_3_ (LATP), respectively, to meet different interface requirements. It is essential to offer tailored solutions to outstanding stability with Li metal and the infiltration of high-voltage cathode assembled in SSE (Figure 18(l)). An SSE electrolyte with notable improvement is achieved by adding PEO and PAN on both sides of LATP (Figure 18(o)). As shown in Figures 18(p) and 18(q), the disparate-polymer protected ceramic electrolyte (DPCE) exhibits a high oxidation potential of 4.5 V and highly reversible Li plating/stripping. In addition, sizeable ionic conductivity (6.26 × 10^−4^ S cm^−1^) and low activation energy (0.46 eV) are achieved in LATP at 60°C (Figures 18(r) and 18(s)). To further explain the interfacial stability related to the ion manipulation triggered by the LATP ceramic in DPCE, COMSOL Multiphysics is conducted to explore the ion layer with high-*t*_Li_^+^. With abundant free anions, the charging process leaves a large concentration gradient field or space-charge layer at the electrolyte/anode interface and hence increases the Li^+^ transport barrier and polarization, which induces dendrite formation, decomposition of polymer electrolyte, and inferior cycling stability.  Table 9 shows the cycling performance of the HVLMBs with different SSEs.

#### 3.5.1. Section Summary

In this section, recent development in the SSEs has been discussed, including LiTFSI/PC/PMM-CPE, LiFSI/PVH/PP_12_FSI, P(CUMA-NPF_6_)-GPE, LiTFSI/PVDF-HFP/LLZO, and LATP/PEO/PAN systems. The ion transfer mechanisms in the SSEs are entirely different from traditional electrolytes. For crystalline solid electrolytes, ion transfer dramatically depends on the defects in the crystal structure, including electron defects, volume defects, planar defects, line defects, and point defects. In the middle of these defects, point defects undertake a pivotal part in Li^+^ transfer mechanisms. The most typical defects are the Schottky point defects, where the cation vacancies are accompanied by anion vacancies, and Frenkel point defects, where cation interstitials accompany anion vacancies. In particular, the ion transfer mechanisms occurring on point defects include (i) nondefect mechanisms, which are divided into collective mechanism, interstitial-substitutional exchange mechanism, and interstitial mechanism, and (ii) defect mechanisms, which are divided into vacancy mechanism and simple vacancy mechanism. For SPEs, Li^+^ transfer generally happens in the amorphous phase. The free-volume model and additional theoretical methods are employed to simulate the Li^+^ conductivity mechanism in the amorphous phase. In the model, the Li cations distribute in the appropriate coordination locations of the polymer section chain. These chains experience topical segmental movement with a quasiliquid action. The Li cation leaps from one coordination position to another through free sites on the same chain segment or two different chain segments driven by the electric field force. Overall, the solid electrolytes based on the combination between inorganic materials and polymers, the association of different polymers, and the QSSEs with a small amount of liquid electrolyte have improved the electrochemical stability under high voltage. In addition to enhancing the high-voltage stability of the SSEs, the interface between electrode and SSE also needs to be further strengthened. Specifically, the issues including large interfacial impedance, element interdiffusion, lithium dendrite growth, side reactions aroused by high-voltage decomposition, and space charge layer especially at the electrolyte/cathode interface are being further concerned. It is believed that developing strategies such as layered architecture and *in situ* gelation can solve these problems to promote the application of SSEs in HVLMBs.

## 4. Conclusions and Prospects

The ever-increasing demand for high-energy-density energy storage devices has put HVLMBs on the agenda in recent years, which has also sentenced conventional electrolytes with narrow electrochemical windows to death owing to their failure of compromising the catalytic nature of high-voltage cathodes and the infamous reactivity of the Li metal anode. A host of strategies are developed to overcome the aforementioned challenges, mainly classified into two categories: conventional electrolyte modification and novel electrolyte design. The important properties of representative electrolytes based on conventional electrolyte modification and novel electrolyte design are displayed in Table 10.

### 4.1. Advantages and Disadvantages of Various Strategies

**Figure 19:**
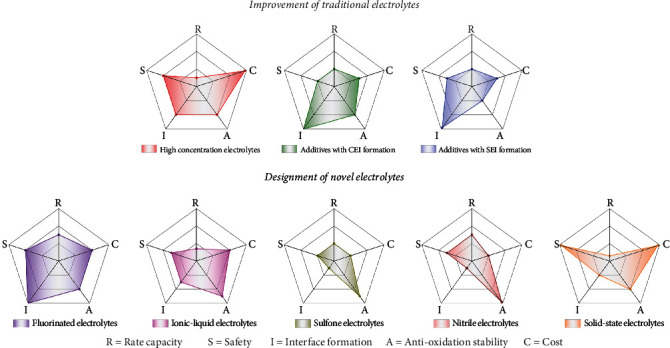
The strategies to improve the rate capacity, cost, antioxidation stability, interface formation, and safety.

The advantages and disadvantages of the improvement strategies for traditional electrolytes and the novel electrolytes are shown in Figure 19 and Table 11.

As breakthrough progress, the success of the HCEs can be attributed to the improved cation aggregation state (solvent-cation and anion-cation coordination conditions), which provides technical advantages including wide voltage window, decreased combustion, and advanced interface chemistry over commercial dilute electrolytes. Nevertheless, heavy use of expensive lithium salts and significantly increased viscosity bring cost concerns, low rate capacity, and inferior compatibility with thick cathodes for industrial applications. Based on the above issues, LHCEs have been developed to combine the advantages between HCEs and conventional concentration electrolytes. The selection of the diluents that do not participate in the Li^+^ solvation structure is the key for LHCEs. Future research should pay attention to the price and flammability of the diluents. To construct the LHCEs, the blind use of expensive height fluorinated solvents and highly volatile ether solvents is not applicable to commercial applications. Cost-effective flame-retardant diluent with low HOMO energy level can be employed to improve comprehensive performances of the LHCEs for practical applications. For example, the integration of phosphate diluent and HCE aqueous electrolytes gives the opportunity to achieve cost-effective high-security electrolytes. Moreover, it is sagacious to develop novel diluents by grafting polar functional groups in annular organic matters. For the design principles of solvents, carbonate-based, sulfone-based, and nitrile-based electrolyte solvents with strong reactivity and poor film-formation ability for Li metal are difficult to apply in HVLMBs. The formation of the anion-derived SEI layer induced in the HCEs can significantly enhance the compatibility between the electrolyte and the Li anode. Moreover, the reduced free solvent number in the HCE can improve the high-voltage stability of ether-based solvents, which promotes their applications in HVLMBs. As for the design principles for lithium salts, great success has been achieved for HCEs, primarily attributed to the LiF-rich SEI layer as derived from the anion. The big anion size in LiFSI and LiTFSI decreases the Coulombic forces between cations and anions in the electrolyte, ensuring high solubility in the solvents. In HCEs, the interaction between Li^+^ and anions significantly affects the interface formation and electrode passivation processes. Due to the uniform and dense LiF-rich EEIs as induced by TFSI^−^ and FSI^−^, the interface can remarkably inhibit Li dendrite growth and tolerate the significant volume change of high-voltage/capacity electrodes. The design principles for LHCE are mainly centered on diluents. The selection principles of the diluents mainly include (i) weakening Li^+^ binding energy without participating in the Li^+^ solvent shell to achieve localized high concentration environment, (ii) lowering the viscosity by reducing the HCE viscosity and increasing the wettability of the electrolyte for electrodes and separators, (iii) lowering the LUMO energy level and facilitating the SEI layer formation, (iv) lowering the cost for promoting large-scale applications, (v) lowering the combustibility and enhancing battery safety, and (vi) weakening toxicity and reducing pollution for battery recycling. If the existing problems can be fundamentally settled, LHCEs will enable HVLMBs with larger power and energy densities.

As to additives for EEI formation, the addition of a tiny dose of adventive molecules is considered one of the simplest and economical ways to construct strong EEIs, which is closely correlated with the electrochemical stability of electrodes and electrolytes. The additives chosen for Li anode and high-voltage cathode should own higher reduction potential and lower oxidation potential compared with electrolytes. Future research can focus on the evaluation of the Al foil corrosion induced by additives and the development of additives with suppression function of lattice oxygen escaping. In the laboratory coin-cell evaluation system, the negative impact of the current collector corrosion is weakened. However, for pouch and cylindrical cells, the Al foil corrosion causes the comprehensive deterioration of electrochemical performances. In addition, the foreign additive under a high voltage is extremely easy to corrode the Al foil. Hence, for the electrolytes after additive introduction, Al foil corrosion should be carefully evaluated. The cathode with oxygen valence activities is often accompanied by irreversible oxygen loss during charge and discharge, resulting in structural degradation of cathode materials. It is suggested that Quantum Chemistry Calculations including G4MP2 calculation and SMD solvation model are employed to filter the additive firming lattice oxygen. Moreover, an excellent additive should meet the following design principles: (i) it should sacrificially decompose before the electrolyte solvent components to passivate the high-voltage cathode and Li metal anode, (ii) the gas generation can be effectively inhibited, (iii) the generated SEI and CEI layers should be compact and thin with low electrochemical impedance, and (iv) the generated SEI and CEI layers should tolerate the extreme potential and the significant volume fluctuation of the electrodes.

For the fluorinated electrolytes, outstanding antioxidation stability makes them promising candidates for the HVLMB electrolyte. In addition, the fluorinated solvent owns high thermal stability and even nonflammability. Nevertheless, highly fluorinated solvents with high purity tend to be expensive, which limits their further development. Therefore, it is suggested that instead of pursuing highly fluorinated solvents, the fluorinated group functions in different positions of the molecules are analyzed in depth to guide the molecular structure design for lowly fluorinated solvents. For example, the position of the fluorinated group in the molecular structure has a profound effect on the capabilities of LiF formation and Li^+^ coordination. Moreover, comprehension of the influence of the fluorinated electrolytes on the HVLMB performances requires insights into two correlations: one between electronic distribution and molecular structure in the fluorinated solvent and the other between nonfluorinated solvent and fluorinated solvent. At last, a safe fluorinated-based electrolyte should follow design principles: (i) high thermal stability, which ensures that the physicochemical properties of the electrolyte can be well maintained at a rapidly increased temperature; (ii) low flammability and even nonflammability, which can significantly improve battery safety; (iii) low flash point, which is especially crucial for the drastic decrease in the flash point with an increasing degree of fluorination; and (iv) high boiling point, which prevents the internal air pressure of the battery from being too large at a high temperature.

For ionic-liquid, sulfone, and nitrile electrolytes, wide electrochemical window and excellent cathode affinity make them promising candidates for the new generation of HVLMB electrolytes. Unfortunately, the inferior compatibility with Li metal prevents their further development. Cheap film-formation solvents can be introduced to stabilize the Li anode, but the distribution of the mixed solvents is random inside the HVLMBs. It is suggested that DFT calculations be employed in achieving selective distribution of each solvent in the mixed electrolyte. For example, nitrile-based compounds are screened to obtain solvents with large absorption energy to the high-voltage cathode but low absorption energy to the Li anode. Film-formation compounds are also screened to obtain the solvents with large absorption energy to the Li anode but low absorption energy to the high-voltage cathode. The screened solvents in the mixed electrolyte wet the two electrodes, which facilitates bielectrode affinity. It is noted that the cost of ionic liquid is obviously higher than those of sulfone and nitrile, so the introduced proportion of cheap doped solvents should be higher to control the cost. The excellent antioxidation stability of the sulfones can be attributed to the powerful intrinsic stability of the sulfone group against oxidation at an extreme potential. On the contrary, the antioxidation stability of nitrile-based electrolytes is attributed to the prioritized decomposition on the surface of the high-voltage cathode, generating an effective protective layer of -C≡N/transition metal complexes to avoid the intimate contact between other species and catalytic surfaces of the cathode. In addition, similar to carbonate functional groups in alkyl carbonate molecules with large dielectric constants and dipolar moments, the terminal -C≡N functional groups are excellent nucleophilic sites for coordinating Li^+^.

SSEs have received abundant attention owing to their excellent characteristics of robust mechanical strength, strong antioxidation capability, no risk of leakage, and nonflammability. Nevertheless, SSEs cannot support HVLMBs to own satisfactory electrochemical performances due to the large interfacial resistance, mechanical collapse, and interfacial side reaction. Future research can focus on the failure mechanism of the HVLMBs using SSEs. Battery failure associated with EEI kinetic process is correlated with Li^+^/e^−^ transport and combination, chemical/electrochemical reactions, and pressure change. It is suggested that the complex process that is difficult to touch experimentally should be simulated through *ab initio* molecular dynamics, where electron/ion transport and combination are imitated with the PAW way, all molecular evolutions are caught in the NVT ensemble using a Nosé-Hoover thermostat, and the free energy of the system is obtained by integrating the position-dependent mean constraint forces. *In situ* gelation for solid-state polymer electrolyte owns an in-built interface with rapid ion transport, which is regarded as one of the most promising SSE technologies. Future research on this technology should be centered on simplifying the polymerization conditions and enhancing the antioxidation stability after polymerization. In addition, future research focusing on the design principles should also be highlighted, such as interfacial characterizations for guiding the design of the interfacial chemical reaction kinetics and the design of the interfacial conservation films with antioxidation and reduction inhibition to widen the electrochemical window for HVLMBs.

### 4.2. Future Directions

**Figure 20:**
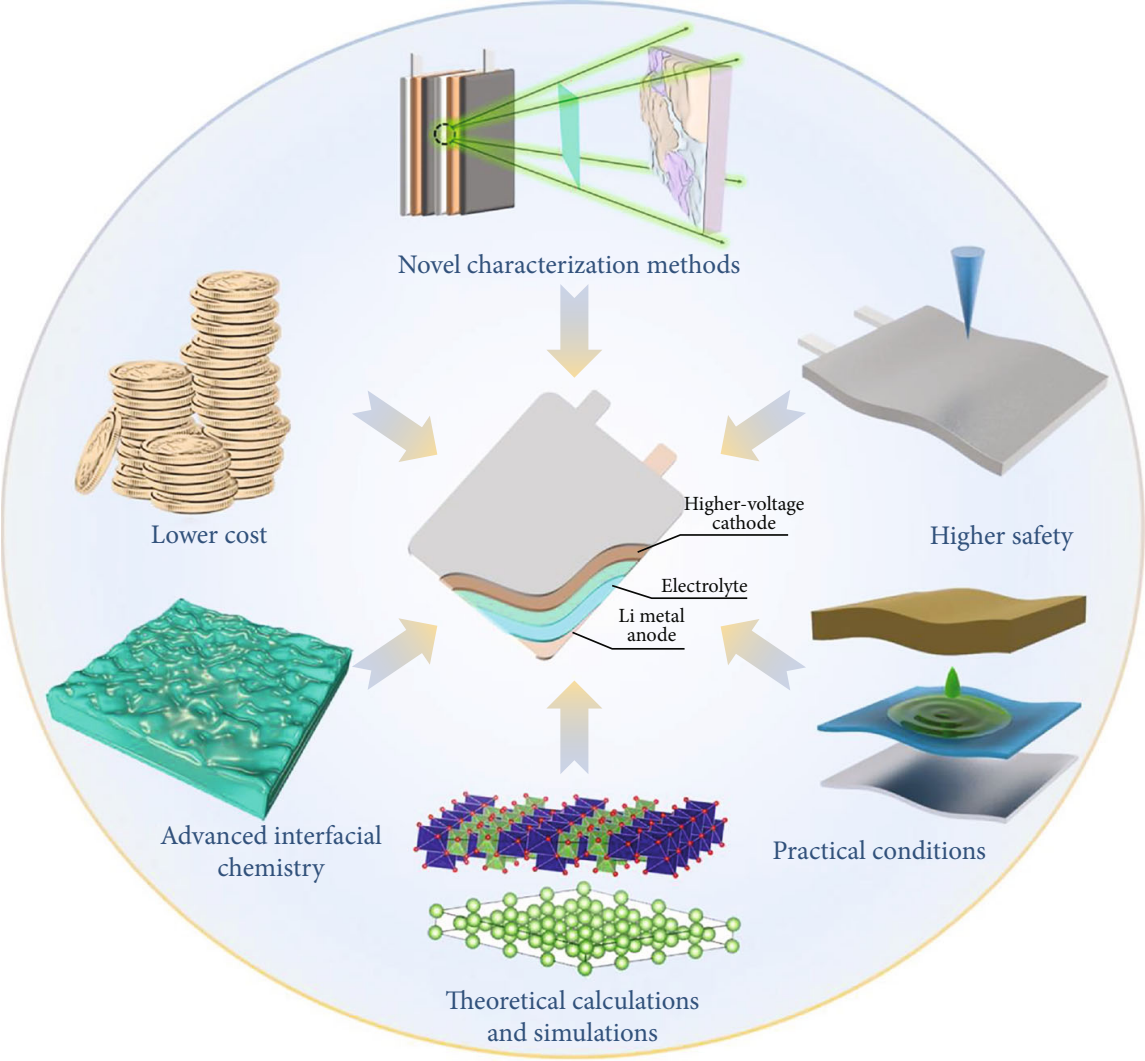
Several critical issues of electrolytes for HVLMBs.

Although valuable progress has been made, several critical issues remain to be resolved for the electrolyte of the practical HVLMBs (Figure 20):

1. *Higher Safety*. The catalytic nature of high-voltage cathodes and the infamous reactivity of Li anode make the inherently flammable traditional liquid electrolyte more prone to dangerous accidents. Fluorine introduction is expected to be an effective solution to alleviate the inflammability of the conventional electrolyte. In addition, the toxicity of the electrolyte solvent can also be reduced after fluorination. The applications of ionic-liquid and nitrile solvents are also a feasible choice regarding their thermostability and flame retardancy. Of course, in terms of safety, SSEs far exceed liquid electrolyte. It is noteworthy that the conclusions drawn from a simple ignition experiment on the electrolyte are unreliable. The so-called nonflammable electrolyte is still dangerous in actual operation. More stringent inspections must be conducted, such as a nail-penetration test. Cost-effective flame-retardant diluents can be employed to improve the comprehensive performances of the LHCEs for practical applications. For example, the integration of phosphate diluents and HCE aqueous electrolytes helps achieve cost-effective high-security electrolytes. Moreover, it is sagacious to obtain novel diluents by grafting polar functional groups (-COOH, Ar-OH, -OH, -NHCO-CH_3_, -NH_2_, -SH, -CHO, and CH_3_-N-CH_3_) in annular organic matters (alicyclic compounds, aromatic compounds, and heterocyclic compounds).
2. *Lower Cost*. In traditional LIBs, the cost of electrolyte accounts for ~20%, which will be further increased in the HVLMBs due to the greater demand for additives with complex functions, large amounts of lithium salts, and novel solvents. Therefore, it is vital to reduce the electrolyte cost. More detailly, lithium salt has the highest cost proportion among the compositions of the electrolyte. Although the HCEs can significantly expand the electrochemical window of traditional electrolytes, the cost is too high for its large proportion of lithium salts. This issue can be effectively solved by the LHCEs. Contrary to the HCEs, low concentration (<1 M) electrolytes (LCEs) pursue high performance while maintaining low cost. Herein, it is suggested that strong reduction additives (CuBr_2_, BeBr_2_, MgBr_2_, CaBr_2_, LiBr, NaBr, and KBr) are introduced to reactivate dead Li and release active Li^+^ for reuse in the LCEs by supplementing Li^+^ and suppressing dead Li growth. Furthermore, the systematic research on the collaborative relationship of solvents, salts, and ionic aggregate structure undoubtedly facilitates the breakthrough of the LCE field.
3. *Practical Conditions*. It is noted that most of these electrolytes are assessed in coin-cell systems (Tables 2–9) with thin high-voltage cathodes (<5 mg cm^−2^), flooded electrolytes (*E*/*A* > 10 g Ah^−1^), and thick lithium metal anodes (>200 *μ*m), whereas evaluations of them in commercial pouch batteries display unsatisfactory energy density and inferior cycle life. Herein, it is suggested that the novel electrolyte should be tested with high-loading cathodes (≥20 mg cm^−2^), lean electrolytes (*E*/*A* ≤ 3 g Ah^−1^), and thin Li metal anodes (≤20 *μ*m). High-loading cathode and lean electrolyte conditions require low-viscosity and highly wetting cosolvents, such as 1-methylpentyl acetate, 1,4-dichlorobenzene, 4-methyl-2-pentanone, and 2-heptanone. Moreover, the CE test of Li/Cu batteries is one of the most widely used methods of assessing Li^+^ stripping/plating reversibility in the interface between the electrolyte and the Li anode. It is suggested that the thickness of the Li foil is 50 *μ*m and the amount of the electrolyte is 15 *μ*L. During cycling, 4-5 mAh cm^−2^ of lithium (~20-25 *μ*m) plated on the Cu current collector provides a suitable areal capacity to be matched with high-loading cathodes. The CEs of the Li/Cu cells under this stringent condition are close to the measured CEs in the HVLMBs with thin Li anodes.
4. *Advanced Interfacial Chemistry*. Despite a small part in the HVLMB system, SEI and CEI play a vital part in preventing the further reaction between electrolyte and electrodes to alleviate the transition metal dissolution and the growth of Li dendrites. In addition, the EEIs affect the lithiation and delithiation of Li^+^ on the electrode surface. Electrochemical reactions at the interphases are triggered during charging/discharging with the generation of Li anode-side SEI and the CEI counterpart due to the initial reduction and oxidation of electrolyte. Therefore, the electrolyte directly determines the composition of SEI and CEI. The ideal interphase is characterized by the uniform and robust inorganic-organic composite layer. Take the fluorinated interphases generated in the fluorinated electrolyte as an example, its high interfacial energy (73.28 meV/Å^2^) and antioxidation stability (6.4 V *vs.* Li/Li^+^) can facilitate Li^+^ transport along with the interface and prevent continuous side reactions between electrolyte and electrode. Similarly, S- and N-rich SEI/CEI have also been proven to have multiple functions. Herein, future research for interfacial chemistry can focus on the following two directions: interfacial layered construction and quantitative combination ratio of beneficial substances at the interface.
5. *Theoretical Calculations and Simulations*. Theoretical research assisted by artificial intelligence, large data sets, machine learning, and supercomputing power will help to quickly screen and foresee the next-generation electrolyte systems that support the HVLMBs. Furthermore, theoretical research will assist in an overall grasp of what happened to the EEIs in the HVLMBs at the molecular scale, which is the area that the experiment cannot touch. However, the predictive ability of theoretical calculations is rarely used in the HVLMBs at present. This field will undoubtedly promote the development of the HVLMB electrolyte. For example, Quantum Chemistry Calculations including G4MP2 calculations and SMD solvation models are employed to filter the additive firming lattice oxygen. Furthermore, by using machine learning and adsorption energy calculations, the film-formation solvent (VC, FEC, vinyl ethylenecarbonate (VEC), ethylene sulfite (ES), vinylethylene sulfite (VES), ethylene sulfate (DTD), 4-propyl- [1,3,2]dioxathiolane-2,2-dioxide (PDTD), diphenyl disulfide (DPDS), tris(2,2,2-trifluoroethyl) borate (TTFEB), and ionanofluid (InF)) and antioxidation solvent (ionic-liquid, sulfone, and nitrile) in the mixed electrolyte can individually wet the two electrodes, which facilitates bielectrode affinity. Besides, the complex interface evolution process should be simulated by *ab initio* molecular dynamics, where electron/ion transport and combination are imitated by the PAW way, all molecular evolution is caught in the NVT ensemble using a Nosé-Hoover thermostat, and the free energy of the system can be obtained by integrating the position-dependent mean constraint forces.
6. *Novel Characterization Methods*. Accurate characterizations of the electrolyte and interphases are still very difficult nowadays, which is ascribed to their low content, sensitivity to electron radiation and X-ray, and reactivity to water and oxygen. *In situ* characterization technology can collect signals from electrolyte and interphases during battery operation, which is undoubted of paramount significance for the accurate and deep research of the internal changes of the HVLMBs. The application of cryoelectron microscopy in the HVLMBs is groundbreaking, which minimizes the influence of oxygen and water during the test. Current *in situ* characterization technologies are mainly based on coin-cell configuration. It is suggested that the progressive characterization should be further integrated with pouch and cylindrical cells.

## Figures and Tables

**Table 1:** The HOMO and LUMO values corresponding to the different substances in Figure 3.

Substance	HOMO (eV)	LUMO (eV)
HFE	-8.42	-0.09
FEC	-7.46	-0.87
AN	-7.93	-0.22
BN	-7.76	-0.19
TMB	-6.27	0.71
TEB	-6.39	1.14
TTS	-6.54	-0.18
MSM	-6.99	0.17
TMS	-6.72	0.11
PEO	-5.85	0.62
PVDF	-7.58	-0.03
EMIm^+^	-9.94	-3.23
FSI^−^	-8.33	-2.87
LiPF_6_	-8.31	-0.67
LiFSI	-7.73	-1.70
LiTFSI	-7.82	-1.51
LiNO_3_	-6.87	-0.81
Mg(NO_3_)_2_	-6.95	-0.79
Mg(TFSI)_2_	-8.19	-0.53

**Table 2:** The cycling performance of the HVLMBs with different HCEs and LHCEs.

Rate (C)	Cathode	Electrolyte	Category	Lithium salt concentration (mol/L)	Solvent volume ratio (Vol : Vol)	Charging cut-off voltage (V)	Cathode loading (mg cm^−2^)	Lithium metal thickness (*μ*m)	Electrolyte amount (*μ*L)	Initial capacity (mAh g^−1^)	Cycle number	Retention (%)	Decay per cycle (%)	Ref. No.
0.20	LNMO	LiFSA/DMC	HCE	5.5	—	5.20	0.7-2.0	>200	160	~128	100	~95.0	~0.05	[58]
0.50	NMC523	LiTFSI/DMC/[C_2_mpyr][FSI]	HCE	3.5	1 : 1	4.50	4.0	>200	Not mentioned	~160	100	~95.0	~0.05	[67]
0.20	NMC622	LiFSI/EC/DMC	HCE	10.0	1 : 1	4.60	13.0	>200	Not mentioned	~225	100	~86.0	~0.14	[57]
0.50	LNMO	LiTFSI/LiDFOB/FEC/DMC	HCE	4.0/0.5	3 : 7	4.90	8.3	>200	150	~120	100	~87.8	~0.13	[68]
0.20	NMC811	LiFSI/PC/FEC	HCE	5.0	93 : 7	4.60	17.0	35	200	~228	60	~96.0	~0.07	[69]
0.33	NMC811	LiFSI/DME/TTE	LHCE	1.7	2 : 7	4.40	15.3	50	12	~200	155	~80.0	~0.13	[60]
1.00	NMC622	LiFSI/TEP/BTFE	LHCE	1.2	1 : 2	4.40	10.0	450	100	~185	600	~97.0	~0.01	[63]
1.00	NCM811	LiPF_6_/EC/DMC/Zeo	LHCE	1.0	1 : 1 : 1	4.40	5.0	400	50	~187	1030	~83.2	~0.02	[70]
1.00	NCM87	LiDFOB/LiNO_3_/TMP/TTE	LHCE	1.0/0.1	3 : 10	4.50	4.5	580	75	~204	200	~90.0	~0.05	[71]
0.50	NCM622	LiDFOB/DMMP/TTE	LHCE	1.1	1 : 1	4.50	4.0	500	80	~180	100	~98.0	~0.02	[72]
0.25	LCO	LiDFOB/LiBF_4_/DEC/FEC/FB	LHCE	0.3/0.2	3.5 : 1.5 : 5.0	4.60	20.4	50	17	~200	120	~85.6	~0.12	[62]
0.50	LCO	LiFSI/DME/TTE	LHCE	1.0	2 : 8	4.40	12.7	20	Not mentioned	~190	200	~85.0	~0.03	[73]
0.33	NCM622	LiFSI/TEP/BTFE	LHCE	1.2	1 : 2	4.40	21.4	50	20	~170	200	~86.0	~0.07	[74]
0.30	NCM622	LiFSI/DME/TTE	LHCE	1.5	1 : 4	4.40	20.0	20	16	~175	600	~71.0	~0.05	[75]
0.50	NCM811	LiPF_6_/LiBF_4_/LiNO_3_/FEC/EMC	LHCE	1.0/0.2/0.3	3 : 7	4.40	12.8	45	40	~221	250	~80.3	~0.08	[76]
0.50	NCM811	LiPF_6_/FEC/FDEC	LHCE	1.0	3 : 7	4.80	8.0-8.5	50	30	~235	100	~85.7	~0.14	[77]

^∗^Abbreviation: [C_2_mpyr][FSI] = N-ethyl-N-methyl-pyrrolidinium bis(fluorosulfonyl)imide; FEC = fluoroethylene carbonate; PC = propylene carbonate; TTE = 1,1,2,2-tetrafluoroethyl-2,2,3,3-tetrafluoropropyl ether; TEP = triethyl phosphate; BTFE = bis(2,2,2-trifluoroethyl) ether; Zeo = zeolite; DMMP = dimethyl methyl phosphonate; DEC = diethyl carbonate; FB = fluorobenzene; EMC = ethyl methyl carbonate; FDEC = bis(2,2,2-trifluoroethyl) carbonate.

**Table 3:** The cycling performance of the HVLMBs with CEI formation using different additives.

Rate (C)	Cathode	CEI additive	Electrolyte	Charging cut-off voltage (V)	Cathode loading (mg cm^−2^)	Lithium metal thickness (*μ*m)	Electrolyte amount (*μ*L)	Initial capacity (mAh g^−1^)	Cycle number	Retention (%)	Decay per cycle (%)	Ref. No.
0.50	LRO	TMB	LiPF_6_/EC/EMC/DEC/TMB	4.80	1.5	>200	200	~220	375	~77.4	~0.060	[84]
0.50	LNMO	TMSPO	LiPF_6_/EC/EMC/DEC/TMSPO	4.90	4.0	400	100	~130	200	~94.6	~0.027	[85]
2.00	NMC76	LiPF_6_	LiTFSI/LiBOB/LiPF_6_/EC/EMC	4.50	4.0	450	100	~175	1000	~80.6	~0.019	[86]
1.00	NMC333	Mg(TFSI)_2_	LiPF_6_/EC/EMC/Mg(TFSI)_2_	4.60	7.0	>200	200	~153	47	~96.1	~0.083	[87]
1.00	LCO	TPFPB/LiNO_3_	LiPF_6_/FEC/EMC/TPFPB/LiNO_3_	4.60	10.0	46	11	~203	140	~80.0	~0.143	[88]
1.00	Li_1.2_Ni_0.2_Mn_0.6_O_2_	4-ABA	LiPF_6_/EC/DEC/4-ABA	4.80	1.3	>200	Not mentioned	~175	100	~94.4	~0.056	[89]
1.00	LCO	TAEC	LiPF_6_/EC/DEC/TAEC	4.50	4.0	>200	Not mentioned	~190	100	~85.1	~0.149	[90]
1.00	NMC333	TEB	LiPF_6_/EC/EMC/DEC/TEB	4.50	3.7	400	100	~164	150	~99.8	~0.001	[91]
1.00	LCP	TTSPi/FDEC	LiPF_6_/EC/DMC/TTSPi/FDEC	4.95	4.1	>200	120	~80	80	~85.0	~0.002	[92]
1.00	LCO	DEPP	LiPF_6_/EC/DMC/DEPP	4.50	1.7-2.0	>200	Not mentioned	~170	100	~69.5	~0.305	[93]
0.50	LCO	AIP	LiPF_6_/EC/EMC/AIP	4.60	5.0	600	100	~220	200	~78.1	~0.110	[94]

^∗^Abbreviation: TPFPB = tris(pentafluorophenyl)borane; TMB = trimethyl borate; TMSPO = 4-(trimethylsiloxy)-3-pentene-2-one; Mg(TFSI)_2_ = magnesium bis(trifluoromethanesulfonyl)imide; 4-ABA = 4-aminobenzoic acid; TAEC = ethyl 5-amino-4-cyano-3-(2-ethoxycarbonylmethyl) thiophene-2-carboxylate; TEB = triethylborate; TTSPi = tris(trimethylsilyl) phosphite; DEPP = diethyl phenylphosphonite; AIP = aluminum isopropoxide.

**Table 4:** The cycling performance of the HVLMBs with SEI formation using different additives.

Rate (C)	Cathode	SEI additive	Electrolyte	Charging cut-off voltage (V)	Cathode loading (mg cm^−2^)	Lithium metal thickness (*μ*m)	Electrolyte amount (*μ*L)	Initial capacity (mAh g^−1^)	Cycle number	Retention (%)	Decay per cycle (%)	Ref. No.
2.00	NCM523	CTAC	LiPF_6_/PC/EC/DEC/CTAC	4.50	1.2	600	30	~138	300	~92.2	~0.026	[100]
1.00	LNMO	TTS	LiPF_6_/EC/EMC/DEC/TTS	4.90	1.1	300	50	~110	500	~92.0	~0.016	[101]
1.00	LNMO	HFiP	LiPF_6_/EC/EMC/HFiP	4.95	8.0	250	50	~120	150	~92.0	~0.053	[102]
0.33	NCM811	TFEO	LiTFSI/FEC/TFEO	4.40	5.5	250	75	~210	300	~80.0	~0.067	[97]
0.10	NCM523	FEP	LiPF_6_/EC/EMC/FEP	4.60	9.5	500	Not mentioned	~197	50	~78.0	~0.440	[103]
1.00	LLRO	LiHFDF	LiPF_6_/EC/DEC/LiHFDF	4.60	3.5-4.0	400	50	~211	300	~75.4	~0.082	[104]
0.50	NCM811	HFPTF	LiPF_6_/EC/DMC/MSIPE/HFPTF	4.50	5.0	>200	30	~190	150	~68.1	~0.213	[105]
1.00	LNMO	EB-COF:NO_3_	LiPF_6_/EC/DEC/EB-COF:NO_3_	4.90	14.6	50	10	~120	600	~92.0	~0.013	[106]
1.00	LNMO	4TP	LiPF_6_/EC/EMC/DEC/4TP	4.90	2.5-2.8	500	50	~125	480	~89.0	~0.023	[107]
1.00	NCM622	K^+^PFHS	LiPF_6_/EC/DMC/K^+^PFHS	4.50	3.0	>200	Not mentioned	~175	200	~77.1	~0.115	[108]
0.50	LNMO	PFPN	LiPF_6_/EC/DEC/ PFPN	4.90	11.0	>200	40	~118	100	~90.7	~0.093	[32]

^∗^Abbreviation: CTAC = hexadecyl trimethylammonium chloride; TTS = trimethoxy(3,3,3-trifluoropropyl)silane; FEP = fluorinated ethyl phosphate; LiHFDF = lithium 1,1,2,2,3,3-hexafluoropropane-1,3-disulfonimide; HFPTF = hexafluoroisopropyl trifluoromethanesulfonate; 4TP = 4-trifluoromethylphenylboronic acid; K^+^PFHS = potassium perfluorohexyl sulfonate; PFPN = ethoxy(pentafluoro)cyclotriphosphazene.

**Table 5:** The cycling performance of the HVLMBs with different fluorinated electrolytes.

Rate (C)	Cathode	Fluorinated solvent	Electrolyte	Charging cut-off voltage (V)	Cathode loading (mg cm^−2^)	Lithium metal thickness (*μ*m)	Electrolyte amount (*μ*L)	Initial capacity (mAh g^−1^)	Cycle number	Retention (%)	Decay per cycle (%)	Ref. No.
0.20	Li_1.2_Mn_0.56_Co_0.08_Ni_0.16_O_2_	2FEC/TTE	LiPF_6_/DMC/2FEC/TTE/TMSP	4.70	10.3	>200	Not mentioned	~265	100	~94.3	~0.057	[113]
0.50	NCM811	MTFP/FEC	LiPF_6_/MTFP/FEC	4.50	6.5	50	Not mentioned	~202	250	~80.0	~0.080	[114]
1.00	LCO	TTE	LiFSI/DME/TTE	4.50	13.5	450	75	~158	800	~80.0	~0.025	[115]
0.20	NCM811	FEMC/DFDEC	LiPF_6_/PC/FEMC/DFDEC	4.50	3.0	500	50	~225	100	~95.0	~0.050	[116]
1.00	LiCoMnO_4_	FEC/FDEC/HFE	LiPF_6_/FEC/FDEC/HFE	5.30	1.0	>200	Not mentioned	~123	1000	~80.0	~0.020	[117]
0.33	NCM811	TFEO	LiFSI/DME/TFEO	4.40	7.5	50	75	~185	200	~80.0	~0.100	[23]
0.20	NCM811	FTriEG	LiFSA/DEG/FTriEG	4.40	4.0	350	30	~132	100	~78.0	~0.220	[110]
0.50	NCM811	FEC/FEMC/HFE	LiPF_6_/FEC/FEMC/HFE	4.40	10.8	250	100	~195	450	~90.0	~0.022	[54]
0.50	LNMO	TFEP/FEMC	LiN(SO_2_F)_2_/TFEP/FEMC	4.90	3.0-4.0	400	Not mentioned	~125	200	~70.0	~0.150	[21]
0.20	NCM811	F5DEE	LiFSI/F5DEE	4.40	24.5	50	45	~215	200	~80.0	~0.100	[118]
0.50	NCM811	FEC/DFEC/FEMC	LiPF_6_/FEC/DFEC/FEMC	4.40	8.3	>200	30	~202	200	~90.8	~0.046	[119]
0.50	NCM811	DTDL	LiFSI/DTDL	4.30	5.0	20	40	~160	200	~84.0	~0.080	[120]
0.50	NCM811	cFTOF	LiFSI/cFTOF	4.30	5.0	20	40	~150	100	~94.0	~0.060	[121]
0.33	NCM811	TTE	LiFSI/Cl-DEE/TTE	4.60	10.0	50	75	~200	200	~88.0	~0.060	[122]

^∗^Abbreviation: MTFP = methyl 3,3,3-trifluoropionate; DFDEC = di-(2,2,2-trifluoroethyl)carbonate; FEMC = 3,3,3-fluoroethylmethyl carbonate; DFEC = difluoroethylene carbonate; DTDL = 2,2-dimethoxy-4-(trifluoromethyl)-1,3-dioxolane; cFTOF = 2-ethoxy-4-(trifluoromethyl)-1,3-dioxolane; Cl-DEE = 1,2-bis(2-chloroethoxy)-ethyl ether.

**Table 6:** The cycling performance of the HVLMBs with different ionic-liquid electrolytes.

Rate (C)	Cathode	Ionic-liquid solvent	Electrolyte	Charging cut-off voltage (V)	Cathode loading (mg cm^−2^)	Lithium metal thickness (*μ*m)	Electrolyte amount (*μ*L)	Initial capacity (mAh g^−1^)	Cycle number	Retention (%)	Decay per cycle (%)	Ref. No.
0.50	NCM811	PP_13_TFSI	LiPF_6_/LiTFSI/LiBOB/NaPF_6_/PC/PP_13_TFSI/TEP/FEC	4.50	4.0	50	Not mentioned	~204	250	~78.4	~0.086	[127]
0.50	LNMO	PP_13_DFOB	LiTFSI/PP_13_DFOB/DMC	4.90	1.6	>200	50	~127	100	~85.8	~0.142	[128]
0.20	LCO	Pyr_1,3_FSI	LiTFSI/Pyr_1,3_FSI	4.40	1.6	500	Not mentioned	~157	60	~59.2	~0.680	[129]
0.05	NCA	C_3_mpyrFSI	LiFSI/C_3_mpyrFSI/ PDADMA	4.50	8.0	100	100	~171	20	~71.9	~1.405	[130]
0.10	LRNM	P-4444(+)/IM14-	LiFSI/P-4444(+)/IM14-	4.80	2.0	500	100	~250	100	~84.4	~0.156	[131]
0.20	NCM622	[MEMP][TFSI]	LiDFOB/[MEMP][TFSI]/HFE	4.50	5.0	500	Not mentioned	~200	100	~96.0	~0.040	[132]
0.50	NCM811	C_3_mpyrFSI	LiFSI/C_3_mpyrFSI/DME	4.40	6.4	50	20	~200	300	~81.0	~0.063	[133]
0.20	LNMO	[Py_14_]PF_6_	LiPF_6_/EC/DMC/ [Py_14_]PF_6_	5.00	Not mentioned	>200	Not mentioned	~116	150	~94.8	~0.045	[134]
0.50	NCM88	Pyr_14_FSI	LiTFSI/Pyr_14_FSI	4.60	2.8	50	Not mentioned	~210	100	~97.6	~0.024	[135]
1.00	NCM811	EmimFSI	LiFSI/EmimFSI/BTFE	4.40	10.0	Not mentioned	75	~198	200	~96.0	~0.020	[136]
1.00	NCM811	EmimFSI	LiFSI/EmimFSI/dFBn	4.40	10.0	500	75	~200	500	~93.0	~0.014	[137]

^∗^Abbreviation: PP_13_TFSI = N-methyl-N-propylpiperidinium bis(trifluoromethane-sulfonyl)imide; PP_13_DFOB = N-propyl-N-methylpiperidiniumdifluoro(oxalate)borate; C_3_mpyrFSI = N-methyl-N-propylpyrrolidinium bis(fluorosulfonyl)imide; P-4444(+) = symmetric tetra-butyl-phosphonium; IM14- = (nonafluorobutanesulfonyl)(trifluoromethanesulfonyl)imide; [MEMP][TFSI] = N-methyl-N-methoxyethyl-pyrrolidinium bis(trifluoromethylsulfonyl)imide; C_3_mpyrFSI = N-propylpyrrolidinium bis(fluorosulfonyl)imide; [Py_14_]PF_6_ = 1-butyl-1-methylpyrrolidinium hexafluorophosphate; Pyr_14_FSI = 1-butyl-1-methylpyrrolidinium bis(fluorosulfonyl)imide; EmimFSI = 1-ethyl-3-methylimidazolium bis(fluorosulfonyl)imide.

**Table 7:** The cycling performance of the HVLMBs with different sulfone electrolytes.

Rate (C)	Cathode	Sulfone solvent	Electrolyte	Charging cut-off voltage (V)	Cathode loading (mg cm^−2^)	Lithium metal thickness (*μ*m)	Electrolyte amount (*μ*L)	Initial capacity (mAh g^−1^)	Cycle number	Retention (%)	Decay per cycle (%)	Ref. No.
1.00	LCO	MSM	LiPF_6_/DMC/FEC/HFE/MSM	4.55	12.4	>200	Not mentioned	~200	300	~74.9	~0.084	[145]
0.50	LCO	TMS	LiClO_4_/PVDF/PVAC/TMS	4.50	1.5	>200	10	~195	200	~85.0	~0.075	[141]
0.50	NCM811	SL	LiTFSI/SL/LiNO_3_	4.40	10.0	50	Not mentioned	~190.4	200	~99.5	~0.003	[144]
0.10	NCA	SL	LiODFB/SL/DMC	4.40	3.0	500	50	~205	50	~61.5	~0.770	[146]
0.50	LiNi_0.43_Mn_1.5_Cr_0.07_O_4_	SL	LiPF_6_/SL/DMC	4.80	2.6	500	50	~125	90	~95.1	~0.054	[147]
0.50	NCM811	TMS	LiTFSI/FEC/TMS	4.40	1.3	400	45	~197	500	~86.1	~0.028	[148]
1.00	NCM523	TMS	LiTFSI/LiDFOB/TMS/EA/FEC	4.60	5.0	500	75	~180	200	~89.0	~0.055	[19]
0.50	NCM811	SL	LiTFSI/SL/HFE/FEC	4.70	3.0-4.0	400	50	~200	150	~85.0	~0.100	[149]
1.00	LNMO	MPS	LiPF_6_/EC/DMC/MPS	4.90	2.4-2.6	>200	Not mentioned	~136	400	~89.8	~0.026	[150]
0.05	NCM622	FPES	LiPF_6_/EC/FPES	4.40	2.0	>200	Not mentioned	~110	100	~73.0	~0.270	[151]

^∗^Abbreviation: MPS = methyl phenyl sulfone; FPES = fluorinated multiblock poly(arylene ether sulfone).

**Table 8:** The cycling performance of the HVLMBs with different nitrile electrolytes.

Rate (C)	Cathode	Nitrile solvent	Electrolyte	Charging cut-off voltage (V)	Cathode loading (mg cm^−2^)	Lithium metal thickness (*μ*m)	Electrolyte amount (*μ*L)	Initial capacity (mAh g^−1^)	Cycle number	Retention (%)	Decay per cycle (%)	Ref. No.
1.00	LLO	HTCN	LiPF_6_/EC/DEC/TMSP/HTCN	4.60	Not mentioned	>200	Not mentioned	~183	200	~93.8	~0.031	[152]
0.50	NCM622	AN	LiFSI/AN/VC	4.40	21.6	500	70	~184	200	~59.8	~0.201	[153]
1.00	LCO	SN	LiTFSI/LiDFOB/SN	4.70	24.0	250	Not mentioned	~180	500	~70.0	~0.060	[154]
0.20	NCM811	TmdSx-CN	LiFSI/TmdSx-CN/FEC/LiDFOB	4.50	4.0	20	80	~225	40	~89.0	~0.275	[155]
0.50	LNMO	PVN	LiPF_6_/PVN/GPE	5.00	5.0	500	100	~132	200	~90.0	~0.050	[156]
1.00	LCO	LBTB	LiPF_6_/EC/DEC/EMC/LBTB	4.40	1.9-2.3	500	Not mentioned	~171	300	~73.2	~0.089	[157]
1.00	NCM622	PN	LiTFSI/PN/FEC	4.50	3.0	57	40	~179	300	~75.3	~0.082	[158]
0.10	NCM532	SN	LiTFSI/SN/FEC/PEO	4.40	Not mentioned	500	Not mentioned	~169	120	~80.0	~0.167	[159]
1.00	LCO	GTCE	LiPF_6_/EC/DEC/EMC/GTCE	4.50	2.0	>200	Not mentioned	~190	150	~79.3	~0.138	[160]
1.00	LCO	ACTN	LiPF_6_/EC/EMC/ATCN	4.50	2.3-2.6	>200	50	~173	200	~91.0	~0.045	[161]

^∗^Abbreviation: SN = succinonitrile; TmdSx-CN = 1,3-bis(cyanopropyl)tetramethyl disiloxane; PVN = poly(vinylene carbonate-acrylonitrile); LBTB = lithium 4-benzonitrile trimethyl borate; PN = pivalonitrile; GTCE = glycerol tris(2-cyanoethyl) ether; ACTN = 5-acetylthiophene-2-carbonitrile.

**Table 9:** The cycling performance of the HVLMBs with different SSEs.

Rate (C)	Cathode	Solid-state electrolyte	Electrolyte category	Charging cut-off voltage (V)	Cathode loading (mg cm^−2^)	Lithium metal thickness (*μ*m)	Electrolyte thickness (*μ*m)	Initial capacity (mAh g^−1^)	Cycle number	Retention (%)	Decay per cycle (%)	Ref. No.
1.00	LCO	LiTFSI/PC/PMM-CPE	QSSE	4.45	1.0	400	80	~178	700	~85.0	~0.021	[164]
1.00	NCM622	LiFSI/PVH/PP_12_FSI	ISSE	4.50	2.0-2.5	>200	149	~153	100	~89.2	~0.108	[168]
0.50	NCM523	LiClO_4_/PVDF/LEP	ISSE	4.50	Not mentioned	>200	Not mentioned	~140	100	~99.0	~0.010	[169]
0.50	NCM622	LiTFSI/PQ/TPU	QSSE	4.40	3.0	500	150	~193	100	~75.1	~0.249	[170]
0.20	LNMO	LiDFOB/PFEC	ISSE	4.90	2.9	>200	70	~110	50	~81.8	~0.364	[171]
0.50	LCO	P(CUMA-NPF_6_)-GPE	QSSE	4.45	2.0	100	Not mentioned	~181	500	~87.4	~0.025	[172]
1.00	LCO	LiTFSI/PVDF-HFP/LLZO	ISSE	4.50	Not mentioned	>200	11	~169	100	~89.4	~0.106	[173]
0.10	NCM622	LiTFSI/PVDF-HFP/LLZTO	ISSE	4.50	3.0	500	25	~194	50	~99.4	~0.120	[174]
0.50	LCO	LiPVFM/LiTFSI/DMSO	QSSE	4.45	3.0	>200	70	~177	225	~89.2	~0.048	[175]
0.50	NCM811	LiTFSI/THF/Q-COFs	QSSE	4.40	2.0	20	50	~160	400	~82.0	~0.045	[176]
0.50	LCO	Zn(TFSI)_2_/LiDFOB/SN/DOL	QSSE	4.45	2.0-3.0	>200	Not mentioned	~130	700	~89.0	~0.016	[177]

^∗^Abbreviation: PVH = poly(vinylidene fluoride-co-hexafluopropylene); PP_12_FSI = N-ethyl-N-methylpiperidine bis(fluorosulfonyl)imide; LEP = lepidolite filler; PQ = polyacene quinone; TPU = thermoplastic polyurethane; PFEC = poly(fluoroethylene carbonate); PVDF-HFP = polyvinylidene fluoride-hexafluoropropylene; LLZTO = Li_6.4_La_3_Zr_1.4_Ta_0.6_O_12_; LiPVFM = lithiated polyvinyl formal; Q-COFs = quinolyl-linked covalent organic frameworks; THF = tetrahydrofuran; DOL = 1,3-dioxolane.

**Table 10:** The important properties of representative electrolytes.

Electrolyte composition	Category	Potential window (V)	Melting point for key lithium salt/additive/solvent (°C)	Boiling point for key lithium salt/additive/solvent (°C)	Conductivity (mS cm^−1^)	Viscosity (mPa S)	Contact angle for separator (°)	Cathode compatibility	Ref. No.
LiFSA/DMC	HCE	5.2	300	—	1.1	238.9	Not mentioned	LNMO	[58]
LiFSI/EC/DMC	HCE	5.5	124	—	1.7	Not mentioned	Not mentioned	NMC622	[57]
LiTFSI/[C_2_mpyr][FSI]/DMC	HCE	5.2	234	—	1.4	185.3	Not mentioned	NMC532/LCO	[67]
LiFSI/PC/FEC	HCE	4.8	124	—	6.7	Not mentioned	Not mentioned	NMC811/LCO	[69]
LiFSI/DME/TTE	LHCE	4.6	—	92	2.4	4.8	27	NMC811	[60]
LiFSI/TEP/BTFE	LHCE	4.8	25	62	1.3	2.9	Not mentioned	NMC622/NCA/LCO	[63]
LiDFOB/LiNO_3_/TMP/TTE	LHCE	4.6	—	92	1.4	5.1	15	NMC87/LCO	[71]
LiDFOB/LiBF_4_/DEC/FEC/FB	LHCE	4.8	-42	85	1.3	1.3	22	LCO/NMC532	[62]
LiPF_6_/EC/EMC/DEC/TMB	CEI additive	5.0	-34	67	7.9	Not mentioned	Not mentioned	LRO/LCO	[84]
LiTFSI/LiBOB/LiPF_6_/EC/EMC	CEI additive	4.7	200	—	Not mentioned	Not mentioned	35	NMC76/NMC811	[86]
LiPF_6_/FEC/EMC/TPFPB/LiNO_3_	CEI additive	4.6	126	327	Not mentioned	2.8	37	LCO/NMC811/LNMO	[88]
LiPF_6_/EC/DMC/DEPP	CEI additive	4.7	—	110	Not mentioned	3.1	Not mentioned	LCO/NMC532	[93]
LiPF_6_/EC/EMC/AIP	CEI additive	4.5	128	130	8.3	Not mentioned	Not mentioned	NMC811/LCO	[94]
LiPF_6_/PC/EC/DEC/CTAC	SEI additive	4.5	232	—	Not mentioned	4.7	19	NMC532	[100]
LiPF_6_/EC/EMC/HFiP	SEI additive	4.7	32	72	7.7	Not mentioned	Not mentioned	LNMO/LLO	[102]
LiTFSI/FEC/TFEO	SEI additive	4.5	—	143	3.2	4.9	17	NMC811	[97]
LiPF_6_/EC/DMC/MSIPE/HFPTF	SEI additive	4.4	—	102	Not mentioned	3.7	Not mentioned	NMC811/LCO	[105]
LiPF_6_/FEC/MTFP	Fluorinated electrolyte	5.8	—	95	Not mentioned	2.1	Not mentioned	NMC811/LCO	[114]
LiFSI/DME/TFEO	Fluorinated electrolyte	5.2	—	144	15.8	4.9	21	NMC811	[23]
LiPF_6_/FEC/FEMC/HFE	Fluorinated electrolyte	5.5	-91	56	5.1	Not mentioned	Not mentioned	NMC811/LCP	[54]
LiN(SO_2_F)_2_/FEMC/TFEP	Fluorinated electrolyte	4.8	—	121	2.0	6.3	Not mentioned	LNMO/NMC333	[21]
LiFSI/F5DEE	Fluorinated electrolyte	5.2	—	135	5.1	3.4	18	NMC532/NMC622/NMC811	[120]
LiFSI/dFBn/ EmimFSI	Ionic-liquid electrolyte	5.0	-18	—	8.8	24.7	Not mentioned	NMC811	[137]
LiFSI/NaTFSI/ EmimFSI	Ionic-liquid electrolyte	4.6	-18	—	3.3	125.0	Not mentioned	LCO/NMC811	[56]
LiTFSI/Pyr_14_FSI	Ionic-liquid electrolyte	5.0	-19	—	3.1	81.0	Not mentioned	NMC88	[135]
LiFSI/DME/C_3_mpyrFSI	Ionic-liquid electrolyte	5.5	-9	—	2.4	92.0	Not mentioned	NMC811/NMC622	[133]
LiTFSI/FEC/TMS	Sulfone electrolyte	5.2	27	285	10.1	8.3	29	NMC811/LCO	[148]
LiPF_6_/DMC/FEC/HFE/MSM	Sulfone electrolyte	5.5	107	238	12.5	9.7	Not mentioned	LCO/NMC532	[145]
LiPF_6_/EC/DMC/MPS	Sulfone electrolyte	4.5	85	303	9.6	Not mentioned	35	LNMO/LCO	[150]
LiPF_6_/EC/FPES	Sulfone electrolyte	4.9	288	—	10.0	Not mentioned	Not mentioned	NMC622	[151]
LiPF_6_/EC/DEC/TMSP/HTCN	Nitrile electrolyte	4.8	—	255	5.1	Not mentioned	37	LLO	[152]
LiFSI/VC/AN	Nitrile electrolyte	5.5	-45	81	16.0	7.2	26	NMC333/NMC622/NCA	[153]
LiTFSI/LiDFOB/SN	Nitrile electrolyte	5.0	50	265	1.3	753.0	Not mentioned	LCO/NMC532	[154]
LiPF_6_/EC/EMC/ATCN	Nitrile electrolyte	4.9	77	95	Not mentioned	Not mentioned	45	LCO/LMO	[161]
LiTFSI/PC/PMM-CPE	QSSE	5.3	—	202	1.3	—	—	LCO	[164]
LiTFSI/PQ/TPU	QSSE	4.8	—	136	5.0	—	—	NMC622	[170]
P(CUMA-NPF_6_)-GPE	QSSE	5.6	—	338	1.0	—	—	LCO/LNMO	[172]
LiPVFM/LiTFSI/DMSO	QSSE	5.0	120	—	0.4	—	—	LCO	[175]
LiFSI/PP_12_FSI/PVH	ISSE	5.3	135	—	0.5	—	—	NMC622	[168]
LiDFOB/PFEC	ISSE	5.5	190	—	0.2	—	—	LCO/LNMO	[171]
LiTFSI/LLZO/PVDF-HFP	ISSE	4.5	135	—	0.1	—	—	LCO	[173]

**Table 11:** The advantages and disadvantages of eight improvement strategies for HVLMBs.

Strategies	Parameters				
	Rate capacity	Safety	Interface formation	Antioxidation stability	Cost
High concentration electrolytes	Lower	Better	Better	Good	Highest
Additives with CEI formation	Fair	Fair	Best	Good	High
Additives with SEI formation	Fair	Good	Best	Fair	High
Fluorinated electrolytes	Good	Better	Best	Good	Higher
Ionic-liquid electrolytes	Low	Good	Good	Better	Higher
Sulfone electrolytes	Fair	Fair	Lower	Better	Fair
Nitrile electrolytes	Good	Good	Lower	Best	Fair
Solid-state electrolytes	Lowest	Best	Fair	Good	Highest

^∗^The criterion of the grade assessment is based on the classic commercial electrolyte (1 M LiPF_6_ in EC/DMC, *v*/*v* = 1 : 1).

## References

[1] Chi X., Li M., Di J. (2021). A highly stable and flexible zeolite electrolyte solid-state Li-air battery. *Nature*.

[2] Yuan B. T., Wen K. C., Chen D. J. (2021). Composite separators for robust high rate lithium ion batteries. *Advanced Functional Materials*.

[3] Dong L., Liu Y., Wen K. (2021). High-polarity fluoroalkyl ether electrolyte enables solvation-free Li^+^ transfer for high-rate lithium metal batteries. *Advanced Science*.

[4] Wang P. F., Meng Y., Wang Y. J. (2022). Oxygen framework reconstruction by LiAlH_4_ treatment enabling stable cycling of high-voltage LiCoO_2_. *Energy Storage Materials*.

[5] An H. W., Liu Q. S., An J. L. (2021). Coupling two-dimensional fillers with polymer chains in solid polymer electrolyte for room-temperature dendrite-free lithium-metal batteries. *Energy Storage Materials*.

[6] Dong L. W., Liu J. P., Chen D. J. (2019). Suppression of polysulfide dissolution and shuttling with glutamate electrolyte for lithium sulfur batteries. *ACS Nano*.

[7] Zhong S. J., Yuan B. T., Guang Z. X. (2021). Recent progress in thin separators for upgraded lithium ion batteries. *Energy Storage Materials*.

[8] Qiao Y., Yang H. J., Chang Z., Deng H., Li X., Zhou H. (2021). A high-energy-density and long-life initial-anode-free lithium battery enabled by a Li_2_O sacrificial agent. *Nature Energy*.

[9] Lv Q., Jiang Y., Wang B. (2022). Suppressing lithium dendrites within inorganic solid-state electrolytes. *Cell Reports Physical Science*.

[10] Holoubek J., Liu H. D., Wu Z. H. (2021). Tailoring electrolyte solvation for Li metal batteries cycled at ultra-low temperature. *Nature Energy*.

[11] Xue W. J., Huang M. J., Li Y. T. (2021). Ultra-high-voltage Ni-rich layered cathodes in practical Li metal batteries enabled by a sulfonamide-based electrolyte. *Nature Energy*.

[12] Boyle D. T., Huang W., Wang H. S. (2021). Corrosion of lithium metal anodes during calendar ageing and its microscopic origins. *Nature Energy*.

[13] Chen H., Yang Y. F., Boyle D. T. (2021). Free-standing ultrathin lithium metal-graphene oxide host foils with controllable thickness for lithium batteries. *Nature Energy*.

[14] Liu J., Bao Z. N., Cui Y. (2019). Pathways for practical high-energy long-cycling lithium metal batteries. *Nature Energy*.

[15] Liu J. P., Dong L. W., Chen D. J. (2020). Metal oxides with distinctive valence states in an electron-rich matrix enable stable high-capacity anodes for Li ion batteries. *Small Methods*.

[16] Wang Z. Y., Shen L., Deng S. G., Cui P., Yao X. (2021). 10 *μ*m‐Thick high-strength solid polymer electrolytes with excellent interface compatibility for flexible all-solid-state lithium-metal batteries. *Advanced Materials*.

[17] Zou Y. G., Cao Z., Zhang J. L. (2021). Interfacial model deciphering high-voltage electrolytes for high energy density, high safety, and fast-charging lithium-ion batteries. *Advanced Materials*.

[18] Wang X. S., Wang S. W., Wang H. R. (2021). Hybrid electrolyte with dual-anion-aggregated solvation sheath for stabilizing high-voltage lithium-metal batteries. *Advanced Materials*.

[19] Lin S. S., Hua H. M., Lai P. B., Zhao J. B. (2021). A multifunctional dual-salt localized high-concentration electrolyte for fast dynamic high-voltage lithium battery in wide temperature range. *Advanced Energy Materials*.

[20] Roy B., Cherepanov P., Nguyen C. (2021). Lithium borate ester salts for electrolyte application in next-generation high voltage lithium batteries. *Advanced Energy Materials*.

[21] Zheng Q. F., Yamada Y., Shang R. (2020). A cyclic phosphate-based battery electrolyte for high voltage and safe operation. *Nature Energy*.

[22] Zheng T. L., Xiong J. W., Shi X. T. (2021). Cocktail therapy towards high temperature/high voltage lithium metal battery *via* solvation sheath structure tuning. *Energy Storage Materials*.

[23] Cao X., Zou L. F., Matthews B. E. (2021). Optimization of fluorinated orthoformate based electrolytes for practical high-voltage lithium metal batteries. *Energy Storage Materials*.

[24] Chen J., Fan X., Li Q. (2020). Electrolyte design for LiF-rich solid-electrolyte interfaces to enable high- performance microsized alloy anodes for batteries. *Nature Energy*.

[25] Luo D., Li M., Zheng Y. (2021). Electrolyte design for lithium metal anode-based batteries toward extreme temperature application. *Advanced Science*.

[26] Ye Y. S., Zhao Y. Y., Zhao T. (2021). An antipulverization and high-continuity lithium metal anode for high-energy lithium batteries. *Advanced Materials*.

[27] Cao Z., Zheng X. Y., Qu Q. T., Huang Y., Zheng H. (2021). Electrolyte design enabling a high-safety and high-performance Si anode with a tailored electrode-electrolyte interphase. *Advanced Materials*.

[28] Huang S. Z., Yang J. F., Ma L. X. (2021). Effectively regulating more robust amorphous Li clusters for ultrastable dendrite-free cycling. *Advanced Science*.

[29] Wang H. P., He J., Liu J. D. (2021). Electrolytes enriched by crown ethers for lithium metal batteries. *Advanced Functional Materials*.

[30] Wang Q., Wan J., Cao X. (2022). Organophosphorus hybrid solid electrolyte interphase layer based on LixPO4Enables uniform lithium deposition for high-performance lithium metal batteries. *Advanced Functional Materials*.

[31] Chen C., Liang Q. W., Wang G., Liu D., Xiong X. (2022). Grain-boundary-rich artificial SEI layer for high-rate lithium metal anodes. *Advanced Functional Materials*.

[32] Liu Q. Q., Chen Z. R., Liu Y. (2021). Cooperative stabilization of bi-electrodes with robust interphases for high- voltage lithium-metal batteries. *Energy Storage Materials*.

[33] Xiao C. F., Kim J. H., Cho S. H. (2021). Ensemble design of electrode-electrolyte interfaces: toward high-performance thin-film all-solid-state Li-metal batteries. *ACS Nano*.

[34] Beheshti S. H., Javanbakht M., Omidvar H. (2022). Development, retainment, and assessment of the graphite-electrolyte interphase in Li-ion batteries regarding the functionality of SEI-forming additives. *iScience*.

[35] Xu Z. X., Yang J., Li H. P., Nuli Y., Wang J. (2019). Electrolytes for advanced lithium ion batteries using silicon-based anodes. *Journal of Materials Chemistry A*.

[36] Wang H. S., Yu Z., Kong X. (2022). Liquid electrolyte: the nexus of practical lithium metal batteries. *Joule*.

[37] Liu Y. T., Elias Y., Meng J. S. (2021). Electrolyte solutions design for lithium-sulfur batteries. *Joule*.

[38] Nanda S., Gupta A., Manthiram A. (2021). Anode-free full cells: a pathway to high-energy density lithium-metal batteries. *Advanced Energy Materials*.

[39] von Aspern N., Roeschenthaler G. V., Winter M., Cekic-Laskovic I. (2019). Fluorine and lithium: ideal partners for high-performance rechargeable battery electrolytes. *Angewandte Chemie-International Edition*.

[40] Cao X., Jia H., Xu W., Zhang J. G. (2021). Review-localized high-concentration electrolytes for lithium batteries. *Journal of The Electrochemical Society*.

[41] Han J. G., Kim K., Lee Y., Choi N. S. (2019). Scavenging materials to stabilize LiPF_6_-containing carbonate-based electrolytes for Li-ion batteries. *Advanced Materials*.

[42] Borodin O., Smith G. D. (2006). Development of many-body polarizable force fields for Li-battery components: 1. Ether, alkane, and carbonate-based solvents. *Journal of Physical Chemistry B*.

[43] Zhou W. J., Zhang M., Kong X. Y., Huang W., Zhang Q. (2021). Recent advance in ionic-liquid-based electrolytes for rechargeable metal-ion batteries. *Advanced Science*.

[44] Gond R., van Ekeren W., Mogensen R., Naylor A. J., Younesi R. (2021). Non-flammable liquid electrolytes for safe batteries. *Materials Horizons*.

[45] Zhao H. J., Yu X. Q., Li J. D. (2019). Film-forming electrolyte additives for rechargeable lithium-ion batteries: progress and outlook. *Journal of Materials Chemistry A*.

[46] Hubble D., Brown D. E., Zhao Y. Z. (2022). Liquid electrolyte development for low-temperature lithium-ion batteries. *Energy & Environmental Science*.

[47] Hayashi K., Nemoto Y., Tobishima S., Yamaki J. (1999). Mixed solvent electrolyte for high voltage lithium metal secondary cells. *Electrochimica Acta*.

[48] Seki S., Kobayashi Y., Miyashiro H., Usami A., Mita Y., Terada N. (2006). Improvement in high-voltage performance of all-solid-state lithium polymer secondary batteries by mixing inorganic electrolyte with cathode materials. *Journal of The Electrochemical Society*.

[49] Cho J. H., Park J. H., Lee M. H., Song H. K., Lee S. Y. (2012). A polymer electrolyte-skinned active material strategy toward high-voltage lithium ion batteries: a polyimide-coated LiNi_0.5_Mn_1.5_O_4_ spinel cathode material case. *Energy & Environmental Science*.

[50] Li Z. D., Zhang Y. C., Xiang H. F. (2013). Trimethyl phosphite as an electrolyte additive for high-voltage lithium-ion batteries using lithium-rich layered oxide cathode. *Journal of Power Sources*.

[51] Qian J. F., Henderson W. A., Xu W. (2015). High rate and stable cycling of lithium metal anode. *Nature Communications*.

[52] Wu F., Zhou H., Bai Y., Wang H., Wu C. (2015). Toward 5 V Li-ion batteries: quantum chemical calculation and electrochemical characterization of sulfone-based high-voltage electrolytes. *ACS Applied Materials & Interfaces*.

[53] Kim H. W., Manikandan P., Lim Y. J., Kim J. H., Nam S. C., Kim Y. (2016). Hybrid solid electrolyte with the combination of Li7La3Zr2O12ceramic and ionic liquid for high voltage pseudo-solid-state Li-ion batteries. *Journal of Materials Chemistry A*.

[54] Fan X. L., Chen L., Borodin O. (2018). Non-flammable electrolyte enables Li-metal batteries with aggressive cathode chemistries. *Nature Nanotechnology*.

[55] Lee S. H., Hwang J. Y., Park S. J., Park G. T., Sun Y. K. (2019). Adiponitrile (C_6_H_8_N_2_): a new bi-functional additive for high-performance Li-metal batteries. *Advanced Functional Materials*.

[56] Sun H., Zhu G. Z., Zhu Y. M. (2020). High-safety and high-energy-density lithium metal batteries in a novel ionic-liquid electrolyte. *Advanced Materials*.

[57] Fan X. L., Chen L., Ji X. (2018). Highly fluorinated interphases enable high-voltage Li-metal batteries. *Chem*.

[58] Wang J. H., Yamada Y., Sodeyama K., Chiang C. H., Tateyama Y., Yamada A. (2016). Superconcentrated electrolytes for a high-voltage lithium-ion battery. *Nature Communications*.

[59] Zhang X. H., Zou L. F., Xu Y. B. (2020). Advanced electrolytes for fast-charging high-voltage lithium-ion batteries in wide-temperature range. *Advanced Energy Materials*.

[60] Ren X. D., Zou L. F., Cao X. (2019). Enabling high-voltage lithium-metal batteries under practical conditions. *Joule*.

[61] Yoo D. J., Yang S., Kim K. J., Choi J. W. (2020). Fluorinated aromatic diluent for high-performance lithium metal batteries. *Angewandte Chemie-International Edition*.

[62] Jiang Z. P., Zeng Z. Q., Zhang H. (2022). Low concentration electrolyte with non-solvating cosolvent enabling high- voltage lithium metal batteries. *iScience*.

[63] Chen S. R., Zheng J. M., Yu L. (2018). High-efficiency lithium metal batteries with fire-retardant electrolytes. *Joule*.

[64] Jiang G. X., Li F., Wang H. P. (2021). Perspective on high-concentration electrolytes for lithium metal batteries. *Small Structures*.

[65] Hu J. T., Ji Y. C., Zheng G. R. (2022). Influence of electrolyte structural evolution on battery applications: cationic aggregation from dilute to high concentration. *Aggregate*.

[66] Chen X., Zhang Q. (2020). Atomic insights into the fundamental interactions in lithium battery electrolytes. *Accounts of Chemical Research*.

[67] Wang Z. C., Sun Y. Y., Mao Y. Y. (2020). Highly concentrated dual-anion electrolyte for non-flammable high-voltage Li- metal batteries. *Energy Storage Materials*.

[68] Wang W., Zhang J. L., Yang Q., Wang S., Wang W., Li B. (2020). Stable cycling of high-voltage lithium-metal batteries enabled by high-concentration FEC-based electrolyte. *ACS Applied Materials & Interfaces*.

[69] Cho S. J., Yu D. E., Pollard T. P. (2020). Nonflammable lithium metal full cells with ultra-high energy density based on coordinated carbonate electrolytes. *iScience*.

[70] Chang Z., Qiao Y., Yang H. J. (2021). Sustainable lithium-metal battery achieved by a safe electrolyte based on recyclable and low-cost molecular sieve. *Angewandte Chemie-International Edition*.

[71] Jia M., Zhang C., Guo Y. (2022). Advanced nonflammable localized high-concentration electrolyte for high energy density lithium battery. *Energy & Environmental Materials*.

[72] Wang X. F., He W. J., Xue H. L. (2022). A nonflammable phosphate-based localized high-concentration electrolyte for safe and high-voltage lithium metal batteries. *Sustainable Energy & Fuels*.

[73] Jang J., Sugimoto T., Mizumo T., Lee J. M., Chang W. S., Mun J. (2021). High-voltage-compatible dual-ether electrolyte for lithium metal batteries. *ACS Applied Energy Materials*.

[74] Niu C. J., Lee H., Chen S. R. (2019). High-energy lithium metal pouch cells with limited anode swelling and long stable cycles. *Nature Energy*.

[75] Niu C. J., Liu D. Y., Lochala J. A. (2021). Balancing interfacial reactions to achieve long cycle life in high-energy lithium metal batteries. *Nature Energy*.

[76] Wang X. Y., Li S. Y., Zhang W. D. (2021). Dual-salt-additive electrolyte enables high-voltage lithium metal full batteries capable of fast-charging ability. *Nano Energy*.

[77] Xiao P. T., Zhao Y., Piao Z. H., Li B., Zhou G., Cheng H. M. (2022). A nonflammable electrolyte for ultrahigh-voltage (4.8 V-class) Li||NCM811 cells with a wide temperature range of 100 °C. *Energy & Environmental Science*.

[78] Choudhury S., Tu Z. Y., Nijamudheen A. (2019). Stabilizing polymer electrolytes in high-voltage lithium batteries. *Nature Communications*.

[79] Yue H. Y., Yang Y. E., Xiao Y. (2019). Boron additive passivated carbonate electrolytes for stable cycling of 5 V lithium-metal batteries. *Journal of Materials Chemistry A*.

[80] Yoon T., Park S., Mun J. (2012). Failure mechanisms of LiNi_0.5_Mn_1.5_O_4_ electrode at elevated temperature. *Journal of Power Sources*.

[81] Li J. Y., Li W. D., You Y., Manthiram A. (2018). Extending the service life of high-Ni layered oxides by tuning the electrode-electrolyte interphase. *Advanced Energy Materials*.

[82] Deng T., Fan X., Cao L. (2019). Designing *in-situ*-formed interphases enables highly reversible cobalt-free LiNiO_2_ cathode for Li-ion and Li-metal batteries. *Joule*.

[83] Li Y. X., Li W. K., Shimizu R. (2022). Elucidating the effect of borate additive in high-voltage electrolyte for Li-rich layered oxide materials. *Advanced Energy Materials*.

[84] Li J. H., Liao Y. Q., Fan W. Z. (2020). Significance of electrolyte additive molecule structure in constructing robust interphases on high-voltage cathodes. *ACS Applied Energy Materials*.

[85] Lee T. J., Soon J., Chae S., Ryu J. H., Oh S. M. (2019). A bifunctional electrolyte additive for high-voltage LiNi0.5Mn1.5O4Positive electrodes. *ACS Applied Materials & Interfaces*.

[86] Zhao W. G., Zheng J. M., Zou L. F. (2018). High voltage operation of Ni-rich NMC cathodes enabled by stable electrode/electrolyte interphases. *Advanced Energy Materials*.

[87] Wagner R., Streipert B., Kraft V. (2016). Counterintuitive role of magnesium salts as effective electrolyte additives for high voltage lithium-ion batteries. *Advanced Materials Interfaces*.

[88] Li S. Y., Zhang W. D., Wu Q. (2020). Synergistic dual-additive electrolyte enables practical lithium-metal batteries. *Angewandte Chemie-International Edition*.

[89] Zhuang Y., Lei Y. Q., Guan M. Y. (2020). 4-Aminobenzoic acid as a novel electrolyte additive for improved electrochemical performance of Li_1.2_Ni_0.2_Mn_0.6_O_2_ cathodes via _in situ_ electrochemical polymerization. *Electrochimica Acta*.

[90] Zheng Y., Fang W., Zheng H. (2019). A multifunctional thiophene-based electrolyte additive for lithium metal batteries using high-voltage LiCoO2Cathode. *Journal of The Electrochemical Society*.

[91] Wang Z. S., Xing L. D., Li J. H., Xu M., Li W. (2016). Triethylborate as an electrolyte additive for high voltage layered lithium nickel cobalt manganese oxide cathode of lithium ion battery. *Journal of Power Sources*.

[92] Kazzazi A., Bresser D., Kuenzel M. (2021). Synergistic electrolyte additives for enhancing the performance of high- voltage lithium-ion cathodes in half-cells and full-cells. *Journal of Power Sources*.

[93] Miao C. X., Qi S. H., Liang K. (2021). Diethyl phenylphosphonite contributing to solid electrolyte interphase and cathode electrolyte interphase for lithium metal batteries. *Journal of Energy Chemistry*.

[94] Yang J. X., Liu X., Wang Y. A. (2021). Electrolytes polymerization-induced cathode-electrolyte-interphase for high voltage lithium-ion batteries. *Advanced Energy Materials*.

[95] Wang H. S., Liu Y. Y., Li Y. Z., Cui Y. (2019). Lithium metal anode materials design: interphase and host. *Electrochemical Energy Reviews*.

[96] Xiao D., Li Q., Luo D. (2020). Regulating the Li+‐Solvation structure of ester electrolyte for high-energy-density lithium metal batteries. *Small*.

[97] Cao X., Ren X. D., Zou L. F. (2019). Monolithic solid-electrolyte interphases formed in fluorinated orthoformate- based electrolytes minimize Li depletion and pulverization. *Nature Energy*.

[98] Wang Q., Yao Z., Zhao C. (2020). Interface chemistry of an amide electrolyte for highly reversible lithium metal batteries. *Nature Communications*.

[99] Tan S. J., Yue J., Hu X. C. (2019). Nitriding-interface-regulated lithium plating enables flame-retardant electrolytes for high-voltage lithium metal batteries. *Angewandte Chemie-International Edition*.

[100] Dai H. L., Xi K., Liu X., Lai C., Zhang S. (2018). Cationic surfactant-based electrolyte additives for uniform lithium deposition *via* lithiophobic repulsion mechanisms. *Journal of the American Chemical Society*.

[101] Chen H., Chen J., Zhang W. (2020). Enhanced cycling stability of high-voltage lithium metal batteries with a trifunctional electrolyte additive. *Journal of Materials Chemistry A*.

[102] Yang Y. G., Zhang Z. T., Yue H. Y. (2020). Anti-cognition in lithium-ion battery electrolytes: comparable performance with degraded electrolyte. *Journal of Power Sources*.

[103] Kim J., Pham H. Q., Chung G. J., Hwang E. H., Kwon Y. G., Song S. W. (2021). Impacts of fluorinated phosphate additive on interface stabilization of 4.6 V battery cathode. *Electrochimica Acta*.

[104] Ma X., Feng D. Y., Xiao Y. L. (2021). Generating lithium fluoride-abundant interphase on layered lithium-rich oxide cathode with lithium 1,1,2,2,3,3-hexafluoropropane-1,3-disulfonimide. *Journal of Power Sources*.

[105] Li X., Liu J. D., He J. (2021). Hexafluoroisopropyl trifluoromethanesulfonate-driven easily Li^+^ desolvated electrolyte to afford Li||NCM811 cells with efficient anode/cathode electrolyte interphases. *Advanced Functional Materials*.

[106] Wen Y. C., Ding J. Y., Yang Y. (2022). Introducing NO3–into carbonate-based electrolytes *via* covalent organic framework to incubate stable interface for Li-metal batteries. *Advanced Functional Materials*.

[107] Ma Z. K., Chen H. Y., Zhou H. B., Xing L., Li W. (2021). Cost-efficient film-forming additive for high-voltage lithium-nickel-manganese oxide cathodes. *ACS Omega*.

[108] Qi S. H., Wang H. P., He J. (2021). Electrolytes enriched by potassium perfluorinated sulfonates for lithium metal batteries. *Science Bulletin*.

[109] Yu Z., Wang H. S., Kong X. (2020). Molecular design for electrolyte solvents enabling energy-dense and long- cycling lithium metal batteries. *Nature Energy*.

[110] Amanchukwu C. V., Yu Z., Kong X., Qin J., Cui Y., Bao Z. (2020). A new class of ionically conducting fluorinated ether electrolytes with high electrochemical stability. *Journal of the American Chemical Society*.

[111] Zhao H., Gu J., Gao Y. (2020). A multifunctional electrolyte with highly-coordinated solvation structure-in- nonsolvent for rechargeable lithium batteries. *Journal of Energy Chemistry*.

[112] Deng W., Dai W., Zhou X. (2021). Competitive solvation-induced concurrent protection on the anode and cathode toward a 400 Wh kg–1Lithium metal battery. *ACS Energy Letters*.

[113] Lavi O., Luski S., Shpigel N. (2020). Electrolyte solutions for rechargeable Li-ion batteries based on fluorinated solvents. *ACS Applied Energy Materials*.

[114] Holoubek J., Yu M. Y., Yu S. C. (2020). An all-fluorinated ester electrolyte for stable high-voltage Li metal batteries capable of ultra-low-temperature operation. *ACS Energy Letters*.

[115] Ren X., Zhang X., Shadike Z. (2020). Designing advanced *in situ* electrode/electrolyte interphases for wide temperature operation of 4.5 V Li||LiCoO2Batteries. *Advanced Materials*.

[116] Pham H. Q., Hwang E. H., Kwon Y. G., Song S. W. (2019). Approaching the maximum capacity of nickel-rich LiNi0.8Co0.1Mn0.1O2cathodes by charging to high-voltage in a non-flammable electrolyte of propylene carbonate and fluorinated linear carbonates. *Chemical Communications*.

[117] Chen L., Fan X. L., Hu E. Y. (2019). Achieving high energy density through increasing the output voltage: a highly reversible 5.3 V battery. *Chem*.

[118] Yu Z., Rudnicki P. E., Zhang Z. W. (2022). Rational solvent molecule tuning for high-performance lithium metal battery electrolytes. *Nature Energy*.

[119] Su C. C., He M. N., Cai M. (2022). Solvation-protection-enabled high-voltage electrolyte for lithium metal batteries. *Nano Energy*.

[120] Zhao Y., Zhou T. H., Ashirov T. (2022). Fluorinated ether electrolyte with controlled solvation structure for high voltage lithium metal batteries. *Nature Communications*.

[121] Zhou T., Zhao Y., El Kazzi M., Choi J. W., Coskun A. (2022). Integrated ring-chain design of a new fluorinated ether solvent for high-voltage lithium-metal batteries. *Angewandte Chemie-International Edition*.

[122] Tan L. J., Chen S. Q., Chen Y. W. (2022). Intrinsic nonflammable ether electrolytes for ultrahigh-voltage lithium metal batteries enabled by chlorine functionality. *Angewandte Chemie-International Edition*.

[123] Wang X. E., Girard G. M. A., Zhu H. J. (2019). Poly (ionic liquid)s/electrospun nanofiber composite polymer electrolytes for high energy density and safe Li metal batteries. *ACS Applied Energy Materials*.

[124] Liu K. X., Wang Z. Y., Shi L. Y., Jungsuttiwong S., Yuan S. (2021). Ionic liquids for high performance lithium metal batteries. *Journal of Energy Chemistry*.

[125] Ishihara Y., Miyazaki K., Fukutsuka T., Abe T. (2014). Lithium-ion transfer at the interface between high potential negative electrodes and ionic liquids. *Journal of The Electrochemical Society*.

[126] Sagane F., Abe T., Ogumi Z. (2012). Electrochemical analysis of lithium-ion transfer reaction through the interface between ceramic electrolyte and ionic liquids. *Journal of The Electrochemical Society*.

[127] Yuan S. Y., Bao J. L., Wang N. (2020). Salt-rich solid electrolyte interphase for safer high-energy-density Li metal batteries with limited Li excess. *Chemical Communications*.

[128] Liang F. X., Yu J. L., Chen J. H. (2018). A novel boron-based ionic liquid electrolyte for high voltage lithium-ion batteries with outstanding cycling stability. *Electrochimica Acta*.

[129] Zhang H. Q., Qu W. J., Chen N. (2018). Ionic liquid electrolyte with highly concentrated LiTFSI for lithium metal batteries. *Electrochimica Acta*.

[130] Girard G. M. A., Wang X., Yunis R., Howlett P. C., Forsyth M. (2020). Stable performance of an all-solid-state Li metal cell coupled with a high-voltage NCA cathode and ultra-high lithium content poly(ionic liquid)s-based polymer electrolyte. *Journal of Solid State Electrochemistry*.

[131] Wu F., Schuer A. R., Kim G. T. (2021). A novel phosphonium ionic liquid electrolyte enabling high-voltage and high- energy positive electrode materials in lithium-metal batteries. *Energy Storage Materials*.

[132] Wang Z., Zhang H., Xu J. (2022). Advanced ultralow-concentration electrolyte for wide-temperature and high-voltage Li-metal batteries. *Advanced Functional Materials*.

[133] Pal U., Rakov D., Lu B. Y. (2022). Interphase control for high performance lithium metal batteries using ether aided ionic liquid electrolyte. *Energy & Environmental Science*.

[134] Poiana R., Lufrano E., Tsurumaki A., Simari C., Nicotera I., Navarra M. A. (2022). Safe gel polymer electrolytes for high voltage Li-batteries. *Electrochimica Acta*.

[135] Wu F. L., Fang S., Kuenzel M. (2021). Dual-anion ionic liquid electrolyte enables stable Ni-rich cathodes in lithium-metal batteries. *Joule*.

[136] Liu X., Mariani A., Zarrabeitia M. (2022). Effect of organic cations in locally concentrated ionic liquid electrolytes on the electrochemical performance of lithium metal batteries. *Energy Storage Materials*.

[137] Liu X., Mariani A., Diemant T. (2022). Difluorobenzene-based locally concentrated Ionic liquid electrolyte enabling stable cycling of lithium metal batteries with nickel-rich cathode. *Advanced Energy Materials*.

[138] Fan X. L., Wang C. S. (2021). High-voltage liquid electrolytes for Li batteries: progress and perspectives. *Chemical Society Reviews*.

[139] Tan S., Ji Y. J., Zhang Z. R., Yang Y. (2014). Recent progress in research on high-voltage electrolytes for lithium-ion batteries. *ChemPhysChem*.

[140] Zhang T., Paillard E. (2018). Recent advances toward high voltage, EC-free electrolytes for graphite-based Li-ion battery. *Frontiers of Chemical Science and Engineering*.

[141] Yu X. R., Wang L. L., Ma J., Sun X., Zhou X., Cui G. (2020). Selectively wetted rigid-flexible coupling polymer electrolyte enabling superior stability and compatibility of high-voltage lithium metal batteries. *Advanced Energy Materials*.

[142] Ren X. D., Chen S. R., Lee H. (2018). Localized High-Concentration Sulfone Electrolytes for High-Efficiency Lithium- Metal Batteries. *Chem*.

[143] Spath T., Fingerle M., Schulz N., Jaegermann W., Hausbrand R. (2016). Adsorption of dimethyl sulfoxide on LiCoO2Thin films: interface formation studied by photoemission spectroscopy. *Journal of Physical Chemistry C*.

[144] Fu J., Ji X., Chen J. (2020). Lithium nitrate regulated sulfone electrolytes for lithium metal batteries. *Angewandte Chemie-International Edition*.

[145] Kong X. B., Zhou R., Wang J., Zhao J. B. (2019). An effective electrolyte strategy to improve the high-voltage performance of LiCoO2Cathode materials. *ACS Applied Energy Materials*.

[146] Zhou H., Yang Z., Xiao D., Xiao K., Li J. (2018). An electrolyte to improve the deep charge-discharge performance of LiNi_0.8_Co_0.15_Al_0.05_O_2_ cathode. *Journal of Materials Science: Materials in Electronics*.

[147] Zhang T., de Meatza I., Qi X., Paillard E. (2017). Enabling steady graphite anode cycling with high voltage, additive-free, sulfolane-based electrolyte: role of the binder. *Journal of Power Sources*.

[148] Dong L. W., Liu Y. P., Chen D. J. (2022). Stabilization of high-voltage lithium metal batteries using a sulfone-based electrolyte with bi-electrode affinity and LiSO_2_F-rich interphases. *Energy Storage Materials*.

[149] Hou W. B., Zhu D. L., Ma S. D., Yang W., Yan H., Dai Y. (2022). High-voltage nickel-rich layered cathodes in lithium metal batteries enabled by a sulfolane / fluorinated ether/ fluoroethylene carbonate-based electrolyte design. *Journal of Power Sources*.

[150] Huang Y. G., Li Y., Tan C. L. (2022). Modifying the cathode-electrolyte interphase by sulfone-based additive to enhance the electrochemical performance of LiNi_0.5_Mn_1.5_O_4_. *ACS Applied Energy Materials*.

[151] Steinle D., Chen Z., Nguyen H. D. (2022). Single-ion conducting polymer electrolyte for _Li||LiNi0.6Mn0.2Co0.2O2_ batteries-impact of the anodic cutoff voltage and ambient temperature. *Journal of Solid State Electrochemistry*.

[152] Zhao J., Liang Y., Zhang X. (2021). *In situ* construction of uniform and robust cathode-electrolyte interphase for Li-rich layered oxides. *Advanced Functional Materials*.

[153] Peng Z., Cao X., Gao P. Y. (2020). High-power lithium metal batteries enabled by high-concentration acetonitrile-based electrolytes with vinylene carbonate additive. *Advanced Functional Materials*.

[154] Hu Z. L., Xian F., Guo Z. Y. (2020). Nonflammable nitrile deep eutectic electrolyte enables high-voltage lithium metal batteries. *Chemistry of Materials*.

[155] Das S. (2020). Highly concentrated nitrile functionalized disiloxane - LiFSI based non-flammable electrolyte for high energy density Li metal battery. *Journal of the Electrochemical Society*.

[156] Wang P., Chai J. C., Zhang Z. H. (2019). An intricately designed poly(vinylene carbonate-acrylonitrile) copolymer electrolyte enables 5 V lithium batteries. *Journal of Materials Chemistry A*.

[157] Sun Z. Y., Zhou H. B., Luo X. H., Che Y., Li W., Xu M. (2021). Design of a novel electrolyte additive for high voltage LiCoO_2_ cathode lithium-ion batteries: lithium 4-benzonitrile trimethyl borate. *Journal of Power Sources*.

[158] Li S. P., Fang S., Li Z. W., Chen W., Dou H., Zhang X. (2022). A high-voltage lithium-metal batteries electrolyte based on fully-methylated pivalonitrile. *Batteries & Supercaps*.

[159] Liu Y. L., Zhao Y., Lu W. (2021). PEO based polymer in plastic crystal electrolytes for room temperature high- voltage lithium metal batteries. *Nano Energy*.

[160] Zhang Z., Huang Z. Y., Liu F. Y. (2021). Glycerol tris(2-cyanoethyl) ether as an electrolyte additive to enhance the cycling stability of lithium cobalt oxide cathode at 4.5V. *ChemElectroChem*.

[161] Ruan D. G., Chen M., Wen X. Y. (2021). *In situ* constructing a stable interface film on high-voltage LiCoO_2_ cathode *via* a novel electrolyte additive. *Nano Energy*.

[162] Zhang H. R., Zhang J. J., Ma J., Xu G., Dong T., Cui G. (2019). Polymer electrolytes for high energy density ternary cathode material-based lithium batteries. *Electrochemical Energy Reviews*.

[163] Sun J., He C., Yao X. (2020). Hierarchical composite-solid-electrolyte with high electrochemical stability and interfacial regulation for boosting ultra-stable lithium batteries. *Advanced Functional Materials*.

[164] Dong T. T., Zhang J. J., Xu G. J. (2018). A multifunctional polymer electrolyte enables ultra-long cycle-life in a high-voltage lithium metal battery. *Energy & Environmental Science*.

[165] Zheng Y. W., Li X. W., Fullerton W. R. (2021). Interpenetrating network-based hybrid solid and gel electrolytes for high voltage lithium metal batteries. *ACS Applied Energy Materials*.

[166] Fan X. L., Ji X., Han F. D. (2018). Fluorinated solid electrolyte interphase enables highly reversible solid-state Li metal battery. *Science Advances*.

[167] Liang J. Y., Zeng X. X., Zhang X. D. (2019). Engineering Janus interfaces of ceramic electrolyte *via* distinct functional polymers for stable high-voltage Li-metal batteries. *Journal of the American Chemical Society*.

[168] Liao Z., Huang J., Chen W. (2020). Safe, superionic conductive and flexible "polymer-in-plastic salts" electrolytes for dendrite-free lithium metal batteries. *Energy Storage Materials*.

[169] Wang B., Wu Y. C., Zhuo S. M. (2020). Synergistic effect of organic plasticizer and lepidolite filler on polymer electrolytes for all-solid high-voltage Li-metal batteries. *Journal of Materials Chemistry A*.

[170] Ye F., Zhang X., Liao K. M. (2020). A smart lithiophilic polymer filler in gel polymer electrolyte enables stable and dendrite-free Li metal anode. *Journal of Materials Chemistry A*.

[171] Liu J., Shen X. W., Zhou J. Q. (2019). Nonflammable and high-voltage-tolerated polymer electrolyte achieving high stability and safety in 4.9 V-class lithium metal battery. *ACS Applied Materials & Interfaces*.

[172] Li X., Han X., Zhang H. (2020). Frontier orbital energy-customized ionomer-based polymer electrolyte for high-voltage lithium metal batteries. *ACS Applied Materials & Interfaces*.

[173] Jiang H., Wu Y. Y., Ma J. (2021). Ultrathin polymer-in-ceramic and ceramic-in-polymer bilayer composite solid electrolyte membrane for high-voltage lithium metal batteries. *Journal of Membrane Science*.

[174] Li X., Cong L. N., Ma S. C. (2021). Low resistance and high stable solid-liquid electrolyte interphases enable high-voltage solid-state lithium metal batteries. *Advanced Functional Materials*.

[175] Li H., Du Y., Wu X., Xie J., Lian F. (2021). Developing "polymer-in-salt" high voltage electrolyte based on composite lithium salts for solid-state Li metal batteries. *Advanced Functional Materials*.

[176] Niu C. Q., Luo W. J., Dai C. M., Yu C., Xu Y. (2021). High-voltage-tolerant covalent organic framework electrolyte with holistically oriented channels for solid-state lithium metal batteries with nickel-rich cathodes. *Angewandte Chemie-International Edition*.

[177] Chen Y., Huo F., Chen S. M., Cai W., Zhang S. (2021). In-built quasi-solid-state poly-ether electrolytes enabling stable cycling of high-voltage and wide-temperature Li metal batteries. *Advanced Functional Materials*.

